# Identification of inhibitors for the transmembrane *Trypanosoma cruzi* eIF2α kinase relevant for parasite proliferation

**DOI:** 10.1016/j.jbc.2023.104857

**Published:** 2023-05-23

**Authors:** Tiago de Paula Marcelino, Angela Maria Fala, Matheus Monteiro da Silva, Normanda Souza-Melo, Amaranta Muniz Malvezzi, Angélica Hollunder Klippel, Martin Zoltner, Norma Padilla-Mejia, Samantha Kosto, Mark C. Field, Gabriela de Assis Burle-Caldas, Santuza Maria Ribeiro Teixeira, Rafael Miguez Couñago, Katlin Brauer Massirer, Sergio Schenkman

**Affiliations:** 1 Departamento de Microbiologia, Imunologia e Parasitologia, Escola Paulista de Medicina, Universidade Federal de São Paulo, São Paulo, SP, Brazil; 2 Center for Molecular Biology and Genetic Engineering – CBMEG, Center of Medicinal Chemistry - CQMED, Structural Genomics Consortium - SGC, University of Campinas - UNICAMP, Campinas, SP, Brazil; 3 Departamento de Ciências Biológicas da Faculdade de Ciências Farmacêuticas da Universidade Estadual Paulista “Júlio de Mesquita Filho”-Unesp, Araraquara, SP, Brazil; 4 Drug Discovery and Evaluation Unit, Department of Parasitology, Faculty of Science, Charles University in Prague, BIOCEV, Vestec, Czech Republic; 5 School of Life Sciences, University of Dundee, Dundee, UK; 6 Biology Centre, Institute of Parasitology, Czech Academy of Sciences, Ceske Budejovice, Czech Republic; 7 Departamento de Bioquímica e Imunologia, Universidade Federal de Minas Gerais, Belo Horizonte, MG, Brazil

**Keywords:** eIF2α, chemical inhibitor, chagas disease, invasion, protein kinase, proteome, recombinant protein, *T.cruzi* EIF2AK2

## Abstract

The TcK2 protein kinase of *Trypanosoma cruzi*, the causative agent of Chagas disease, is structurally similar to the human kinase PERK, which phosphorylates the initiation factor eIF2α and, in turn, inhibits translation initiation. We have previously shown that absence of TcK2 kinase impairs parasite proliferation within mammalian cells, positioning it as a potential target for treatment of Chagas disease. To better understand its role in the parasite, here we initially confirmed the importance of TcK2 in parasite proliferation by generating CRISPR/Cas9 TcK2-null cells, albeit they more efficiently differentiate into infective forms. Proteomics indicates that the TcK2 knockout of proliferative forms expresses proteins including trans-sialidases, normally restricted to infective and nonproliferative trypomastigotes explaining decreased proliferation and better differentiation. TcK2 knockout cells lost phosphorylation of eukaryotic initiation factor 3 and cyclic AMP responsive-like element, recognized to promote growth, likely explaining both decreased proliferation and augmented differentiation. To identify specific inhibitors, a library of 379 kinase inhibitors was screened by differential scanning fluorimetry using a recombinant TcK2 encompassing the kinase domain and selected molecules were tested for kinase inhibition. Only Dasatinib and PF-477736, inhibitors of Src/Abl and ChK1 kinases, showed inhibitory activity with IC50 of 0.2 ± 0.02 mM and 0.8 ± 0.1, respectively. In infected cells Dasatinib inhibited growth of parental amastigotes (IC50 = 0.6 ± 0.2 mM) but not TcK2 of depleted parasites (IC50 > 34 mM) identifying Dasatinib as a potential lead for development of therapeutics for Chagas disease targeting TcK2.

Four protein kinases phosphorylate the α-subunit of the eukaryotic translation initiation factor 2 (eIF2α) in mammals, each being induced by a variety of stresses (1). Among these, the mammalian eIF2α kinase 3, also called protein kinase R of the endoplasmic reticulum kinase (PERK), shows sequence (31.4%) and topological similarity to the protein kinase 2 of eukaryotic translation initiation factor 2 (TcK2) of Trypanosomatids. In mammals, PERK is located in the endoplasmic reticulum (ER) membrane and phosphorylates eIF2α in response to ER stress (2), by releasing the ER chaperone BiP from PERK. The kinase oligomerization leads to enzyme activation, autophosphorylation and phosphorylation of eIF2α, and consequent inhibition of general protein synthesis. This allows preferential translation initiation of mRNAs containing upstream open reading frames, with some of them acting to control stress (3).

*Trypanosoma cruzi* is the agent of Chagas disease, an illness with 30,000 new cases and approximately 12,000 deaths reported per year worldwide. About 8600 newborns become infected during gestation (4) and the options for treatment are restricted to compounds with elevated toxicity (5). Among potential drug targets, 200 kinases have been identified in the *T. cruzi* genome (6), three of which, designated TcK1, TcK2 and TcK3, are predicted to phosphorylate eIF2α. TcK1 is the Gcn2 kinase ortholog, highly conserved in all eukaryotes (7). TcK2 is a PERK homologue possessing both a signal sequence and a transmembrane domain, showing endosomal localization in both *T. cruzi* (8) and *Trypanosoma brucei* (9), rather than the ER. *T. brucei* K2 phosphorylates mammalian eIF2α *in vitro* and acts as an eIF2α kinase when the catalytic domain is expressed in yeast (9). As *Trypanosoma* eIF2α proteins contain an elongated N-terminal domain, phosphorylation occurs at Thr169 instead of the Ser52 in mammalian eIF2α, phosphorylated by specific protein kinases, including PERK. Thr169 phosphorylation is relevant in *T. cruzi* for parasite differentiation (10) and growth inside of mammalian cells (11), and in the absence of TcK2, parasites accumulate reactive oxygen species (ROS) and have slower intracellular proliferation (8).

Current treatments for Chagas disease remain limited, albeit with some recent progress (12, 13). The need to identify new drug modalities remains critical. TcK2 represents a potential new target, given the central role in the intracellular life cycle stages of the parasite (12, 13, 14). With this aim we initially verified that it is effectively expressed in intracellular amastigote, the parasitic stage that should be target for new inhibitors. For this, we expressed a recombinant protein encompassing the kinase domain of TcK2, which was used to prepare highly specific antibodies and to build a platform for TcK2 inhibitor screening. We also reevaluated the requirement of TcK2 for parasite growth by using a new knockout strategy (8), which was required to confirm the target specificity and to provide clues to why the kinase was important for parasite growth, using proteomic analysis. With these approaches, we identified lead inhibitors against TcK2 that acted effectively in mammalian cells infected with *T. cruzi* and that proved to target TcK2, a kinase required for parasite proliferation.

## Results

### Recombinant expression and purification of TcK2 kinase domain

**Figure S1:**
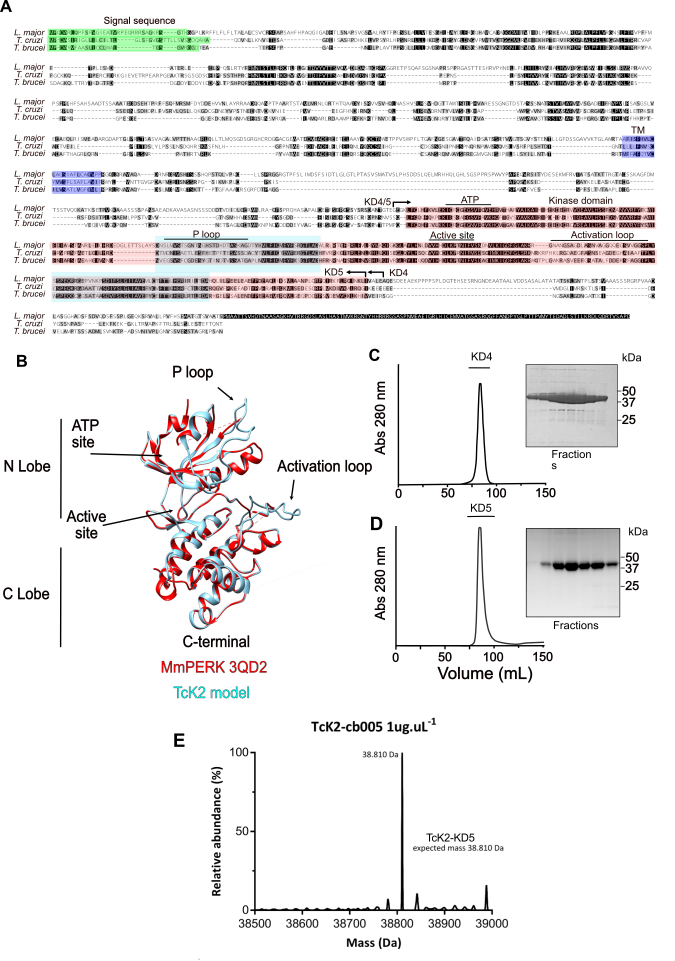
**Conservation of trypanosomatid PERK-like enzymes and expression of soluble recombinant catalytic domain of *T. cruzi* K2.** (A) MUSCLE 3.8.45 alignment of *T. cruzi* (TcYC6_0089220), *T. brucei* (Tb427.04.2500 2), and *Leishmania major* (LmjF.34.2150) obtained from https://tritrypdb.org. The green and purple shade represent the predicted signal sequence and the transmembrane (TM) regions detected by Phobius (https://www.ebi.ac.uk/Tools/pfa/phobius/). The pink shade indicates the predicted kinase domains based on Prosite (https://prosite.expasy.org). The locations of the kinase domain, ATP binding site and the active site are indicated according to Prosite. The *T. cruzi* recombinant kinase domain was produced according the indicated amino acid positions as KD4 and KD5. (B) Ribbon model of the kinase domain (KD4) of *T. cruzi* TcK2 (light blue) generated based on the mouse PERK structure (red, PDB id=3QD2.1.A) using SWISS-MODEL Homology Modeling (https://swissmodel.expasy.org) as detailed in **Supporting information file TcK2-KD4.pdf**. The arrows indicate the domains shown in (A) in addition to the putative P and activation loops. (C) and (D) Gel exclusion chromatography on Superdex-200 of the preparative purification of the kinase domain constructs KD4 and KD5. The insets show Coomassie blue staining of SDS-PAGE of the indicated fractions (1 mL each). (E) TcK2-KD5 mass spectrum, showing that it has the expected weight without no phosphorylation.

To identify possible TcK2 inhibitors and to generate active recombinant proteins, we started by analyzing the structure of its kinase domain in comparison with other trypanosome and eukaryotic homologues. We observed a high similarity among the PERK homologues of *T. cruzi*, *T. brucei*, and *Leishmania major* within the kinase domain (KD), including the predicted ATP-binding site and the active site (Fig. S1*A*). However, the predicted activation and P-loops of the *T. cruzi* enzyme, based on best fit modeling with the murine X-ray crystallographic PERK structure (Fig. S1*B*), are less conserved among the parasites. All three species possess a predicted signal sequence and transmembrane span, suggesting that the KD faces the cytosol. Furthermore, the N-terminal regions possess conserved features suggesting that they serve similar roles. Based on this analysis, the TcK2 gene of *T. cruzi* was used to construct clones for heterologous expression of the region coding the KD fused with a 6X-Histag at the N terminus. Two bacterial clones expressing the kinase domain of TcK2, KD4 ^(S659-Q981)^ and KD5 ^(S659-I976)^, were obtained, and large amounts of soluble recombinant proteins were purified by Ni^2+^-agarose and size exclusion chromatography, with a yield of ∼ 10 mg/L of purified protein per liter of bacterial culture (Fig. S1, *C* and *D*). Mass analysis indicates that KD5 has 38,810 kDa, the expected weight for the nonphosphorylated protein (Fig. S1*E*). We have not checked the final mass of KD4 as it showed more instability.

### Endogenous expression of the TcK2 kinase is observed in all parasitic stages

**Figure 1:**
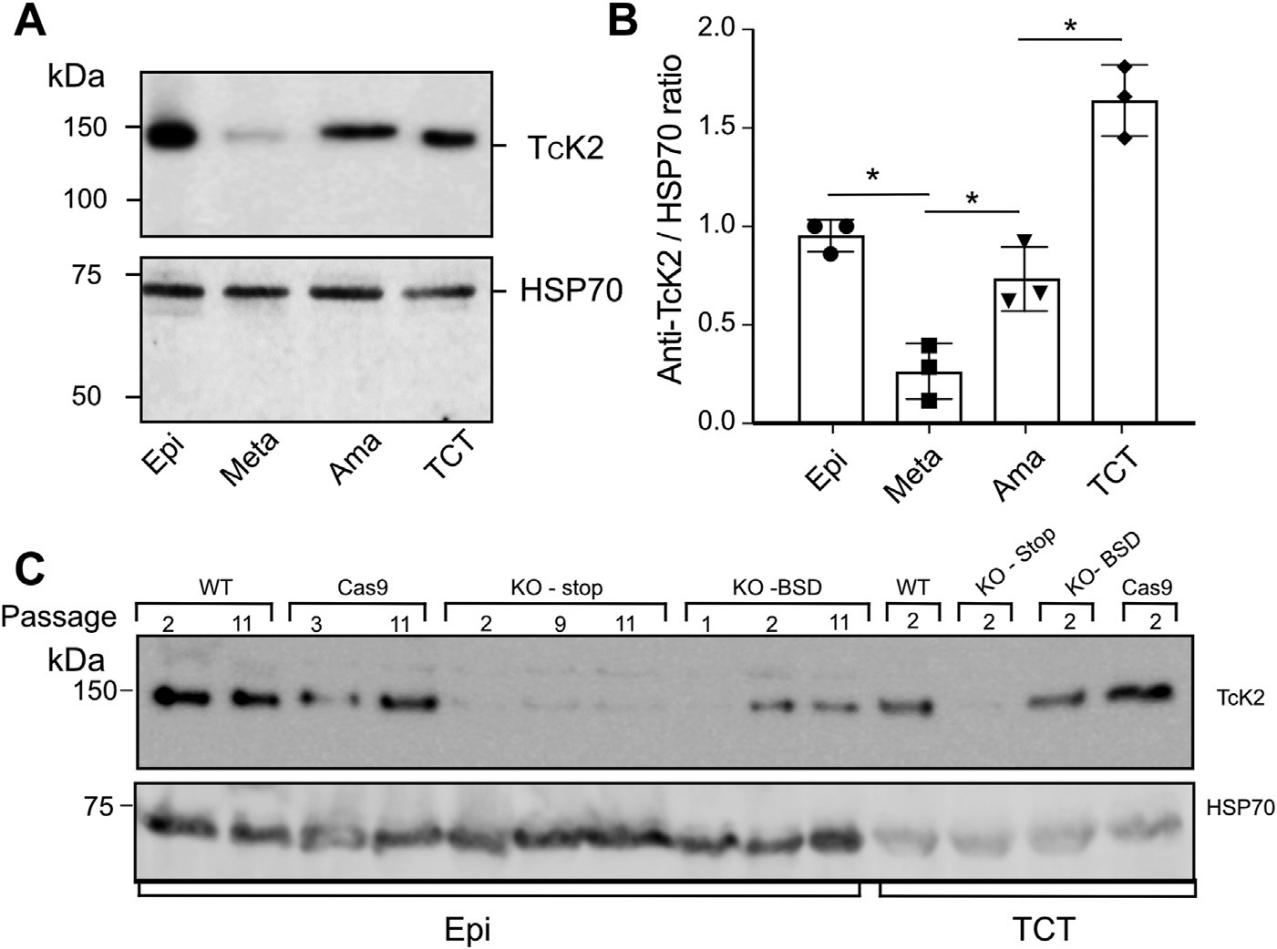
**TcK2 is expressed along *T. cruzi* stages.***A*, Western blot of samples containing the equivalent of 1 × 10^7^ parasites was probed with affinity-purified polyclonal rabbit antibodies to TcK2KD4 or HSP70. The analyzed stages were epimastigotes (Epi), metacyclic-trypomastigotes (Meta), intracellular amastigotes (Ama), and tissue culture–derived trypomastigotes (TCT). *B*, graph representing quantitative analysis of the relative band intensity (mean and SD) in different *T. cruzi* stages in three independent experiments. ∗, Significant differences using two-way ANOVA, with *p* < 0.05. *C*, Western blot using anti-TcK2 (*top*) or anti-HSP70 (*bottom*) of 1 × 10^7^ epimastigotes (Epi) or 1 × 10^7^ cell-derived trypomastigotes (TCT) at different passages, derived from original Y strain (WT), which was used to generate Cas9 expressing line, and the KO lines obtained with insertion of the stop codon or with the BSD insertion.

To evaluate the expression of TcK2 in the different parasitic stages, purified TcK2 KD4 was used to generate specific polyclonal antibodies after rabbit immunization and affinity chromatography with protein immobilized on CNBr-agarose. The antibodies recognized a band of the expected protein size in epimastigote extracts, which correspond to the *T. cruzi* form that develops in the insect vector midgut. TcK2 was also present in amastigotes, which proliferate in the cytosol of infected mammalian cells, and in trypomastigotes released from these cells (Fig. 1, *A* and *B*). We observed reduced expression in metacyclic-trypomastigotes (Metas) and increased expression in tissue culture–derived trypomastigotes (TCT) relative to proliferative epimastigotes and amastigotes.

### Insertion of stop codon in the gene of TcK2 generated the enzyme-depleted trypanosome cells

**Figure S2:**
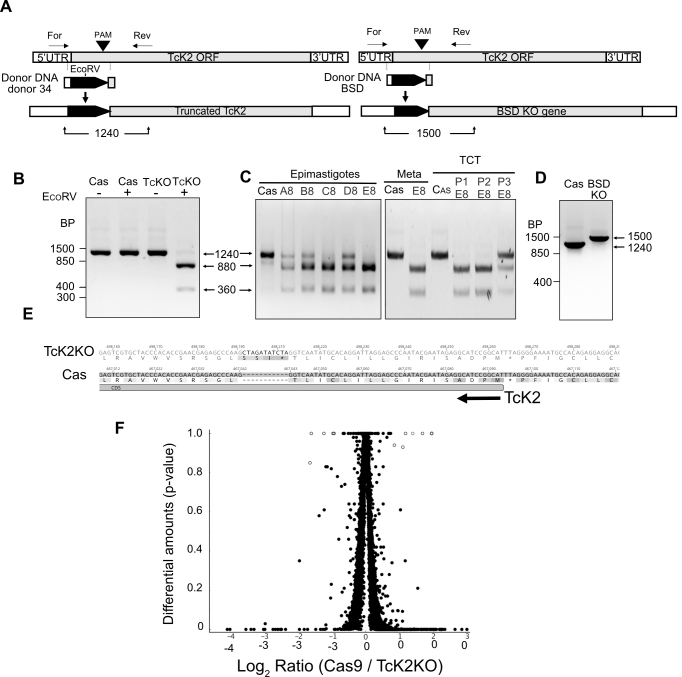
**Generation of TcK2 knockouts by CRISPR/Cas9 in *T. cruzi*.** (A) Diagram showing the two strategies employed to generate knockouts of TcK2. On the left, a donor with a stop codon containing the *EcoRV* site was used, while on the right a blasticidin (BSD) donor was employed. (B) Agarose gel electrophoresis of PCR products of the original line (Cas9) or the TcK2KO line using the indicated For and Rev primers of DNA extracted for a knockout clone that were treated (+) or untreated (-) with *EcoRV*. (C) Agarose gel electrophoresis of the PCR product of the Cas9 and TcK2KO isolated from metacyclic and TCT at the indicated infection cycles (P1 to P3). The first infection was made with metacyclic and the subsequent with the TCT released on day 7 post-infection. (D) Agarose gel electrophoresis of the PCR product of BSD resistant epimastigotes. (E) Comparison of the original sequence of the TcK2 ORF (Cas9) with the consensus alignment generated by Illumina sequence of the TcK2KO. The sequence follows the chromosome direction, and the gene is transcribed by the reverse strand as indicated by the arrow. (F) Comparative number of reads of the genome obtained by Illumina sequencing of the parental (Cas9) and the TcK2KO clone (KO-stop). The graphic shows the Log_2_ ratio of differential amounts of Illumina reads plotted against the p-value calculated by Student’s t-test using Geneious prime program. The positive numbers are increased hits and negative decreased hits of Cas9 compared to TcK2KO. The open dots correspond to major differences and the hits are detailed in the text and **Table S2**. **Table S2** also shows the SNP variations of Cas9 and TcK2KO clone.

We have previously obtained TcK2 null parasites by double replacement of TcK2 by antibiotic resistance genes (8) that were unable to grow efficiently as epimastigotes or amastigotes and did not survive after freezing and could not be recovered. Consequently, it was relevant to obtain new lines of TcK2 knockouts (TcK2KO) for this work, first to confirm the requirement of the kinase, then to understand why the enzyme was required for growth, and finally to test whether the parasite was more resistant to growth in the absence of TcK2 inhibitors in a drug developing program. Therefore, we generated new knockouts using CRISPR/Cas9 in epimastigotes for phenotypic characterization of TcK2 as a target for drug development. We used two different strategies: in one, we generated a donor DNA sequence to recombine near the Cas9 cleavage site and to insert a stop codon with an *EcoRV* site for diagnostic purposes as described (15) (Fig. S2*A*). After two rounds of transfection, a transfected population was subjected to PCR analysis detecting a region corresponding to the TcK2 sequence containing the *EcoRV* site. As some of the parental configuration remained, which could be due to incomplete digestion or contamination with wildtype cells (Fig. S2*B*), the population was cloned by limiting dilution. In several clones, the *EcoRV* undigested fragment was reduced, and it was barely detected when the DNA was isolated from the differentiated metacyclic form (Fig. S2*C*). However, after infection of mammalian cells, the arising TCTs showed a gradual increase of the wildtype phenotype. This observation also suggested that the WT gene was present and regained over the knockout after passages in TCT, which could be due to incomplete cloning or the presence of the original gene as an additional copy in the genome in some parasites. Therefore, for all experiments with TCT, we used the parasites appearing after an initial infection with metacyclic-trypomastigotes, which contained only the modified TcK2 gene, as checked routinely by PCR and *EcoRV* digestion and by Western blot.

The second strategy used the blasticidin resistance coding sequence (BSD) as donor. After drug selection, the isolated cell population presented a PCR product that was larger than the original gene (Fig. S2*D*). To further confirm that these insertions affected mainly the TcK2 gene, a library of total DNA of both the Cas9 original strain and the TcK2 generated by insertion of the stop codon was used to generate a sequencing NGS library. The sequencing analysis indicated that more than 99% of the sequences contained the insertion at the expected position with the generation of the stop codon and restriction site (Fig. S2*E*). Comparative analysis of the hit levels of both coding sequences was aligned with the YC6 genome of *T. cruzi* (16). Illumina sequence showed that one hit with the WT sequence with unchanged bases was detected in 100 sequences encompassing the changed region in the library, suggesting that the wildtype gene might be present in the TcK2KO clone. Moreover, it showed few genomic modifications (Fig. S2*F*). We found depletion of TcYC6_0108810, a hypothetical protein, as it was only detected in the parental line. In contrast, TcYC6_0077900, also a hypothetical protein, was only seen in the TcK2KO. Statistically significant differences are highlighted with open symbols in Fig. S2*F*. From these, five are changes in genes coding for pseudogene transcripts. The coding sequences with decreased hits in the TcK2KO were TcYC6_0044920, a putative SNF2 DNA repair protein; TcYC6_0108810, a hypothetical protein; TcYC6_0064880 and TcYC6_0047600, both hypothetical proteins found only in *T. cruzi*. Only copies of two coding genes were found significantly increased in the TcK2KO: TcYC6_0063930, a viral-type inclusion protein, and TcYC6_0077450, coding for a unique hypothetical protein in the YC6 strain (see full list in Table S1, which also includes the observed single nucleotide polymorphism). Such variations have been frequently observed in sequenced *T. cruzi* genome (17, 18).

Next, we evaluated the expression of TcK2 mutants by performing Western blot analysis. The TcK2 protein was not detected in the KO generated by the stop codon insertion after several passages of epimastigotes, in contrast to the wildtype Y strain and the parental line containing the Cas9 gene (Fig. 1*C*). However, the line generated by BSD insertion even after cloning was able to recover TcK2 expression, despite the presence of antibiotic selection in epimastigotes.

### TcK2-depleted cells have a lower rate of proliferation

**Figure 2:**
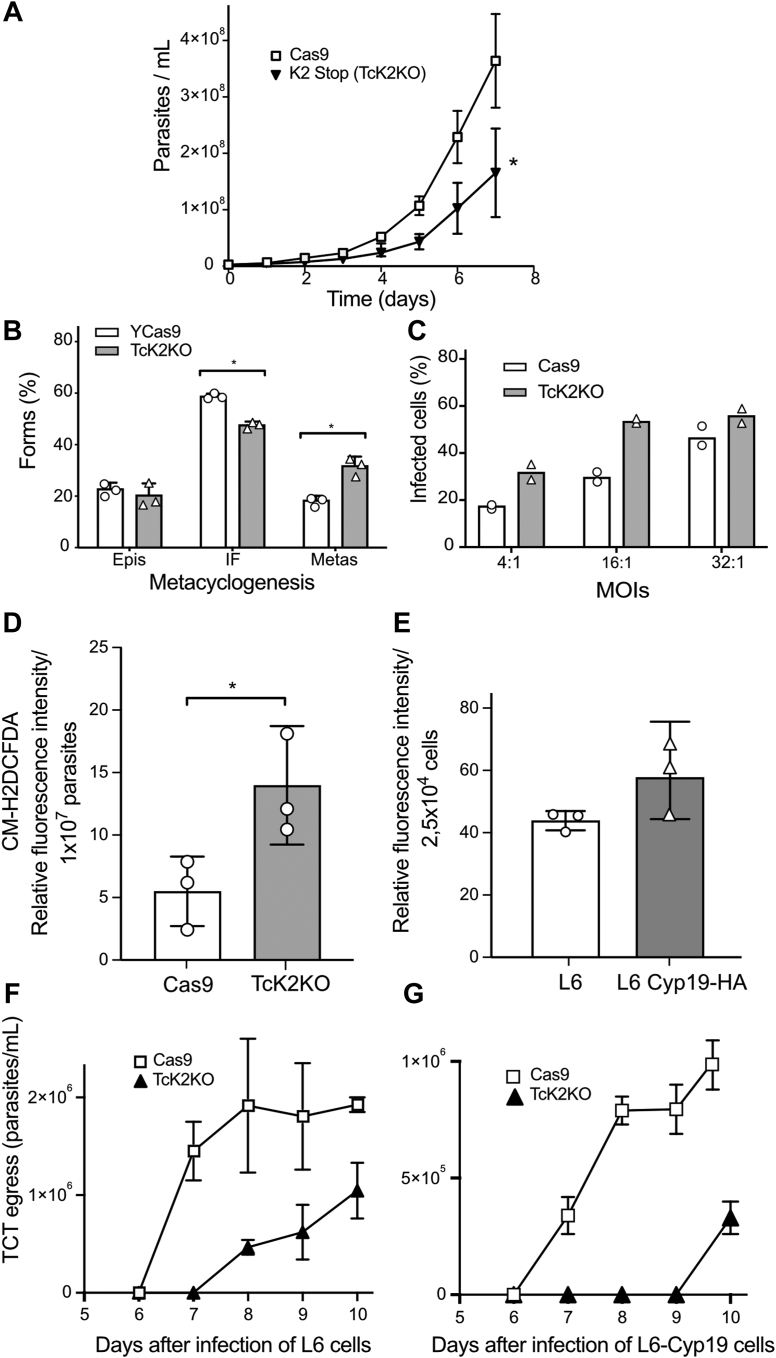
**Epimastigote and amastigote proliferation is inhibited by TcK2 depletion, while differentiation in metacyclics and trypomastigote invasion are preserved**. *A*, proliferation of the parental (Cas9) and TcK2-depleted epimastigote parasites in normal medium. The values are means of triplicate experiments. *B*, late exponential epimastigote cultures (2 × 10^7^/ml) were washed and incubated in Grace’s medium. After 7 days, the total number of Epis, Intermediate Forms (IF), and Metas were estimated by Giemsa staining of 250 parasites in three independent experiments. *C*, L6 rat myoblasts (1 × 10^5^) were plated on round coverslips in 24-well plates. After 24 h, the cells were incubated with 0.5 ml purified metacyclics at the indicated multiplicity of infection (MOIs) for 3 h at 37 °C, and the number of internalized parasites per cell was counted by Giemsa staining as described in Methods. *D*, ROS levels measured with CM-H2DCFDA of epimastigotes and (*E*) of L6 and L6 expressing *T. cruzi* cyclophilin 19. The values are triplicate experiments and with *p* = 0.05 and 0.14, respectively, calculated by two-tailed unpaired *t* test. *F* and *G*, egress of TCT from L6 myoblast, and L6 myoblast overexpressing *T. cruzi* cyclophilin 19, respectively, infected for 3 h to obtain the same infectivity with Cas9 and TcK2KO parasites TCTs. The graphs show the daily number of TCTs collected in the culture supernatant. All the values are means and SD (n = 3), and asterisks indicate *p* < 0.05 using two-way ANOVA.

To evaluate the consequences of TcK2 depletion, we first evaluated the growth and differentiation of the epimastigote form into infective metacyclic-trypomastigotes. The TcK2KO clone with the stop codon insertion showed a decreased multiplication rate compared with the parental epimastigotes (Cas9) (Fig. 2*A*), consistent with our earlier work using double replacement of TcK2 by antibiotic resistance genes (8). However, the effects were less intense in the present case, which might be due to the way the knockouts were generated. On the double replacement the selection was made in the presence of antibiotics, while in the present study, a stop codon was inserted without drug selection, but the resulting cell expressed Cas9 protein, which in either case could produce combined effects. The epimastigotes were used to generate metacyclic-trypomastigotes after incubation in Grace’s medium. A quantitative analysis by counting microscopically the number of parasites with different shapes, as shown in reference (19) indicated that the TcK2KO line was more effective in generating metacyclic forms (Fig. 2*B*), which were also more infective than the parental Cas9 line (Fig. 2*C*), similarly to what was obtained before (20).

We have previously suggested that the less efficient growth and increased differentiation was due to the presence of higher levels of ROS (20). Indeed, ROS levels were also increased in the presently generated TcK2KO epimastigotes (Fig. 2*D*). Because the oxidative levels were found to impact the intracellular growth, we assessed whether the growth and the subsequent release of trypomastigotes were affected in cells containing distinct ROS levels. For this, we compared the growth in L6 rat myoblasts with L6 cells expressing the *T. cruzi* cyclophilin 19, which is secreted by the parasite and found to increase the intracellular ROS (Fig. 2*E*) (20). TCT knockout, although invading more efficiently both cells, showed limited intracellular proliferation, producing less TCTs in both cases (Fig. 2, *F* and *G*). However, the later TcK2KO egress from infected cells was more pronounced in L6 cells expressing cyclophilin19 (Fig. 2*G*), suggesting these differences might be related to the management of ROS, which would be less efficient in the absence of TcK2.

### Increased expression of trypomastigote proteins In TcK2 knockout cells

**Figure 3:**
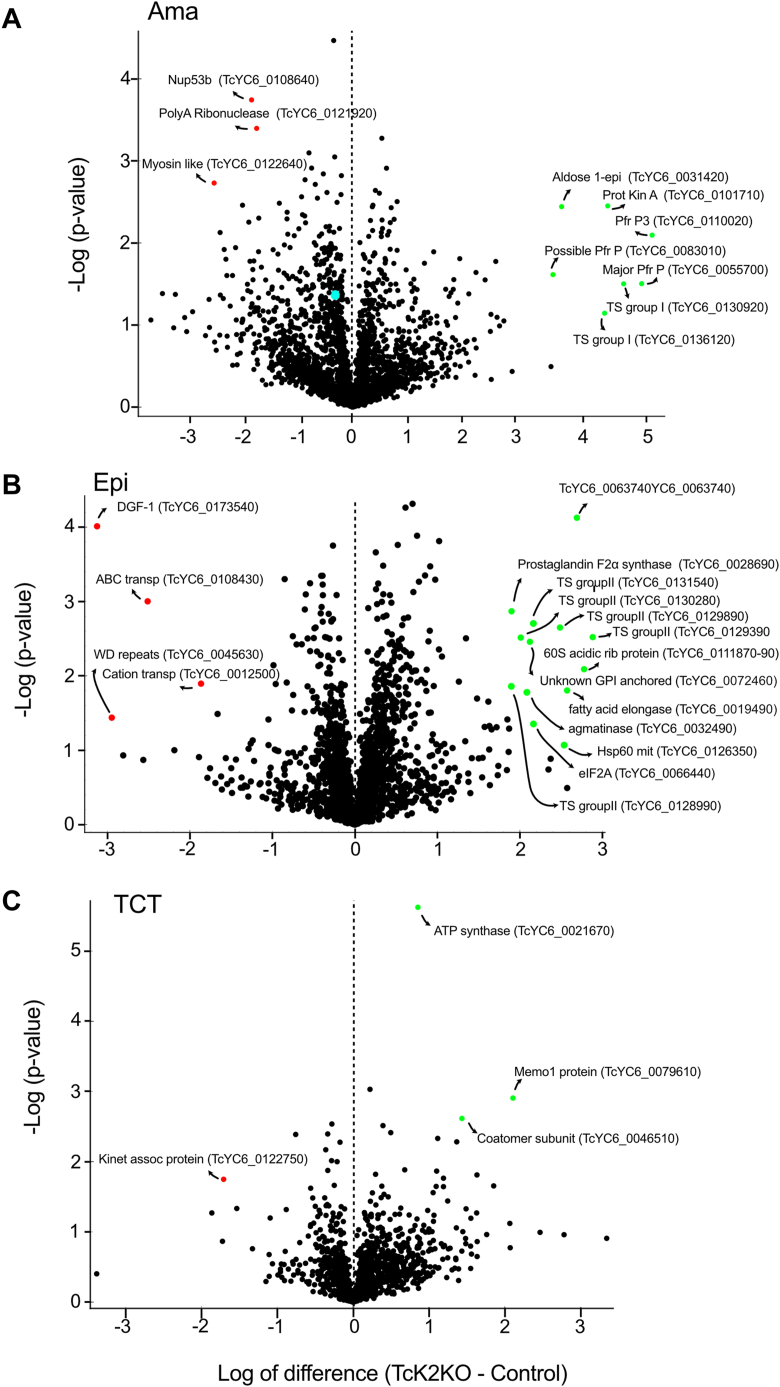
**Global proteome changes upon TcK2 knockout for three *T. cruzi* life-cycle stages**. Volcano plots of −log_10_*t* test *p*-value plotted *versus* the *t* test difference (difference between means) comparing protein abundance of TcK2KO cells with the respective parental cells—for (*A*) intracellular amastigotes collected 72 h after infection (Amas), (*B*) exponentially growing epimastigotes (Epis), and (*C*) TCTs released from infected L6 cells. The complete results from label-free quantification are in Table S3. The green symbols show increased and red decreased levels in TcK2KO relative to the parental line (Cas9). The annotated names are show with the respective accession number in https://tritrypdb.org. The blue dot indicates eIF3a.

**Figure 4:**
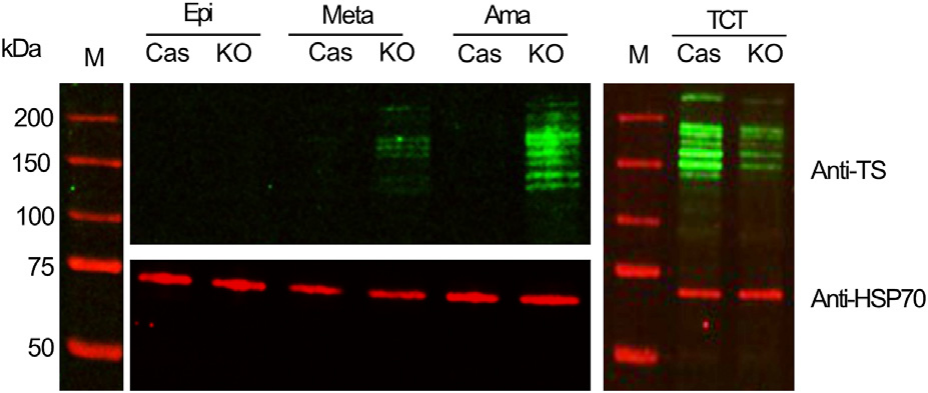
**Trans-sialidase expression is increased in intracellular amastigotes of TcK2KO.** Western blot of the Cas9 and TcK2KO of epimastigote (Epi), metacyclic-trypomastigotes (Meta), intracellular amastigotes (Ama), and tissue culture–derived trypomastigotes (TCT) extracts, each containing 20 μg of protein, probed with trans-sialidase monoclonal antibody (mAb 39, *green*) and anti HSP70. Lane M indicates the molecular weight markers of each gel.

To gain more insight about the reduced proliferation caused by TcK2 depletion, cell extracts of amastigotes, epimastigotes, and TCTs were analyzed by label-free liquid chromatography–tandem mass spectrometry. As shown in Figure 3, *A*–*C*, the differences in protein expression suggested that TcK2KO proliferating forms have increased levels of some trypomastigote-specific proteins. For example, in the epimastigotes and intracellular amastigotes of the knockout we observed an increase in members of the trans-sialidase family genes, and paraflagellar rod proteins 1 and 2, all typical of trypomastigote stages (Fig. 3 and Table S2). In epimastigotes, we observed an increased expression of ribonuclease mar 1 (TcYC6_0063740), 60S acid ribosomal protein (TcYC6_0111890), fatty acid elongase (TcYC6_0019490), and prostaglandin synthetase (TcYC6_0028690), all enriched in the parasites after differentiation into metacyclic-trypomastigotes (21). In contrast, an ABC transporter (TCYC6_0108430) more expressed in epimastigotes was found decreased in epimastigotes. Similarly, in amastigotes there was a small decrease of amastin (TcYC6_0087860 and TcYC6_0087830) as well as myosin-like coiled-coil protein (TcYC6_0122640), glycerate kinase (TcYC6_0044810), and nucleoporin 53b (TcYC6_0108640) (Table S2), which are key proteins for amastigote growth. In TCT, the main changes in TcK2KO are a decrease in a kinetoplast associated protein 4 (TcYC6_0122750) and an increase in a subunit of ATP synthase and a Memo-1 protein (TcYC6_0079610), involved in microtubule recognition of extracellular signals (Table S2). To confirm these observations, we probed extracts of parasites of these different stages with antibodies against trans-sialidase. We verified that the trans-sialidases belonging to group I, characterized by showing enzymatic trans-glycosidase activity and containing the typical 12 amino acid repeats (22, 23), are highly expressed in intracellular TcK2KO amastigotes as compared with parental amastigotes (Fig. 4). Significantly, expression of this protein family was not observed in epimastigotes, but by contrast epimastigotes appear to be enriched in sialidase-like proteins of group II.

### Phosphoproteomics indicates eIF3a and kinesins as potential substrates for TcK2 in amastigotes and CAP methyltransferase and cAMP response protein in epimastigotes

**Figure 5:**
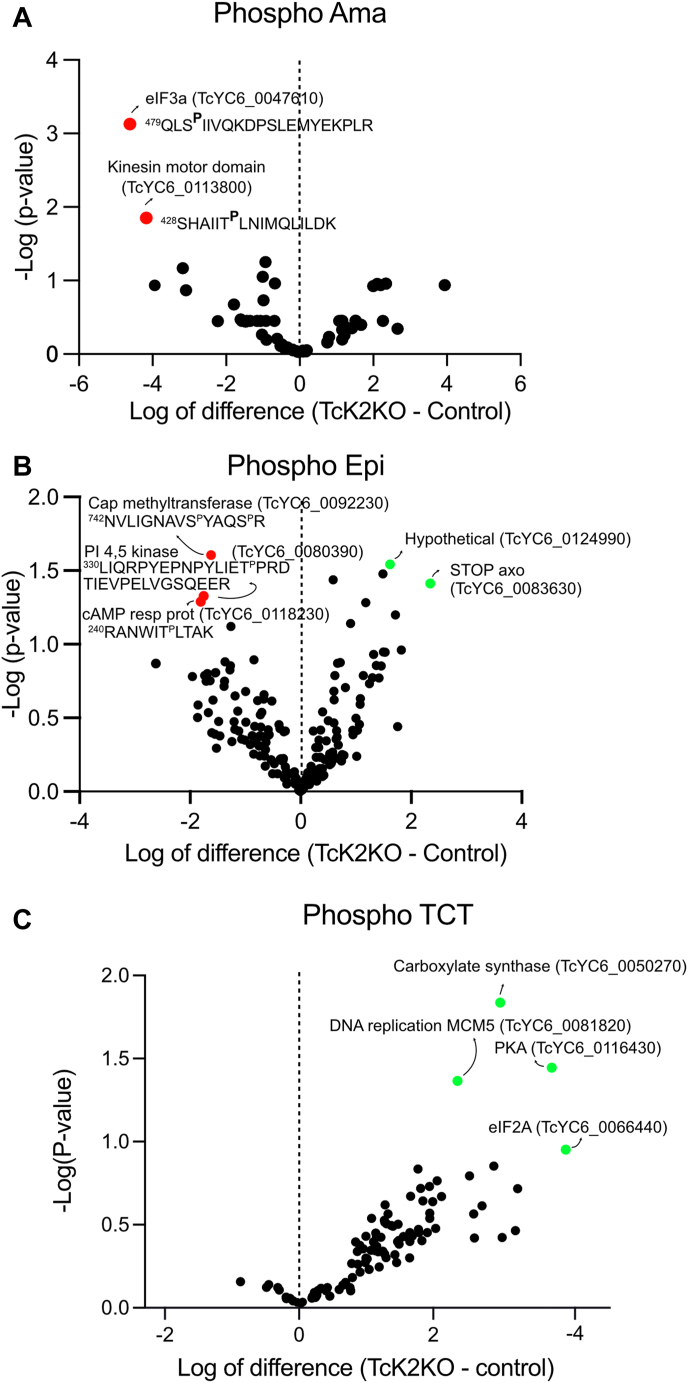
**Major protein phosphorylation changes between parental TcK2KO lines of *T. cruzi* stages**. Volcano plots of −log_10_*t* test *p*-value plotted *versus* the *t* test difference (difference between means) comparing phosphoprotein abundance of TcK2KO cells with the respective parental cells for (*A*) intracellular amastigotes collected 72 h after infection (Amas), (*B*) exponentially growing epimastigotes (Epis), and (*C*) trypomastigotes released from infected L6 cells (see also Table S3). *Green* symbols are proteins increased and *red* decreased in the TcK2KO relative to the control samples. The corresponding proteins with the respective accession number in https://tritrypdb.org and with the respective peptides and phosphorylation sites are shown in the figure.

We were also able to obtain quantitative data for the protein phosphorylation status of TcK2KO in comparison with the parental line (Fig. 5, *A*–*C* and Table S3). In amastigotes, we found eIF3a and a kinesin motor domain protein phosphorylated only in the parental line, suggesting they could be substrates for TcK2. The eukaryotic initiation factor 3a (eIF3a, the largest subunit of eIF3), is part of the 43S preinitiation complex formed by the small ribosomal subunit (40S) bound by initiation factors eIF1, eIF1A, eIF3 and the eIF2, GTP, and activated ^met^tRNA ternary complex (24). The kinesin motor domain protein, also annotated as flagellum attachment zone protein 7, is associated with the flagellum attachment to the cell body (25). The phosphorylated sites for these proteins are depicted in Figure 5*A*. In epimastigotes, three other proteins were less phosphorylated in the TcK2-depleted clone. These are CAP methyltransferase, a phosphatidyl inositol 4,5 kinase, and a protein annotated as cyclic AMP responsive protein 4. Interestingly, TCTs showed an increased protein phosphorylation of some proteins in the depleted clone. It is particularly interesting to find increased phosphorylation in eIF2A, a protein that is believed to replace the entire three-subunit eIF2 in translation initiation (26), as only eIF2α is normally a substrate for PERK when ER stress arises (3). This finding could explain alternative translation initiation mechanisms in trypanosomes at the different parasitic stages (27).

### TcK2 phosphorylates *T. cruzi* eIF2α under starvation stress and can phosphorylate mammalian eIF2α *in vitro*

**Figure 6:**
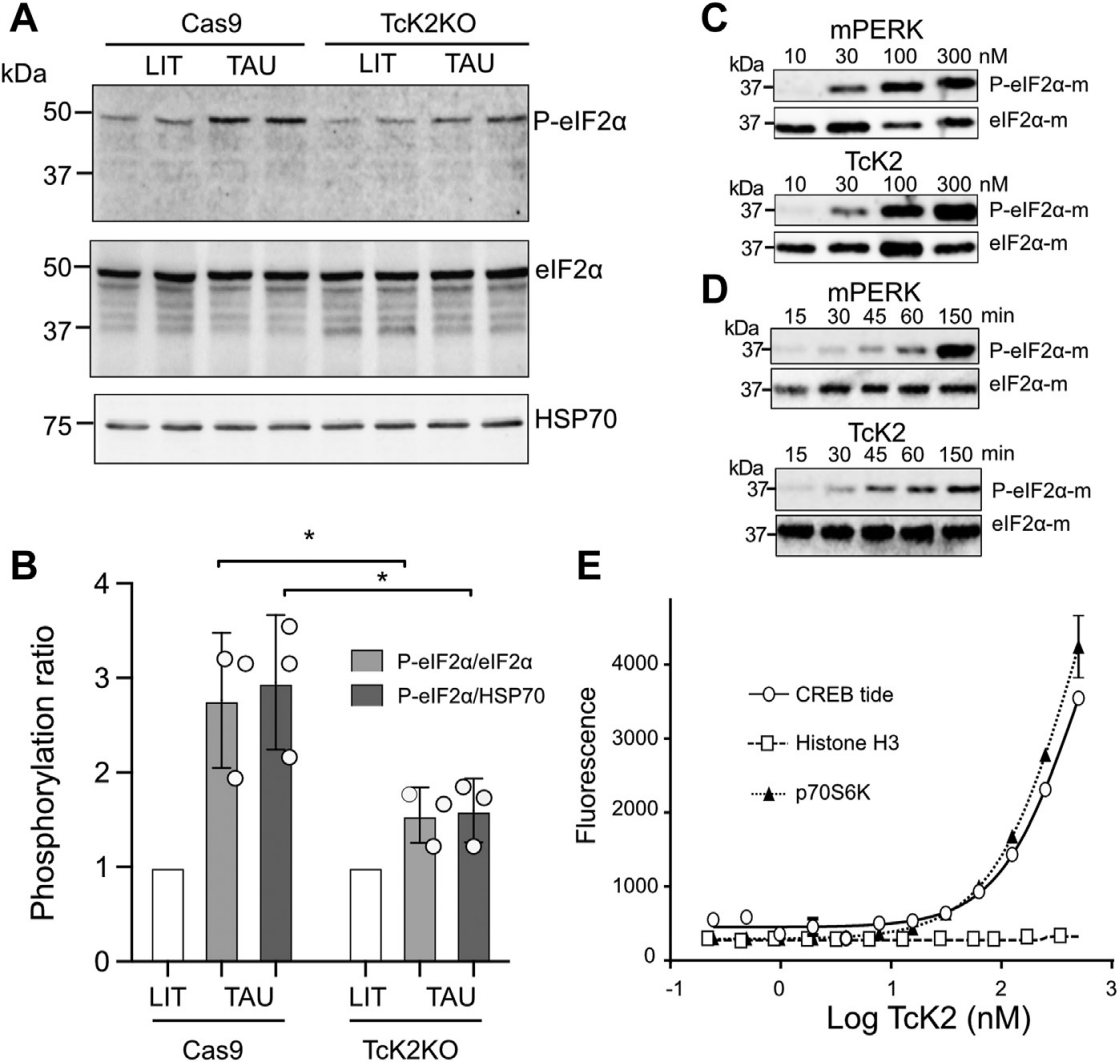
**TcK2 specificity *in vivo* and *in vitro***. Western blot of extracts containing 1 × 10^7^ parasites of parental (Cas9) and TcK2KO epimastigotes incubated in LIT or TAU solution for 2 h, at 28 °C and probed with rabbit anti-phospho-eIF2α (*top panel*), total mouse anti-eIF2α (*middle panel*), and rabbit anti-eIF2α developed with the respective IRDye anti-IgG conjugates and anti-HSP70. *B*, relative phosphorylation of triplicate experiments for the parental and TcK2KO lines incubated in TAU as in (*A*) compared with parasites maintained in LIT. The ratio was measured relative to total eIF2α or HSP70 as indicated. The *asterisks* indicate *p* < 0.05 calculated by two-way ANOVA. *C*, Western blots of samples containing the indicated concentrations of recombinant mouse PERK kinase domain or TcK2 (KD4) for 1 h, at 28 °C in the presence of 10 μM recombinant mouse eIF2α and 0.2 mM ATP. The blots were decorated with anti-phospho-Ser51 antibodies and anti-eIF2α. *D*, Western blots of samples of recombinant mouse eIF2α (10 μM) incubated with ATP (0.1 mM) and 100 nM of recombinant PERK or TcK2 (KD4) for the indicated times and decorated as above. *E*, effect of TcK2 concentrations on the phosphorylation of synthetic peptides used as substrate: CREBtide, histone H3, and p70S6K, by employing the LANCE assay after 3-h incubation.

We asked whether the TcK2-depleted parasites could lack eIF2α phosphorylation as we detected several peptides of eIF2α protein including the one that is proposed to be phosphorylated at Thr169 (Table S3), but we could not find changes in the eIF2α phosphorylation in the proteomics of any the analyzed stages of *T. cruzi.* This might be due to either that eIF2α is poorly phosphorylated or that the phosphorylated site is close to several Arg, known to prevent trypsin digestion (28). In fact, phosphoproteome studies in trypanosomes did not detect this modification in eIF2α (29, 30, 31). Therefore, we tested whether eIF2α phosphorylation was present in the TcK2-depleted cells by Western blot upon nutritional stress in saline supplemented with 2 mM Ca^2+^ and Mg^2+^ (TAU solution). As previously shown (10), we found increased phosphorylation upon TAU incubation in parental lines but significantly reduced phosphorylation in the TcK2-depleted line (Fig. 6, *A* and *B*). This result suggested that TcK2 is indeed involved in eIF2α phosphorylation in the parasite, which could not be detected by proteomic analysis.

**Figure S3:**
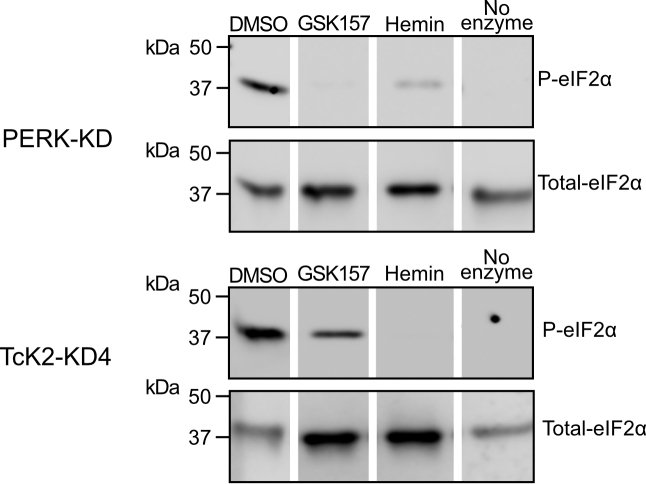
Effects of hemin and GSK2656157 in the mammalian eIF2α phosphorylation by PERK and TcK2-KD4. Western blots of samples of recombinant mouse eIF2α (10 μM) incubated 1 h at 28°C with ATP (0.1 mM) and 100 nM of recombinant PERK or TcK2 (KD4) pre-incubated 30 min with the indicated inhibitors at 10 μM. Reactions were detected with anti-phospho-Ser51 antibodies to eIF2α and with total anti-eIF2α, respectively detected with anti-rabbit IgG IRDye800 and anti-mouse IgG peroxidase.

We also incubated the purified recombinant TcK2 KD4 with mouse-eIF2α in the presence of ATP. TcK2 was able to phosphorylate mouse eIF2α in a concentration- and time-dependent manner (Fig. 6, *C* and *D*). To complement this observation, we used an *in vitro* phosphorylation assay that detects specific phosphorylation. As shown in Figure 6*E*, TcK2 KD4 phosphorylated the peptides corresponding to cAMP response element-binding protein (CREB) and the ribosomal protein S6 kinase beta-1 kinase (p70S6K) but not peptides corresponding to histone H3. The CREB peptide, CKRREI**L**SRRPS**Y**RK, can be phosphorylated in both Ser, p70S6K in the Thr of the LG**F**T**Y**VAP motif, and histone H3 in the 3rd Thr and 10th Ser of the peptide ARTKQTARKSTGGK. The common feature of CREB and p70S6K is the presence of at least one hydrophobic residue (in bold) next to the phosphorylation site, absent in the histone H3 peptide. Furthermore, this matches the characteristics of the eIF2α phosphorylation site and was also observed in all the proteins with decreased phosphorylation by the proteomic analysis, except for PI 4,5 kinase (Fig. 5). We additionally explored the capacity of the PERK strongest inhibitor to prevent mammalian eIF2α phosphorylation by TcK2-KD4. At 0.1 μM it fully prevented phosphorylation of eIF2α by PERK, but even at 10 μM no decrease in the phosphorylation was observed for the *T. cruzi* enzyme (Fig. S3). Interestingly, we confirmed the inhibitory effect of hemin as shown before (8), which also acted on PERK activity *in vitro*.

### A compound screen identifies TcK2 inhibitors

**Figure 7:**
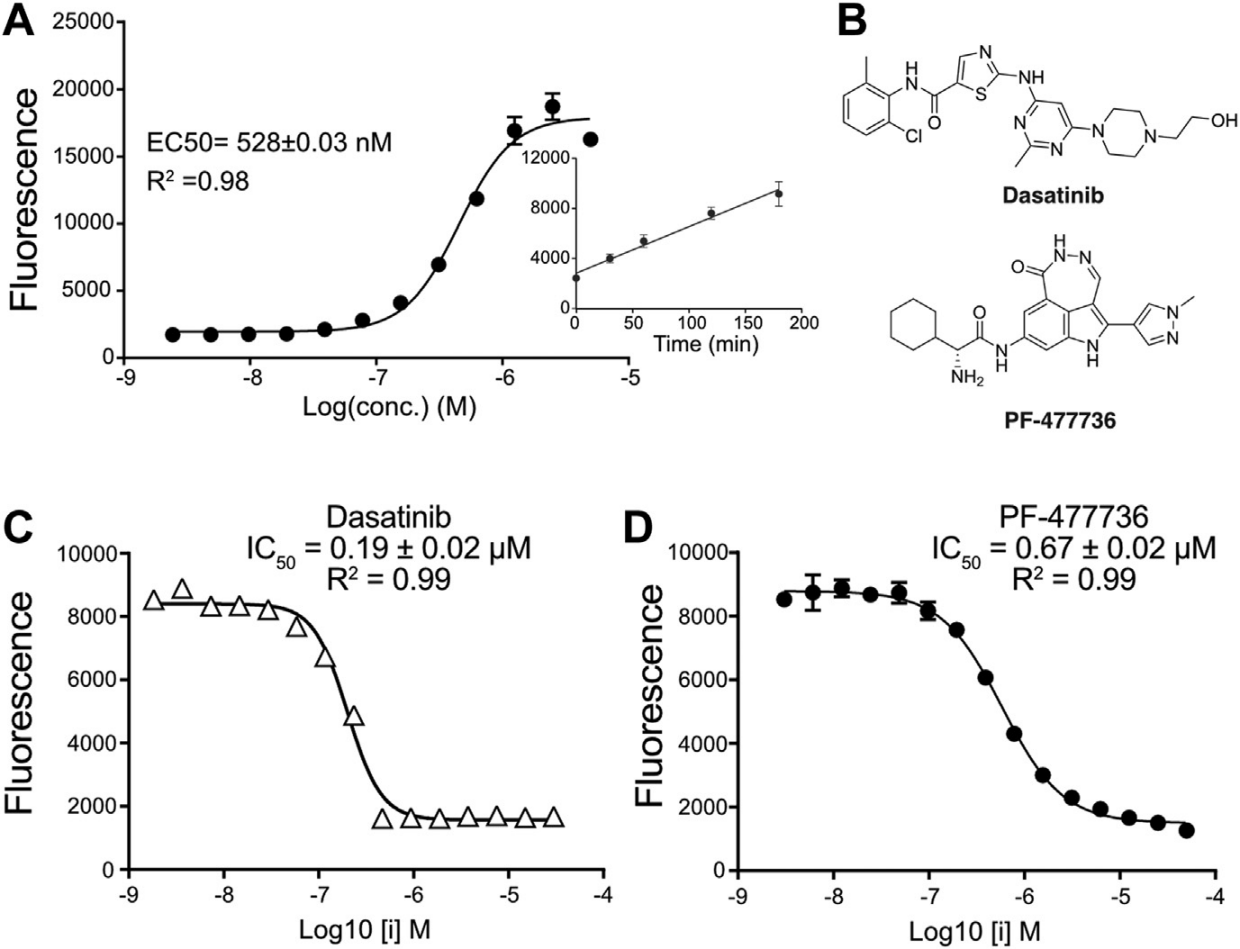
**Kinetic analysis of TcK2 phosphorylation activity**. *A*, recombinant TcK2 (KD5) at the indicated concentrations in duplicates was incubated with 0.2 mM ATP, 50 nM Ulight p70S6K substrate in kinase buffer for 3 h at 23 °C in 10 μl. Then, 6 mM EDTA was added, and the mixture was incubated for 1 h with the Europium-antibody. The substrate phosphorylation was estimated by the fluorescence energy transfer, and the corrected emission reads at 615 nm are shown. The panel shows the calculated EC_50_ and the R^2^ for the enzyme concentration. The inset shows the time course of the reaction using 430 nM of enzyme. TcK2 KD5 (430 nM) was incubated in duplicates. *B*, structures of Dasatinib and PF-477736. *C*, phosphorylation promoted by TcK2 KD5 (400 nM) in p70S6K incubated for 3 h, at 23 °C with the indicated concentrations of Dasatinib (*triangles*) or (*D*) PF-477736 (*circles*) in triplicates. The IC_50_ for Dasatinib was 0.199 ± 0.02 μM (R^2^ = 0.99) and 0.67 ± 0.02 μM (R^2^ = 0.99) for PF477736, calculated using GraphPad Prism software. In all panels, the values are given as means and standard deviation.

Because the available mammalian PERK inhibitor GSK2656157 had poor capacity to inhibit the *T. cruzi* enzyme and the finding that TcK2 KD5 is more stable than TcK2 KD4, the KD5 form of the enzyme was used to screen a library of 379 kinase inhibitors from Selleckchem by differential scanning fluorimetry (Table S4). Sixty-three compounds were identified that increased the melting temperature (ΔTm) of the enzyme by more than 1 °C. Among the five ligands with ΔTm > 5.5 °C, only Dasatinib and compound PF-477736 inhibited the enzyme (Table 1) in the chosen follow-up experiment, a quantitative enzymatic assay (LANCE). The latter was developed as a 384-well plate format using the TcK2 KD5 construct. The enzyme was fully characterized with phosphorylation activity observed in a linear range between 10 and 1 μM of enzyme (Fig. 7*A*) and linear over time up to 200 min with ATP concentrations up to 100 μM (Fig. 7*A*, inset). The Km for ATP was determined as 1.72 μM. At optimized conditions we observed that Dasatinib and PF-477736 (Fig. 7*B*) inhibit TcK2 with IC_50_ = 0.19 ± 0.02 μM and 0.67 ± 0.02 μM, respectively (Fig. 7, *C* and *D*). Dasatinib is a protein tyrosine kinase inhibitor that targets Src/Abl kinases in leukemia, and PF-477736 is a serine–threonine Chk1 kinase inhibitor.

**Table 1:** Compounds that bind TcK2 and inhibit enzymatic activity

Ligand	ΔTm (°C)	Activity inhibition
GZD824	15.7	No inhibition
**Dasatinib**	9.1	Inhibition
PLX-4720	8.9	No inhibition
**PF-477736**	6.1	Inhibition
Dabrafenib (GSK2118436)	5.8	No inhibition
Ponatinib (AP24534)	5.3	NT^a^
Bosutinib (SKI-606)	5.1	NT^a^
Saracatinib (AZD0530)	4.3	NT^a^
Vandetanib (ZD6474)	4.2	NT^a^
Golvatinib (E7050)	3.8	NT^a^
DMSO	0.2	No inhibition

a Not tested. Compouns marked in Bold were found to inhibit TcK2.

### Dasatinib inhibits intracellular *T. cruzi* proliferation in parental but not in the TcK2 knockout

**Figure 8:**
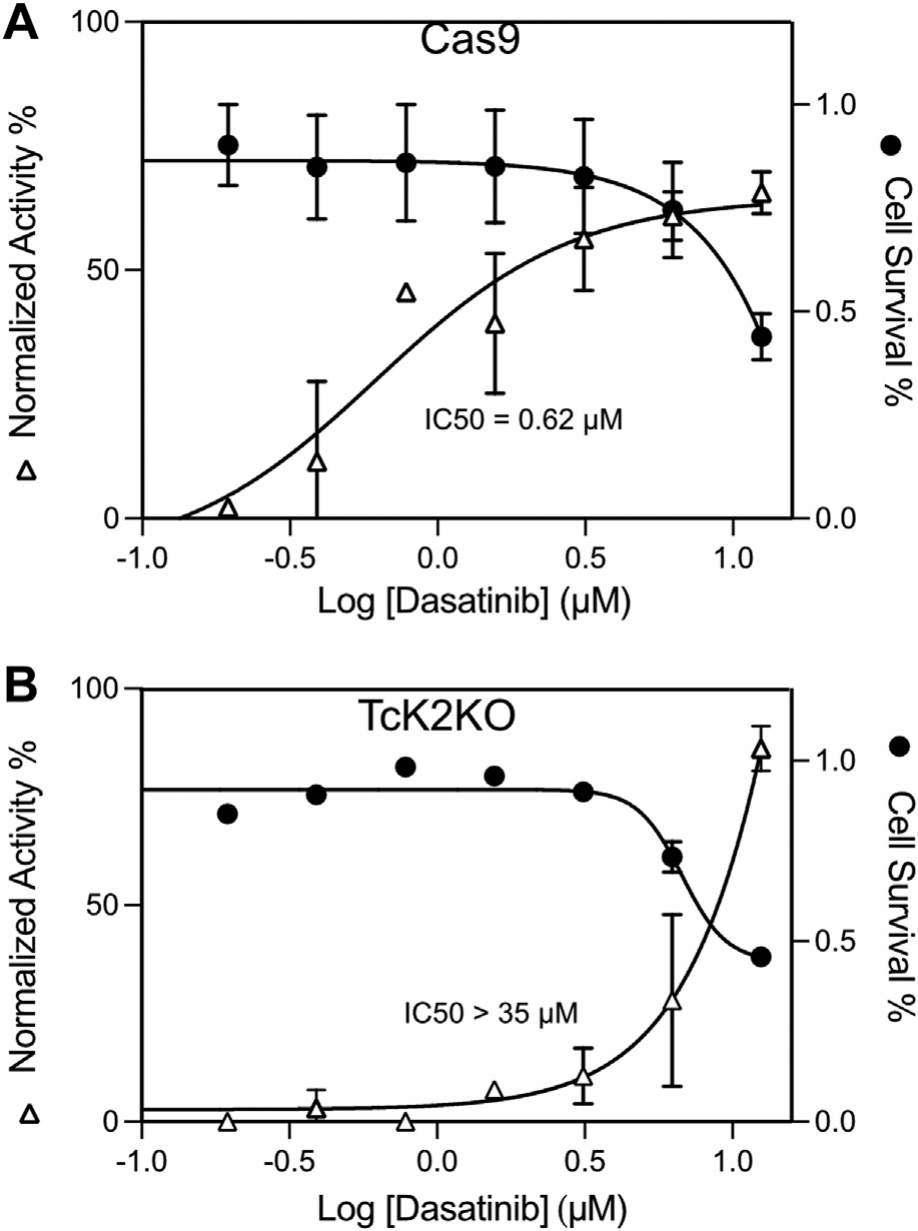
**TcK2KO proliferating amastigotes are more resistant to Dasatinib than wildtype parasites**. L6 rat myoblasts (1 × 10^3^) were seeded in black 96-well plates with transparent bottom in 120 μl of DMEM medium containing 10% FBS. After 24 h, 30 μl of TCT of the Cas9 *(A)* or TcK2KO line (first passage, *B*) was added (MOI=20) and the cells were maintained at 37 °C. After the next 24 h, serial dilutions of Dasatinib were added in a volume of 30 μl and the incubation was continued for 72 h, before fixing the cells with 4% *p*-formaldehyde in PBS, washing once with PBS and staining with Draq5. Imaging was carried out using an automated cell plate reader. Infection ratio (IR) was defined as the ratio between (i) the total number of infected cells in all images from the well and (ii) the total number of cells in all images from the same well. The values are means and standard deviations from duplicate experiments, each one made in two wells for each concentration.

As Dasatinib was more potent in inhibiting TcK2, we tested its effect on mammalian cells using a High Content Screening platform. Moreover, PF-477736 was toxic for L6 cells and hence activity against the parasite could not be evaluated. Dasatinib inhibited parasite growth in L6 cells with an IC_50_ of 0.6 ± 0.2 μM, and cytotoxicity toward L6 cells was above 5 μM (Fig. 8*A*), similar to the Dasatinib IC_50_ of 4 μM determined in HepG2 cell (32), suggesting a selectivity window of approximately 8-fold. Foremost, the activity of Dasatinib against the growth of TcK2-depleted parasites was above 35 μM (Fig. 8*B*). These results indicated that the absence of TcK2 rendered the parasite >20 times less sensitive and are strong evidence that TcK2 is the major Dasatinib target during parasite proliferation inside mammalian cells.

## Discussion

Identification of relevant drug targets and respective lead compounds is challenging (33, 34). When considering parasitic protozoans, this is particularly difficult as they have distinct enzymatic and metabolic pathways (12, 13). We previously reported that protein kinase TcK2 knockouts display attenuated intracellular proliferation due mainly to the incapacity to deal with ROS (8). Here we confirm this finding using a complementary strategy and further understanding of TcK2 function in promoting proliferation. To identify inhibitors and candidate drugs for therapy, we expressed and purified recombinant TcK2 kinase domain and developed an assay to screen for inhibitory compounds. We identified candidate compounds by differential scanning fluorimetry and confirmed submicromolar range inhibition for the kinase inhibitors Dasatinib and PF-477736. Furthermore, Dasatinib blocks parasite proliferation only in cells expressing TcK2, validating that it is the target for this compound.

Regions selected for expression of recombinant TcK2 kinase were based on structural predictions using Swiss Modeler and sequence comparisons with orthologous enzymes in other Trypanosomatids. The N- and C-terminal regions of the kinase could be assigned with high confidence and displayed high similarity with the PERK structure among several other protein kinase structures (Table S2 in supporting information file TcK2-Kd5.pdf). The TcK2 model also predicts a nonstructured domain in the P-loop region, smaller than the one found in metazoan PERK and likely related to substrate binding (35). In addition, the model suggests an unstructured activation domain that is variable in sequence between trypanosomatid species, suggesting potential substrate divergence and/or activation mechanisms within the lineage.

Purified recombinant TcK2 was obtained in high yields and was in the dephosphorylated state as observed by mass analysis. This was expected as the protein was produced in *Escherichia coli* expressing lambda phosphatase (36). Both recombinant TcK2 protein constructs showed protein kinase activity and were able to phosphorylate recombinant mouse eIF2α at Ser52, as well as CREBTide and p70S6K peptides. The recombinant kinase domain of TcK2 was also used to generate specific antibodies *via* affinity purification. These antibodies recognized TcK2 in proliferating forms of the parasite (epimastigotes and amastigotes) and in trypomastigotes released from infected cells. The relative enzyme levels were largely decreased in metacyclic-trypomastigotes, which agrees with the observations that metacyclic-trypomastigote have low levels of translation (37) and contain diminished amounts of eIF2α (11). TcK2-specific antibodies also helped to confirm the complete elimination of the protein in the parasites subjected to CRISPR/Cas9 gene knockout.

Stop codon insertion without drug selection abrogated TcK2 expression without regain of the kinase for several divisions in epimastigotes and upon differentiation in metacyclic-trypomastigotes. In addition, sequence analysis indicated that most of the parasite population contained the stop codon in the TcK2 open reading frame. The TcK2KO lines presented a decreased growth rate, differently from the lines in which the entire gene was replaced by BSD as selective drug, which recombined and reacquired the original TcK2 gene after a few divisions as epimastigotes. The insertion of stop codons has been successfully used in *T. cruzi* in the absence of antibiotic selection (15). As shown previously, TcK2 null parasites obtained by gene replacement (8) showed increased ROS levels, but with a less strong phenotype, probably due to the presence of drugs and expression of antibiotic resistance markers. Moreover, sequence analysis of the knockout clone indicated a few changes in the genome. Only one hypothetical protein was completely lost in the modified strain, and two appeared in the TcK2 null strain. The number of copies was also increased for two coding genes (one hypothetical and one coding for a viral-type inclusion protein), and four decreased in copy number (SNF2 DNA protein and three hypothetical). We also observed single nucleotide polymorphisms, but at a low number. We observed 2950 SNPs in coding genes when comparing the parental and TcK2KO genomes. Most of them (2350) occurred in pseudogenic transcripts (Table S1). From the remaining, 129 changes were predicted to impact protein-coding genes, with most (39) in hypothetical open reading frames. Among them, TcYC6_00871170 codes for a possible kinase that showed an insertion at base 401 producing a frameshift variation. However, the original gene appears truncated in the genome and might correspond to several copies of the Aurora kinase found in the genome and most likely is not responsible for the observed changes. In addition, the kinase coded for TcYC6_0064240 presented a SNP with moderate effects (change in Thr120Ala). The kinase domain of the protein is predicted from AA 573, and the mutation does not appear to change it. These limited modifications are unlikely to produce the observed results.

TcK2 null epimastigotes differentiate into metacyclic-trypomastigotes that infect L6 cells more efficiently than the parental line. However, these intracellular parasites have decreased proliferation and trypomastigote release is significantly delayed and after more than two infection cycles, parasites with unchanged gene reappear. Quantitative whole cell proteomics detected an increase of several proteins normally absent from these stages but expressed in nonproliferative trypomastigotes, such as the trans-sialidase family and paraflagellar rod proteins (38, 39). Most notably, high levels of catalytically active trans-sialidase (group I) proteins were present in the TcK2 null intracellular amastigotes, while proliferative epimastigotes expressed sialidase-like proteins of group II. In addition, we detected alterations in the abundance of metabolic enzymes that are known to be differentially expressed in the different life cycle stages (40, 41, 42). Decreased levels of amastin, a typical amastigote protein, as well as some ribosomal proteins are consistent with the slow growth phenotype of TcK2 null cells and suggest that TcK2 is an important growth-promoting kinase and in its absence a differentiation state predominates.

Our results demonstrating that the absence of TcK2 reduced eIF2α phosphorylation levels in stressed epimastigotes support the notion that TcK2 promotes eIF2α phosphorylation, which is related to differentiation induced by cell stress (10). Accordingly, mutants without the phosphorylated site of eIF2α showed a decreased amastigote growth and reduced release of TCT (11), similarly to what is observed in the absence of TcK2. Furthermore, overexpression of nonphosphorylatable eIF2α or parasites with mutated eIF2α generated by eIF2α gene replacement presented increased levels of trypomastigote-specific genes (11) as observed here. As eIF2α phosphorylation increases intracellular amastigotes that start to undergo the transformation to become trypomastigotes (11), these data provide strong evidence for a direct TcK2 role in promoting growth in opposition to parasite differentiation into infective stages. One might also consider that increased eIF2A in TcK2, an alternative initiation factor, might be relevant to promote trypomastigote translation, but further studies to confirm this possibility are still required.

Analysis of the proteomic data opens some perspectives to understand the role of TcK2 in promoting growth and preventing differentiation. Most notably eIF3a phosphorylation was consistently detected but absent in the TcK2 null lines, suggesting that this translation initiation factor is a direct or a cascade TcK2 substrate. eIF3a is the largest subunit of eIF3 required to assemble the 40S ribosomal subunit with eIF2-GTP-tRNA^met^ to form the 43S subunit (24). eIF3a is also involved in translation termination in mammalian cells and in other processes (43) and can be phosphorylated at multiple residues (44). Interestingly, trypanosomatid eIF3a is structurally distinct from other eukaryotes (45, 46, 47) and possibly related to different mechanisms of translation initiation that might occur in the various stages of the parasite. The phosphorylated site of *T. cruzi* eIF3a occurs in a conserved proteosome domain involved in stabilization of protein–protein interactions. However, it is not present in the human eIF3a subunit. We also noticed the absence of phosphorylated kinesin, a component of the flagellum attachment machinery, in amastigotes of the TcK2 knockout. A respective ortholog is phosphorylated in *T. brucei* and possibly involved in quorum sensing and/or differentiation (48). This phosphorylation occurs in the predicted ATP-binding site of the protein, and it is close to sites that are phosphorylated in the homologous human protein, which might be related to a regulatory activity, considering that large changes in the cytoskeleton take place during differentiation. Our data also showed that TcK2 null epimastigotes had decreased phosphorylation of Cap methyltransferase, PI 4,5 kinases, and the cAMP response protein (CARP4), indicative of involvement in proliferation. Specifically, cAMP is implicated in differentiation in *T. cruzi* (49) and the CAP methyltransferase is linked to mRNA production and translation (50). The phosphorylation of the capping enzyme is in a conserved region of trypanosomatids but does not occur in other eukaryote capping enzymes, which could be related to the unique gene expression regulation of trypanosomes, mostly at the posttranscriptional level (51).

As our goal was also to find scaffolds to be used in drug development, our work allowed identification of two compounds that significantly changed the melting temperature of recombinant TcK2 and were able to inhibit its activity with submicromolar IC_50_. One was Dasatinib, originally developed to inhibit the dual Src/Abl tyrosine protein kinase (52), but with the capacity to bind and inhibit several serine/threonine kinases (53). The second was PF-477736, a checkpoint kinase 1 inhibitor (54). Although the IC_50_ for these inhibitors was higher compared with their original targets, Dasatinib inhibited the proliferation of wildtype intracellular amastigotes of *T. cruzi* with an IC_50_ of 0.6 ± 0.2 μM, while the drug did not affect growth of TcK2 null cells at this concentration, providing strong evidence that TcK2 is one of its main targets. It is unlikely that additional changes in the expression of other kinases could explain the reduced activity of Dasatinib as only one kinase (TcYC6_0087170) was found mutated in TcK2KO but the mutation occurs outside its kinase domain.

**Figure 9:**
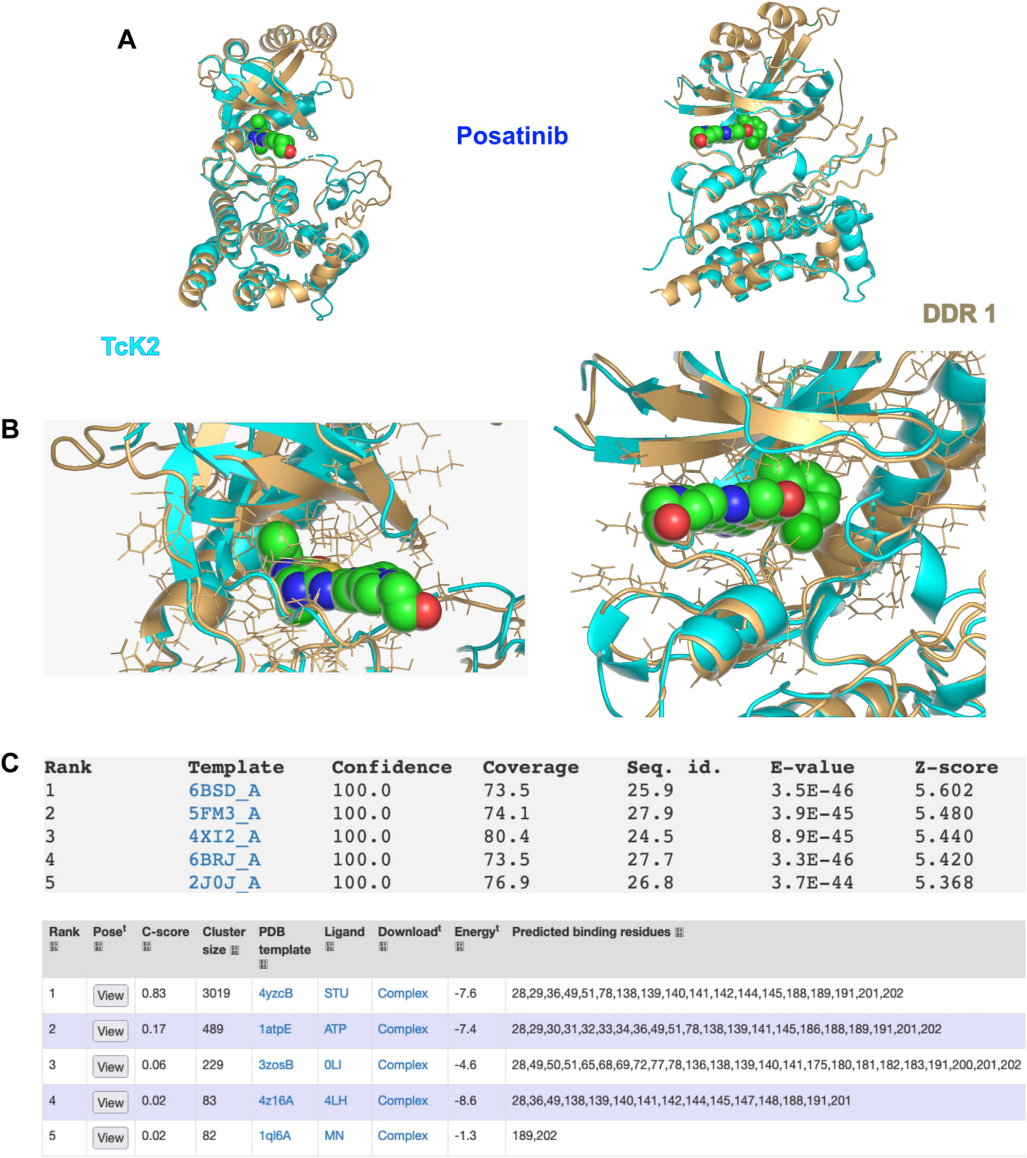
**Model of the TcK2 generated by COACH-D software.***A*, the structure of DDR1 (PDB: 6BSD_A) in savanna color is shown in comparison with the model of TcK2 (KD4) in light blue, both as ribbons generated in the presence of Dasatinib (*spherical*) generated by Pymol. The structures are rotated 90 ° relative to each other to show the position on Dasatinib. *B*, enlargement of the Dasatinib interaction region. *C*, table indicating the best hits for modeling templates. Details of the modeling are in Supporting information file COACH-D results.pdf.

To test the propensity of Dasatinib to bind and inhibit the TcK2 active site we performed a modeling using COACH-D software that searched for binding sites for particular ligands during 3D modeling (55, 56). The results showed a high probability of interaction with the TcK2 model, based on the structures of DDR1 (PDB: 3ZOS) in complex with Ponatinib (Fig. 9 and File S2), a Dasatinib analogue. Altogether, the collective data suggest that Dasatinib can inhibit TcK2 by interacting with the kinase active site. Importantly, known mammalian PERK inhibitors such as GSK2656157 (Fig. S3) and GSK2606414 (not shown) inhibited TcK2 activity only above 1 μM, a concentration that was found toxic for the mammalian cells.

In conclusion, we report the development of an enzymatic assay to screen additional inhibitors of TcK2, a protein kinase that has a unique role in promoting parasite proliferation. The results suggest that derivatives of Dasatinib can be used for Chagas’ disease drug development. Actually, Dasatinib is indicated as an orally available drug for treatment of acute and chronic myeloid leukemias. Treatment is effective because of high sensitivity of a dual Src/Abl tyrosine protein kinase to Dasatinib. However, under current dosing regimens Dasatinib does not reach a serum concentration sufficient for inhibiting *T. cruzi* proliferation (57), and therefore, further improvements are necessary.

## Experimental procedures

### Parasite, mammalian cell cultures and cell invasion assays

*T. cruzi* epimastigotes (Y strain) were maintained in exponential growth phase in liver and infusion tryptose medium (LIT) (58) supplemented with 10% fetal bovine serum (FBS) and 10 U/ml penicillin, 10 μg/ml streptomycin (Invitrogen) at 28 °C. Parasites were counted manually in a Neubauer chamber or with a MUSE equipment (Merck-Millipore). For differentiation, epimastigotes were grown until they reached stationary phase (2 × 10^7^ cells/ml). The parasites were washed and resuspended in Grace’s medium (Thermo Fisher Scientific), and the number of metacyclic-trypomastigotes were estimated by microscopic observation, using phase contrast or after Giemsa staining (11). *T. cruzi* tissue culture–derived trypomastigotes (TCTs) were obtained from supernatants of LLCMK2 cells (Rhesus monkey kidney epithelial cells, ATCC CCL-7), maintained as described (11). The cells were infected with metacyclic-trypomastigotes or with trypomastigotes released from infected cells. To avoid other stages in the trypomastigote cultures, the supernatants were centrifuged at 1500*g* for 5 min, incubated for at least 60 min at 37 °C, and trypomastigote-enriched supernatants collected. Intracellular amastigotes were obtained by scraping the mammalian cells 72 h after infection with a Teflon policeman, followed by mammalian cell rupture with a Potter-Elvehjem tissue grinder in injection buffer (27 mM KH_2_HPO_4_, 8 mM Na_2_HPO_4_, and 26 mM KH_2_PO_4_), and removal of cell debris by centrifugation at 1200*g* for 5 min. Epimastigotes nutritional stress was performed after collecting the cells by centrifugation (2000*g*, for 8 min), resuspension in PBS, centrifugation, and resuspension in 190 mM NaCl, 17 mM KCl, 2 mM CaCl_2_, 2 mM MgCl_2_, and 8 mM KH_2_PO_4_, pH 6.5 (TAU) (59).

For invasion assays, L6 *Rattus norvegicus* skeletal muscle myoblast (L6 cells, CRL-1458, ATCC), L6 cells expressing *T. cruzi* cyclophilin 19 (TcCyp19) (20), or U-2OS cells (Human osteosarcoma cells, Cell Bank, Rio de Janeiro) were seeded either on 13-mm glass coverslips in 24-well plates in 0.5 ml (20,000 cells/well) or in 96-well black plates with clear bottom in 0.1 ml (4000 cells/well), in low-glucose Dulbecco’s modified Eagle’s medium supplemented with 10% FBS and incubated for 24 h at 37 °C and 5% CO_2_. The cells were then incubated with tissue culture trypomastigotes (TCTs) or metacyclic-trypomastigotes (Metas) at the indicated multiplicity of infection in 0.5 or 0.1 ml per well, respectively. For analysis of infection ratio, noninvading parasites were removed after 3 h, wells were washed with PBS, and the cells were fixed with Bouin solution and stained with Giemsa for coverslips (11) or with 4% paraformaldehyde in PBS and stained with Draq5 (Biostatus) for 96-well plates. The percentage of infected cells and the number of remaining cells (cell viability) was determined by counting or by imaging with High Content Analysis System IN Cell Analyzer 2200 (GE), with a 20× magnification as detailed (60). When using Dasatinib (Merck), the compound was added 24 h after infection and incubation continued for the indicated times. The infection rate was normalized to negative control (infected cells, dimethyl sulfoxide [DMSO]-mock treated) and positive control (noninfected cells) to determine the normalized activity using the following equation: [1 - (average [Av]) of the infection ratio test at each compound concentration – Av of infection ratio of the positive control (IRP)/(Av infection ratio negative control – Av IRP)] × 100. Cell ratio was defined as the ratio between the total number of cells in wells upon exposure to the test compound and the average total number of cells from the negative control wells (infected cells, DMSO-mock treated). Benznidazole was used as a positive control. The cell ratio is an evaluation of compound activity against the host cell and was measured to estimate compound selectivity.

To monitor the intracellular growth, the multiplicity of infection was adjusted to obtain the same infectivity for each parasite line. The parasites were incubated for 24 h with the cells; the wells were washed in PBS and incubated with fresh medium for further 48 h. The numbers of intracellular amastigotes were quantified as described (11). In parallel, egress was determined by counting the number of trypomastigotes released in the culture supernatant.

### Cloning, protein expression and purification

The TcK2 (TcYC6_0089220, http://tritrypdb.org) kinase domain was amplified by PCR using total DNA extracted from Y strain epimastigotes (61) with primers F2 and R1 for the KD4 construction or R2 for the KD5 construction, using Phusion DNA polymerase (Thermo Fisher Scientific). All primers used are listed in Table S5. The PCR products were cloned by ligation-independent cloning into the plasmid pNIC28-Bsa4 (Plasmid #2610, Addgene) previously digested with *BsaI*. Both PCR products and the vector were treated with *DpnI* and were gel purified. The products were end-trimmed with T4 DNA polymerase (NEB) in the presence of dCTP for the PCR product, or dGTP for the plasmid, annealed at 37 °C for 30 min, followed by heat shock transformation in MACH1 *E. coli* strain. Colonies were selected in LB-agar supplemented with 5% sucrose and 50 μg/ml kanamycin and screened by colony PCR using pLiC-for and pLiC-rev (Table S5). The correct inserts were confirmed by DNA sequencing.

For protein expression, the plasmids were inserted in BL21(DE3)-lambda-phosphatase bacteria (62), grown in LB-agar containing 50 μg/ml kanamycin and 34 μg/ml chloramphenicol. Protein expression was induced with 0.2 mM IPTG. For preparative purposes, an overnight 50 ml preculture was inoculated in 1.5 L TB medium with 50 μg/ml kanamycin and after reaching absorbance of 2.0 at 600 nm, it was induced with 0.2 mM isopropyl-β-D-thiogalactopyranoside (IPTG) for 16 h at 18 °C. Cells were harvested and resuspended in lysis buffer (50 mM Hepes pH 7.5, 500 mM NaCl, 10% glycerol, 10 mM imidazole pH 7.4, 0.5 mM Tris (2-carboxyethyl-phosphine) [TCEP] [Merck]). Bacterial pellets were resuspended in approximately 35 ml lysis buffer and broken by passages in a French press at 12,000 psi or by sonication at 4 °C in a 5 s on/10 s off program, for a total of 4 min. The cell debris was pelleted at 39,000*g*, for 40 min, and the supernatant was used for purification on a gravity flow Ni-NTA column (4 ml) equilibrated in 50 mM Hepes pH 7.5, 500 mM NaCl, 10% glycerol, 10 mM imidazole pH 7.4, 0.5 mM TCEP (binding buffer). The column was washed with 80 ml of the same buffer containing 30 mM imidazole, and the protein was eluted in the buffer containing 300 mM imidazole pH 7.4. The eluted fractions obtained at 1.0 ml/min were concentrated to 5 ml with a 30 kDa MWCO spin concentrator and chromatographed into a Superdex S200 16/60 column (Cytiva), pre-equilibrated in 20 mM Hepes pH 7.5, 300 mM NaCl, 1 mM TCEP, 5% glycerol. Fractions containing the main peak were collected, and SDS-PAGE gels were used to assess the purity of the sample. The obtained TcK2 proteins were concentrated to 10 mg/ml, aliquoted, and stored at −70 °C until use. The human recombinant protein corresponding to PERK kinase domain (PERK-KD) in fusion with glutathione-S-transferase was obtained by expression in *E. coli* DE3 (Artic Express) transformed with pGEX4T1-PERK-KD from David Ron (Addgene, Plasmid #21817). The protein was expressed in LB medium containing 100 μg/ml ampicillin until absorbance of 0.6 at 600 nm induced with 1 mM IPTG at 12 °C for 18 h. The purification was performed from the soluble extract, after French press lysis in GST-agarose (Cytiva) equilibrated in 50 mM Tris-HCl, 0.1 M NaCl, 0.15 mM phenyl-methyl sulfonyl fluoride, 1 μg/ml pepstatin, and 10 μg/ml leupeptin, pH 7.4. The resin was washed with 20 column volumes, and the enzyme was eluted in 0.1 M reduced glutathione in 50 mM Tris-HCl, pH 7.4. Mouse eIF2α cloned in pET28a (9) was expressed in *E. coli* BL21 (Artic Express) at 12 °C, for 18 h, after induction with 1 mM IPTG. After lysis on French press, the soluble proteins were purified by affinity on Ni^2+^ agarose equilibrated in 20 mM Tris-HCl, 500 mM NaCl, 10 mM imidazole, and Complete Mini EDTA-free Easy Pack (Roche) at pH 7.4. The protein was eluted with the same buffer containing 300 mM imidazole at pH 6.0, concentrated by filtration in 10 kDa MWCO spin concentrator, and applied to a Superdex S200 column (10 × 300) in 20 mM Tris-HCl, 500 mM NaCl. The fractions corresponding to the monomer size were confirmed by mass spectrometry and further used in the assays.

### Western blot

Whole parasite extracts were prepared by collecting epimastigote forms and boiling in denaturing sample buffer (25 mM Tris-HCl, pH 6.8, 2% SDS, 6% (v/v) glycerol, 2 mM DTT, 0.01% (w/v) bromophenol blue). Extracts were resolved on 8% polyacrylamide gels and transferred to nitrocellulose or PVDF membranes (Bio-Rad) by standard procedures. The membranes were stained with 0.3% Ponceau S (w/v) in 0.3% (v/v) acetic acid, washed in water, and blocked with 3% (w/v) bovine serum albumin (BSA) or milk in 10 mM Tris-HCl, pH 7.4, 0.15 M NaCl, and 0.05% Tween-20 (TBS-T). Primary antibody incubation was performed for 1 h at room temperature with TBS-T supplemented with 3% BSA, diluted as described below. Membranes were washed with TBS-T and then incubated with secondary antibodies in the same conditions as the primary antibodies. IRDye-conjugated secondary antibodies (anti-rabbit or anti-mouse IgG coupled to IRDye 800 and IRDye 680 LI-COR Biosciences) were used for protein detection and visualization. Alternatively, secondary antibodies conjugated to peroxidase were used (Thermo Fisher Scientific), and detection was performed by chemiluminescence reagent (Merck). Proteins were detected using immunopurified anti-TcK2 antibodies, raised in rabbits that were immunized with three intradermic injections of 200 μg of recombinant TcKD4 protein in Freund’s adjuvant, with 3 weeks interval. After the immunization, specific antibodies were obtained by adsorption on TcK2 protein coupled to CNBr-Sepharose resin (Cytiva) and elution with 50 mM glycine pH 2.5, followed by neutralization with 1 M Tris-HCl pH 8.5. Rabbit antiserum against HSP70 (anti-HSP70) was donated by James Bangs (63). Affinity purified, rabbit anti-phosphorylated eIF2α and mouse anti-eIF2α from *T. cruzi* were obtained as described (11) and used at a dilution of 1:100 and 1:1000, respectively. Rabbit anti-mouse eIF2αS51 (Thermo Fischer Scientific, #44-728G) was diluted 1:5000, and total mouse anti-eIF2α (Thermo Fisher, # AHO1182) was used at 1:2000.

### Generation of TcK2KO lines

Epimastigotes (Y strain) expressing Cas9 were generated by transfection with plasmid pTREX-Cas9-Neo (64), selected, and maintained in LIT medium, 10% FBS, supplemented with 200 μg/ml Geneticin G418. Exponentially growing Cas9 epimastigotes were collected and centrifuged at 2000*g* for 5 min; the parasites were washed in 1 ml of electroporation buffer (5 mM KCl, 0.15 mM CaCl_2_, 90 mM Na_2_HPO_4_, 50 mM Hepes, 50 mM mannitol) (65) and resuspended in 100 μl of the same buffer, along with 50 μl containing 2 μg of purified sgRNA and 1 μmole of the donor sequence. The sgRNA was generated using primers TcK2 sg34 defined as described in http://grna.ctegd.uga.edu/ (66) and the common primer that recognizes the plasmid pUC sgRNA (64) (Table S5). The sgRNA was prepared by *in vitro* transcription using the MEGAshortscript Kit (Thermo Fisher Scientific) according to the manufacturer instructions and purified by phenol/chloroform extraction and ethanol precipitation. The donors for the insertion of stop codon was the primer TcK2 donor 34 that contained a stop codon and the site *EcoRV*. The insert of BSD donor was prepared by PCR amplification of the plasmid pTREX-BSD (64), with primers TcK2-KO-BSD Fdonor and TcK2-KO-BSD Rdonor (Table S5) and used after agarose gel electrophoresis purification. Parasites were transfected in 2-mm cuvettes using AMAXA Nucleofactor II electroporator, with two pulses of X-014 program in Cytomix buffer (2 mM EGTA, 120 mM KCl, 0.15 mM CaCl_2_, 10 mM potassium phosphate pH 7.6, 25 mM Hepes, 1 mM hypoxanthine, 5 mM MgCl_2_, 5 mg/ml glucose, 0.1 mg/ml BSA). Subsequently, parasites were transferred to bottles with fresh LIT medium supplemented with 10% FBS and maintained at 28 °C for recovery. To verify the correct insertion, the total DNA of the parasites were extracted and the region corresponding to TcK2 was PCR-amplified using the primers K2-For and K2-Rev and digested for 3 h with *EcoRV* followed by agarose gel electrophoresis and ethidium bromide staining. Parasite clones were obtained by serial dilutions (2, 1, 0.5, and 0.25 per well) in 96-well plates in complete LIT medium. The wells with growing parasites in the most diluted wells were considered clones. Reactive oxygen species were measured in parasites and mammalian cells using CM-H2DCFDA (Thermo Fisher Scientific) as described (20).

### Genomic analysis

For the genomic analysis, the DNA was obtained from epimastigotes of the Y strain expressing T7 RNA polymerase and Cas9 endonuclease and a clone of the parental line with the TcK2 gene replaced by the stop codon region by the Blood GenomicPrep Mini Spin Kit (Cytiva). Illumina libraries and sequencing was performed by Novogene Incorporation Inc. The genomic DNA was fragmented, end-repaired and A-tailed, ligated to 5′ and 3′ adapters, and PCR amplified, and the generated fragments were size purified. The sequences were made in the NovaSeq PE150. The distribution of the sequencing error used single base substitution technology for the high-throughput sequencing platform (67) was 0.3% from 96,980,522 and 91,864,014 reads, respectively, for each genomic library of Cas9 and TcK2KO. The original raw data from Illumina platform were transformed to Sequenced Reads, known as Raw Data or RAW Reads, by base calling. Raw data are recorded in a FASTQ file, which contained sequencing reads and corresponding sequencing quality (68). The analysis of the FASTQ files were performed using the VEuPathDB Galaxy site (https://veupathdb.globusgenomics.org) using the Variant Calling Workflow for paired end reads (v.7), which consists of the Galaxy Version FASTQC 0.11.3, Galaxy Sickle Version 1.33.2, and Galaxy Bowtie2.4.4), using as reference genome the TriTrypDB_46 *T. cruzi* YC6_Genome. Variants were analyzed by Galaxy Version FreeBayes-6, v 0.9.21-19-gc003c1e with the same genome and the quality was filtered with the Galaxy quality–7, version 1.0.0, and single nucleotide polymorphisms were identified using the Galaxy Version SNPEFF 3.6. The BAM files generated from Bowtie analysis were visualized and analyzed using Geneious Prime 2022.1.1. The hits were assigned to the assembled YC6 genome previously inserted in the Geneious program using the corresponding fasta and gff files obtained from the TriTrypDB database. The sequencing data are available in the BioProject at NCBI (PRJNA939735). For each contig, the number of fragment hits was obtained. We found no differences between the original YC6 and the Cas9 lines hit profile. However, appreciable changes were detected in comparison of our lines with the deposited genome (Fig. S1). Therefore, the raw partial counts per CDS for each contig were obtained relative to the total read counts and differences for each CDS calculated using the Geneious software. The results were exported as. csv files and analyzed and visualized using Orange3 3.32 (https://orangedatamining.com). Similarly, the .vcf files obtained from the SNPEFF analysis were imported into Geneious that was used to identify the differential SNPs between YC6 Cas9 and the YC6 TcK2KO.

### Proteomic analysis

Parasites (2 × 10^7^/ml) were collected by centrifugation (2000*g*, 3 min), washed once in PBS, resuspended in 20 μl of SDS-PAGE sample buffer containing 5 mM DTT, and boiled for 5 min before loading onto precast Novex Value 4 to 12% Tris-Glycine gels (Thermo Fisher). The electrophoresis was performed in 50 mM Mops, 50 mM Tris base, 3.5 mM SDS, 1 mM EDTA, at 100 V for 10 min. The gel region corresponding to the migration portion of each lane was cut, and the gel pieces were fixed 3 times in 1 volume of 40% ethanol, 10% acetic acid, 10 min each at room temperature. The bands were kept frozen at −80 °C until processed by mass spectrometry analysis. The gel slices were subjected to reductive alkylation and in-gel tryptic digest using routine procedures. The eluted peptides were then analyzed by liquid chromatography–tandem mass spectrometry on an ultimate 3000 nano rapid separation LC system (Dionex) coupled to a Q Exactive HF Hybrid Quadrupole-Orbitrap mass spectrometer (Thermo Fisher Scientific). Spectra were processed using the intensity-based label-free quantification method of MaxQuant (69) searching the *T. cruzi* YC6 annotated protein database from TriTrypDB and S and T phosphopeptides (16). The label-free quantification data were analyzed using Perseus software (70). A Student’s *t* test was used to compare the control sample groups with the respective modified strain generated -log10 *t* test *p*-value plotted *versus* log of the difference between assigned samples and was used to generate a volcano plot. Enrichment analysis was performed using Fisher's exact test in Perseus with a Benjamin–Hochberg false discovery rate of 0.02. Proteomics data have been deposited to the ProteomeXchange Consortium *via* the PRIDE partner repository (71) with the dataset identifier PXD040597.

### Differential scanning fluorimetry

Differential scanning fluorometry (DSF) was performed in 384-well plates using QuantStudio 6 Real-Time PCR Software (Applied Biosystems) with excitation and emission filters of 492 and 610 nm, respectively. Each well contained 2 μM protein in 20 μl DSF buffer (20 mM Hepes pH 7.5, 300 mM NaCl, 1 mM TCEP, 5% glycerol), 2 μl SYPRO ORANGE (Merck) diluted 1000-fold in DSF buffer, and 2 μl ligand at 1 mM. Fluorescence intensities were measured from 25 to 96 °C with a ramp rate of 3 °C/min using a C1000 thermal cycler with CFX-96 RT-PCR head (Bio-Rad). The compounds were obtained from a kinase inhibitor library (Selleckchem.com, detailed in Table S4).

### Kinase assays

The kinase phosphorylation activity on mouse eIF2α by TcK2 (KD4 at 100 nM) was done in a final volume of 27 μl, containing 0.25 M Hepes, pH 7.0, 0.05% Nonidet P40, 50 mM MgCl_2_, 5 mM EGTA, and 10 μM ATP at 37 °C. The reactions also contained 1.6 μM (56 μg/ml) of recombinant mouse eIF2α prepared as described (9). Assays were also performed in the presence of GSK2656157 (MedChemExpress), hemin (Merck). At the end of the incubation period, the reactions were stopped by addition of 2× SDS-PAGE sample buffer, heated at 96 °C for 5 min, and submitted to Western blot as described above.

The activity of TcK2 recombinant proteins (KD4 and KD5) was measured by LANCE Ultra Kinase Assay (PerkinElmer, TRF0200-D, TRF0107-D; TRF0214-D, TRF0126-D), with detection by fluorescence in 384-well plates. This assay gave a linear response with protein concentration from 0.244 to 500 nM. A typical reaction consisted of 400 nM of recombinant TcK2, 50 nM of substrate (CREBtide, p70S6K, and histone H3 peptides), 1.7 μM of ATP, and 1× kinase buffer (50 mM Hepes pH 7.5, 10 mM MgCl_2_, 0.01% Tween 20). The assay was performed in two steps: the enzymatic reaction was left for 2 to 3 h at 25 °C, followed by addition of 1 mM EDTA and incubation for 5 min. Then, the respective Europium-conjugated antibody was added and the plate was incubated at room temperature for 2 h for the reaction detection. The time-resolved fluorescence energy transfer (TR-FRET) signal corresponding to the amount of phosphorylated peptide was measured using ClarioStar microplate reader (BMG Labtech) previously set up to 340 nm excitation and 665 nm emission wavelengths, 50 μs delay time, 100 flashes, and 100 μs integration time.

### Data analysis and statistics

Results are expressed as the mean and SD indicating the number of replicates in each experiment. The statistical analysis was performed using the tests and significance indicated in each experiment using Prism9 (GraphPad).

## Data availability

All data are available upon request and shared to public data banks indicated in the text.

## Supporting information

This article contains supporting information.

## Conflict of interest

The authors declare that they have no conflicts of interest with the contents of this article.

## Supporting information

Table S1 Differential detection of CDS levels (Copy variation) including the relative numbers to the genes in the genome of TcYC6 in https://tritrypdb.org, and single nucleotide polymorphisms (SNPs) differences in Cas9 and TcK2KO.

Table S2 Perseus analysis of proteomic data of Cas9 and TcK2KO samples of epimastigotes (Epi), TCT and amastigotes (AMA).

Table S3 Perseus analysis of serine and threonine phosphorylation data of Cas9 and TcK2KO samples epimastigotes (Epi), TCT and amastigotes (AMA).

Table S4 Selleckchem compounds and DSF analysis of TcK2 KD5 showing the Tm and ΔTm for each tested compound.

Table S5 Oligonucleotides used in this work.

COACH-D results

TcK2-KD4 Report

tmodel1.pdb

tmodel2.pdb

tmodel3

tmodel4

tmodel5
