# Supplementary material for: Identification of inhibitors for the transmembrane Trypanosoma cruzi eIF2α kinase relevant for parasite proliferation
Source: J Biol Chem. 2023 May 23;299(7):104857. doi: 10.1016/j.jbc.2023.104857 (PMC10300260; doi:10.1016/j.jbc.2023.104857)
Supplement: Table S5 — Oligonucleotides used in this work. [file mmc5.docx]

Table S5 – Oligonucleotides used in this work

| Oligonucletide | Sequence |
| --- | --- |
| F2 | 5’TACTTCCAATCCATGTCTCAACTTTTTCAGCAGCAGTTCC |
| R1 | 5’TATCCACCTTTACTGTCACTCCTCCTCCTCCTTAATTCC |
| R2 | 5’TATCCACCTTTACTGTCAAATTCCCTGTCGCAGGGC |
| TcK2 sg34 | 5’GGAGGCCGGAGAATTGTAATACGACTCACTATAGGGAGAGATCCTGTGCATATTGACCCTGTTTTAGAGCTAGAAATAGC |
| Tck2 donor 34 | 5’ATTGGGCTCCTAATCCTGTGCATATTGACCTAGATATCTAGCTTGGGCTCTCGTTCGGTGTGGGTAGCACG |
| Tck2-KO-BSD F donor | 5’CTACGGCTATCCCTGTGTTTGTCCCTGCGTGTGAGAATTACACTGGAGGGTTGTGCCTCCTCTGTGGCATTTTTCCCTAAATGGCCAAGCCTTTGTCTCA |
| Tck2-KO-BSD R donor | 5’TTCAGCAAATTAGCATCCTGCGCATGAGCCTGGTCTCCGGAGACAGATAGGAGAGTCGTGCTACCCACACCGAACGAGAGTTAGCCCTCCCACACATAAC |
| Common | 5’AAAAAAGCACCGACTCGGTGCCACTT |
| K2-For | 5’CCGAGCTCCCGGGTTGGCACAGTCGATCGCACC |
| K2-Rev | 5’CTCGAGTCACCCCTTTTGAAGCAGGCTCTGAAG |
| pLIC-for | 5’TGTGAGCGGATAACAATTCC |
| pLic-rev | 5’AGCAGCCAACTCAGCTTCC |
