# Supplementary material for: Identification of inhibitors for the transmembrane Trypanosoma cruzi eIF2α kinase relevant for parasite proliferation
Source: J Biol Chem. 2023 May 23;299(7):104857. doi: 10.1016/j.jbc.2023.104857 (PMC10300260; doi:10.1016/j.jbc.2023.104857)
Supplement: COACH-D results [file mmc6.pdf]

Predicted ligand-binding sites and poses

Top 5 predicitons

Rank		C-score	Cluster size	PDB template	Ligand	Download	Energy	Predicted binding residues
1		0.83	3019	4yzcB (https://www.rcsb.org/structure/4yzc)	STU (https://zhanglab.ccmb.med.umich.edu/BioLiP/sym.cgi?code=STU)	Complex tmodel1.pdb	-7.6	28,29,36,49,51,78,138,139,140,141,142,144,145,188,189,191,201,202
2		0.17	489	1atpE (https://www.rcsb.org/structure/1atp)	ATP (https://zhanglab.ccmb.med.umich.edu/BioLiP/sym.cgi?code=ATP)	Complex tmodel2.pdb	-7.4	28,29,30,31,32,33,34,36,49,51,78,138,139,141,145,186,188,189,191,201,202
3		0.06	229	3zosB (https://www.rcsb.org/structure/3zos)	0LI (https://zhanglab.ccmb.med.umich.edu/BioLiP/sym.cgi?code=0LI)	Complex tmodel3.pdb)	-4.6	28,49,50,51,65,68,69,72,77,78,136,138,139,140,141,175,180,181,182,183,191,200,201,202
4		0.02	83	4z16A (https://www.rcsb.org/structure/4z16)	4LH (https://zhanglab.ccmb.med.umich.edu/BioLiP/sym.cgi?code=4LH)	Complex tmodel4.pdb	-8.6	28,36,49,138,139,140,141,142,144,145,147,148,188,191,201
5		0.02	82	1ql6A (https://www.rcsb.org/structure/1ql6)	MN (https://zhanglab.ccmb.med.umich.edu/BioLiP/sym.cgi?code=MN)	Complex tmodel5.pdb	-1.3	189,202

predicted_bsr.txt = the detailed prediction summary.
dock/clustering.txt the templates clustering results.

Summary for ligands in the first-ranked prediciton above

Rank	Ligand	Frequency	PDB template	Download	Visualizaiton
1	ANP (https://zhanglab.ccmb.med.umich.edu/BioLiP/sym.cgi?code=ANP)	153	1ad5A (https://www.rcsb.org/structure/1ad5), 1ad5B (https://www.rcsb.org/structure/1ad5), 1cm8A (https://www.rcsb.org/structure/1cm8), 1ir3A (https://www.rcsb.org/structure/1ir3), 1j1bA (https://www.rcsb.org/structure/1j1b),<br>1j1bB (https://www.rcsb.org/structure/1j1b), 1jpaB (https://www.rcsb.org/structure/1jpa), 1jwhA (https://www.rcsb.org/structure/1jwh), 1mqbA (https://www.rcsb.org/structure/1mqb), 1o6kA (https://www.rcsb.org/structure/1o6k),<br>1pjkA (https://www.rcsb.org/structure/1pjk), 1pyxB (https://www.rcsb.org/structure/1pyx), 1qpcA (https://www.rcsb.org/structure/1qpc), 1xr1A (https://www.rcsb.org/structure/1xr1), 1yxtA (https://www.rcsb.org/structure/1yxt),<br>1zy5A (https://www.rcsb.org/structure/1zy5), 1zy5B (https://www.rcsb.org/structure/1zy5), 2a19B (https://www.rcsb.org/structure/2a19), 2a19C (https://www.rcsb.org/structure/2a19), 2bzkB (https://www.rcsb.org/structure/2bzk),	SDF file (https://yanglab.nankai.edu.cn/SDF/ANP.sdf)	
2	STU (https://zhanglab.ccmb.med.umich.edu/BioLiP/sym.cgi?code=STU)	97	1aq1A (https://www.rcsb.org/structure/1aq1), 1bygA (https://www.rcsb.org/structure/1byg), 1nxkA (https://www.rcsb.org/structure/1nxk), 1nxkD (https://www.rcsb.org/structure/1nxk), 1okyA (https://www.rcsb.org/structure/1oky),<br>1q3dA (https://www.rcsb.org/structure/1q3d), 1q3dB (https://www.rcsb.org/structure/1q3d), 1qpdA (https://www.rcsb.org/structure/1qpd), 1qpjA (https://www.rcsb.org/structure/1qpj), 1sm2A (https://www.rcsb.org/structure/1sm2),<br>1snuA (https://www.rcsb.org/structure/1snu), 1stcE (https://www.rcsb.org/structure/1stc), 1u59A (https://www.rcsb.org/structure/1u59), 1wvyA (https://www.rcsb.org/structure/1wvy), 1xbcA (https://www.rcsb.org/structure/1xbc),<br>1xjdA (https://www.rcsb.org/structure/1xjd), 1yhsA (https://www.rcsb.org/structure/1yhs), 2bujA (https://www.rcsb.org/structure/2buj), 2bujB (https://www.rcsb.org/structure/2buj), 2clqA (https://www.rcsb.org/structure/2clq),	SDF file (https://yanglab.nankai.edu.cn/SDF/STU.sdf)	
3	ADP (https://zhanglab.ccmb.med.umich.edu/BioLiP/sym.cgi?code=ADP)	64	1j1cA (https://www.rcsb.org/structure/1j1c), 1j1cB (https://www.rcsb.org/structure/1j1c), 1ol5A (https://www.rcsb.org/structure/1ol5), 1rdqE (https://www.rcsb.org/structure/1rdq), 2b9fA (https://www.rcsb.org/structure/2b9f),<br>2b9hA (https://www.rcsb.org/structure/2b9h), 2g2iA (https://www.rcsb.org/structure/2g2i), 2g2iB (https://www.rcsb.org/structure/2g2i), 2henB (https://www.rcsb.org/structure/2hen), 2qurA (https://www.rcsb.org/structure/2qur),<br>3brbA (https://www.rcsb.org/structure/3brb), 3brbB (https://www.rcsb.org/structure/3brb), 3d5wA (https://www.rcsb.org/structure/3d5w), 3dlsA (https://www.rcsb.org/structure/3dls), 3dlsD (https://www.rcsb.org/structure/3dls),<br>3eqhA (https://www.rcsb.org/structure/3eqh), 3f5gA (https://www.rcsb.org/structure/3f5g), 3f61A (https://www.rcsb.org/structure/3f61), 3gu6A (https://www.rcsb.org/structure/3gu6), 3p23D (https://www.rcsb.org/structure/3p23),	SDF file (https://yanglab.nankai.edu.cn/SDF/ADP.sdf)	
Others	ATP (https://zhanglab.ccmb.med.umich.edu/BioLiP/sym.cgi?code=ATP)(63), LDN (https://zhanglab.ccmb.med.umich.edu/BioLiP/sym.cgi?code=LDN)(32), ACP (https://zhanglab.ccmb.med.umich.edu/BioLiP/sym.cgi?code=ACP)(30), AMP (https://zhanglab.ccmb.med.umich.edu/BioLiP/sym.cgi?code=AMP)(24), P4O (https://zhanglab.ccmb.med.umich.edu/BioLiP/sym.cgi?code=P4O)(18), B49 (https://zhanglab.ccmb.med.umich.edu/BioLiP/sym.cgi?code=B49)(17), 1N1 (https://zhanglab.ccmb.med.umich.edu/BioLiP/sym.cgi?code=1N1)(16), IZA (https://zhanglab.ccmb.med.umich.edu/BioLiP/sym.cgi?code=IZA)(15), M77 (https://zhanglab.ccmb.med.umich.edu/BioLiP/sym.cgi?code=M77)(14), AGS (https://zhanglab.ccmb.med.umich.edu/BioLiP/sym.cgi?code=AGS)(14), GVD (https://zhanglab.ccmb.med.umich.edu/BioLiP/sym.cgi?code=GVD)(14), DB8 (https://zhanglab.ccmb.med.umich.edu/BioLiP/sym.cgi?code=DB8)(13), MK2 (https://zhanglab.ccmb.med.umich.edu/BioLiP/sym.cgi?code=MK2)(12), JZO (https://zhanglab.ccmb.med.umich.edu/BioLiP/sym.cgi?code=JZO)(12), IQU (https://zhanglab.ccmb.med.umich.edu/BioLiP/sym.cgi?code=IQU)(12),<br>MIX (https://zhanglab.ccmb.med.umich.edu/BioLiP/sym.cgi?code=MIX)(11), Y27 (https://zhanglab.ccmb.med.umich.edu/BioLiP/sym.cgi?code=Y27)(10), VX6 (https://zhanglab.ccmb.med.umich.edu/BioLiP/sym.cgi?code=VX6)(9), ADN (https://zhanglab.ccmb.med.umich.edu/BioLiP/sym.cgi?code=ADN)(9), ZOP (https://zhanglab.ccmb.med.umich.edu/BioLiP/sym.cgi?code=ZOP)(9), 55F (https://zhanglab.ccmb.med.umich.edu/BioLiP/sym.cgi?code=55F)(9), APJ (https://zhanglab.ccmb.med.umich.edu/BioLiP/sym.cgi?code=APJ)(9), 3AM (https://zhanglab.ccmb.med.umich.edu/BioLiP/sym.cgi?code=3AM)(9), BI1 (https://zhanglab.ccmb.med.umich.edu/BioLiP/sym.cgi?code=BI1)(9), FEF (https://zhanglab.ccmb.med.umich.edu/BioLiP/sym.cgi?code=FEF)(8), 3NC (https://zhanglab.ccmb.med.umich.edu/BioLiP/sym.cgi?code=3NC)(8), P16 (https://zhanglab.ccmb.med.umich.edu/BioLiP/sym.cgi?code=P16)(8), DKI (https://zhanglab.ccmb.med.umich.edu/BioLiP/sym.cgi?code=DKI)(8), 4SP (https://zhanglab.ccmb.med.umich.edu/BioLiP/sym.cgi?code=4SP)(7), H52 (https://zhanglab.ccmb.med.umich.edu/BioLiP/sym.cgi?code=H52)(7),<br>PDY (https://zhanglab.ccmb.med.umich.edu/BioLiP/sym.cgi?code=PDY)(7), BI8 (https://zhanglab.ccmb.med.umich.edu/BioLiP/sym.cgi?code=BI8)(7), TAK (https://zhanglab.ccmb.med.umich.edu/BioLiP/sym.cgi?code=TAK)(7), KS1 (https://zhanglab.ccmb.med.umich.edu/BioLiP/sym.cgi?code=KS1)(7), 824 (https://zhanglab.ccmb.med.umich.edu/BioLiP/sym.cgi?code=824)(7), 1RO (https://zhanglab.ccmb.med.umich.edu/BioLiP/sym.cgi?code=1RO)(7), VGH (https://zhanglab.ccmb.med.umich.edu/BioLiP/sym.cgi?code=VGH)(7), MPY (https://zhanglab.ccmb.med.umich.edu/BioLiP/sym.cgi?code=MPY)(7), QUE (https://zhanglab.ccmb.med.umich.edu/BioLiP/sym.cgi?code=QUE)(6), XDR (https://zhanglab.ccmb.med.umich.edu/BioLiP/sym.cgi?code=XDR)(6), ZZL (https://zhanglab.ccmb.med.umich.edu/BioLiP/sym.cgi?code=ZZL)(6), YUN (https://zhanglab.ccmb.med.umich.edu/BioLiP/sym.cgi?code=YUN)(6), BX7 (https://zhanglab.ccmb.med.umich.edu/BioLiP/sym.cgi?code=BX7)(6), 324 (https://zhanglab.ccmb.med.umich.edu/BioLiP/sym.cgi?code=324)(6), 4ST (https://zhanglab.ccmb.med.umich.edu/BioLiP/sym.cgi?code=4ST)(6),<br>SM5 (https://zhanglab.ccmb.med.umich.edu/BioLiP/sym.cgi?code=SM5)(6), SX7 (https://zhanglab.ccmb.med.umich.edu/BioLiP/sym.cgi?code=SX7)(6), CK2 (https://zhanglab.ccmb.med.umich.edu/BioLiP/sym.cgi?code=CK2)(6), 05B (https://zhanglab.ccmb.med.umich.edu/BioLiP/sym.cgi?code=05B)(5), UCN (https://zhanglab.ccmb.med.umich.edu/BioLiP/sym.cgi?code=UCN)(5), 7CP (https://zhanglab.ccmb.med.umich.edu/BioLiP/sym.cgi?code=7CP)(5), 627 (https://zhanglab.ccmb.med.umich.edu/BioLiP/sym.cgi?code=627)(5), P06 (https://zhanglab.ccmb.med.umich.edu/BioLiP/sym.cgi?code=P06)(5), RFZ (https://zhanglab.ccmb.med.umich.edu/BioLiP/sym.cgi?code=RFZ)(5), IRE (https://zhanglab.ccmb.med.umich.edu/BioLiP/sym.cgi?code=IRE)(5), C4E (https://zhanglab.ccmb.med.umich.edu/BioLiP/sym.cgi?code=C4E)(5), AZ5 (https://zhanglab.ccmb.med.umich.edu/BioLiP/sym.cgi?code=AZ5)(4), PP2 (https://zhanglab.ccmb.med.umich.edu/BioLiP/sym.cgi?code=PP2)(4), 3RA (https://zhanglab.ccmb.med.umich.edu/BioLiP/sym.cgi?code=3RA)(4), MFR (https://zhanglab.ccmb.med.umich.edu/BioLiP/sym.cgi?code=MFR)(4),<br>MP6 (https://zhanglab.ccmb.med.umich.edu/BioLiP/sym.cgi?code=MP6)(4), Z92 (https://zhanglab.ccmb.med.umich.edu/BioLiP/sym.cgi?code=Z92)(4), ADE (https://zhanglab.ccmb.med.umich.edu/BioLiP/sym.cgi?code=ADE)(4), BK3 (https://zhanglab.ccmb.med.umich.edu/BioLiP/sym.cgi?code=BK3)(4), NM7 (https://zhanglab.ccmb.med.umich.edu/BioLiP/sym.cgi?code=NM7)(4), 0CK (https://zhanglab.ccmb.med.umich.edu/BioLiP/sym.cgi?code=0CK)(4), 877 (https://zhanglab.ccmb.med.umich.edu/BioLiP/sym.cgi?code=877)(4), 626 (https://zhanglab.ccmb.med.umich.edu/BioLiP/sym.cgi?code=626)(4), IXM (https://zhanglab.ccmb.med.umich.edu/BioLiP/sym.cgi?code=IXM)(4), DJK (https://zhanglab.ccmb.med.umich.edu/BioLiP/sym.cgi?code=DJK)(4), 5CP (https://zhanglab.ccmb.med.umich.edu/BioLiP/sym.cgi?code=5CP)(4), 23D (https://zhanglab.ccmb.med.umich.edu/BioLiP/sym.cgi?code=23D)(4), 985 (https://zhanglab.ccmb.med.umich.edu/BioLiP/sym.cgi?code=985)(4), J60 (https://zhanglab.ccmb.med.umich.edu/BioLiP/sym.cgi?code=J60)(4), AWR (https://zhanglab.ccmb.med.umich.edu/BioLiP/sym.cgi?code=AWR)(4),<br>DW1 (https://zhanglab.ccmb.med.umich.edu/BioLiP/sym.cgi?code=DW1)(4), ZRK (https://zhanglab.ccmb.med.umich.edu/BioLiP/sym.cgi?code=ZRK)(4), 537 (https://zhanglab.ccmb.med.umich.edu/BioLiP/sym.cgi?code=537)(4), NVB (https://zhanglab.ccmb.med.umich.edu/BioLiP/sym.cgi?code=NVB)(4), 5PW (https://zhanglab.ccmb.med.umich.edu/BioLiP/sym.cgi?code=5PW)(4), IYZ (https://zhanglab.ccmb.med.umich.edu/BioLiP/sym.cgi?code=IYZ)(4), BK1 (https://zhanglab.ccmb.med.umich.edu/BioLiP/sym.cgi?code=BK1)(4), GW8 (https://zhanglab.ccmb.med.umich.edu/BioLiP/sym.cgi?code=GW8)(4), 859 (https://zhanglab.ccmb.med.umich.edu/BioLiP/sym.cgi?code=859)(4), DTQ (https://zhanglab.ccmb.med.umich.edu/BioLiP/sym.cgi?code=DTQ)(4), G98 (https://zhanglab.ccmb.med.umich.edu/BioLiP/sym.cgi?code=G98)(4), 4H5 (https://zhanglab.ccmb.med.umich.edu/BioLiP/sym.cgi?code=4H5)(4), PFQ (https://zhanglab.ccmb.med.umich.edu/BioLiP/sym.cgi?code=PFQ)(4), KSA (https://zhanglab.ccmb.med.umich.edu/BioLiP/sym.cgi?code=KSA)(4), 215 (https://zhanglab.ccmb.med.umich.edu/BioLiP/sym.cgi?code=215)(4),<br>ZC3 (https://zhanglab.ccmb.med.umich.edu/BioLiP/sym.cgi?code=ZC3)(4), FP3 (https://zhanglab.ccmb.med.umich.edu/BioLiP/sym.cgi?code=FP3)(4), SR2 (https://zhanglab.ccmb.med.umich.edu/BioLiP/sym.cgi?code=SR2)(4), 679 (https://zhanglab.ccmb.med.umich.edu/BioLiP/sym.cgi?code=679)(4), 893 (https://zhanglab.ccmb.med.umich.edu/BioLiP/sym.cgi?code=893)(4), VAR (https://zhanglab.ccmb.med.umich.edu/BioLiP/sym.cgi?code=VAR)(4), 3RH (https://zhanglab.ccmb.med.umich.edu/BioLiP/sym.cgi?code=3RH)(4),				

References

- Q Wu, Z Peng, Y Zhang, J Yang, *COACH-D: improved protein-ligand binding site prediction with refined ligand-binding poses through molecular docking*, **Nucleic Acids Research**, 46: W438–W442 (2018).
- J Yang, A Roy, Y Zhang, *Protein-ligand binding site recognition using complementary binding-specific substructure comparison and sequence profile alignment*, **Bioinformatics**, 29: 2588-2595 (2013).
