# Supplementary material for: Identification of inhibitors for the transmembrane Trypanosoma cruzi eIF2α kinase relevant for parasite proliferation
Source: J Biol Chem. 2023 May 23;299(7):104857. doi: 10.1016/j.jbc.2023.104857 (PMC10300260; doi:10.1016/j.jbc.2023.104857)
Supplement: TcK2-KD4 Report [file mmc7.pdf]

SWISS-MODEL Homology Modelling Report

Model Building Report

This document lists the results for the homology modelling project "TcK2-KD4" submitted to SWISS-MODEL workspace on Jan. 1, 2023, 4:27 p.m..The submitted primary amino acid sequence is given in Table T1.

If you use any results in your research, please cite the relevant publications:

- Waterhouse, A., Bertoni, M., Bienert, S., Studer, G., Tauriello, G., Gumienny, R., Heer, F.T., de Beer, T.A.P., Rempfer, C., Bordoli, L., Lepore, R., Schwede, T. SWISS-MODEL: homology modelling of protein structures and complexes. Nucleic Acids Res. 46(W1), W296-W303 (2018).
- Bienert, S., Waterhouse, A., de Beer, T.A.P., Tauriello, G., Studer, G., Bordoli, L., Schwede, T. The SWISS-MODEL Repository - new features and functionality. Nucleic Acids Res. 45, D313-D319 (2017).
- Studer, G., Tauriello, G., Bienert, S., Biasini, M., Johner, N., Schwede, T. ProMod3 - A versatile homology modelling toolbox. PLOS Comp. Biol. 17(1), e1008667 (2021).
- Studer, G., Rempfer, C., Waterhouse, A.M., Gumienny, G., Haas, J., Schwede, T. QMEANDisCo - distance constraints applied on model quality estimation. Bioinformatics 36, 1765-1771 (2020).
- Bertoni, M., Kiefer, F., Biasini, M., Bordoli, L., Schwede, T. Modeling protein quaternary structure of homo- and hetero-oligomers beyond binary interactions by homology. Scientific Reports 7 (2017).

Results

The SWISS-MODEL template library (SMTL version 2022-12-28, PDB release 2022-12-23) was searched with for evolutionary related structures matching the target sequence in Table T1. For details on the template search, see Materials and Methods. Overall 6292 templates were found (Table T2).

Models

The following model was built (see Materials and Methods "Model Building"):

Model #01	File	Built with	Oligo-State	Ligands	GMQE	QMEANDisCo Global
	PDB	ProMod3 3.2.1	monomer	None	0.58	0.59 ± 0.05

Template	Seq Identity	Oligo-state	QSQE	Found by	Method	Resolution	Seq Similarity	Range	Coverage	Description
3qd2.1.A	31.42	monomer	0.00	HHblits	X-ray	2.81Å	0.35	4 - 305	0.94	Eukaryotic translation initiation factor 2-alpha kinase 3

The template contained no ligands.

```
Target    MSQLFQQQFQKPEKIGSGAEGSVFRVKHQFTGVSYAVKAIRIPDD---NEIYIQEAVLHSTFDSVNVVRFFNAWIERVPR
3qd2.1.A  ---RYLTDFEPIQCMGRGGFGVVFEAKNKVDDCNYAIKRIRLPNRELAREKVMREVKALAKLEHPGIVRYFNAWLETPPE

Target    NFAETLG--ILHR-------------------DDTMDNISAETLE-SFHETACYTVLFIQTEWFERGTLADHFVRRK---
3qd2.1.A  KWQEEMDEIWLKDESTDWPLSSPSPMDAPSVKIRRMDPFSTKNTVGQLQPSSPKVYLYIQMQLCRKENLKDWMNRRCSLE

Target    GFTRLENLKHLLQISEGLQYLHSQCVVHCDLKPRNIFMSDSGIMKIGDFGLSRKNRKRPHKLRGDAEGFASNSEAGDEGH
3qd2.1.A  DREHGVCLHIFIQIAEAVEFLHSKGLMHRDLKPSNIFFTMDDVVKVGDFGLVTAMDQDEEEQTV------LTPMPAYATH

Target    AIAAFTPLYCSPEQKRGDAATTASDIYSLGLIALEFYCVFTTQHERFCTLGEARQGVFPKEFADTYPVERALFQKMLSED
3qd2.1.A  TGQVGTKLYMSPEQIHGNNYSHKVDIFSLGLILFELLYSFSTQMERVRIITDVRNLKFPLLFTQKYPQEHMMVQDMLSPS

Target    ESCRPLMKDIVKALRQGIKEEEE
3qd2.1.A  PTERPEATDIIEN----------
```

Materials and Methods

Template Search

Template search with has been performed against the SWISS-MODEL template library (SMTL, last update: 2022-12-28, last included PDB release: 2022-12-23).

Model Building

Models are built based on the target-template alignment using ProMod3 (Studer et al.). Coordinates which are conserved between the target and the template are copied from the template to the model. Insertions and deletions are remodelled using a fragment library. Side chains are then rebuilt. Finally, the geometry of the resulting model is regularized by using a force field.

Model Quality Estimation

The global and per-residue model quality has been assessed using the QMEAN scoring function (Studer et al.).

Ligand Modelling

Ligands present in the template structure are transferred by homology to the model when the following criteria are met: (a) The ligands are annotated as biologically relevant in the template library, (b) the ligand is in contact with the model, (c) the ligand is not clashing with the protein, (d) the residues in contact with the ligand are conserved between the target and the template. If any of these four criteria is not satisfied, a certain ligand will not be included in the model. The model summary includes information on why and which ligand has not been included.

Oligomeric State Conservation

The quaternary structure annotation of the template is used to model the target sequence in its oligomeric form. The method (Bertoni et al.) is based on a supervised machine learning algorithm, Support Vector Machines (SVM), which combines interface conservation, structural clustering, and other template features to provide a quaternary structure quality estimate (QSQE). The QSQE score is a number between 0 and 1, reflecting the expected accuracy of the interchain contacts for a model built based a given alignment and template. Higher numbers indicate higher reliability. This complements the GMQE score which estimates the accuracy of the tertiary structure of the resulting model.

References

- **BLAST**
  Camacho, C., Coulouris, G., Avagyan, V., Ma, N., Papadopoulos, J., Bealer, K., Madden, T.L. BLAST+: architecture and applications. BMC Bioinformatics 10, 421-430 (2009).
- **HHblits**
  Steinegger, M., Meier, M., Mirdita, M., Vöhringer, H., Haunsberger, S. J., Söding, J. HH-suite3 for fast remote homology detection and deep protein annotation. BMC Bioinformatics 20, 473 (2019).

Table T1:

Primary amino acid sequence for which templates were searched and models were built.

```
MSQLFQQQFQKPEKIGSGAEGSVFRVKHQFTGVSYAVKAIRIPDDNEIYIQEAVLHSTFDSVNVVRFFNAWIERVPRNFAETLGILHRDDTMDNISAETL
ESFHETACYTVLFIQTEWFERGTLADHFVRRKGFTRLENLKHLLQISEGLQYLHSQCVVHCDLKPRNIFMSDSGIMKIGDFGLSRKNRKRPHKLRGDAEG
FASNSEAGDEGHAIAAFTPLYCSPEQKRGDAATTASDIYSLGLIALEFYCVFTTQHERFCTLGEARQGVFPKEFADTYPVERALFQKMLSEDESCRPLMK
DIVKALRQGIKEEEE
```

Table T2:

Template	Seq Identity	Oligo-state	QSQE	Found by	Method	Resolution	Seq Similarity	Coverage	Description
3qd2.1.A	31.42	monomer	-	HHblits	X-ray	2.81Å	0.35	0.94	Eukaryotic translation initiation factor 2-alpha kinase 3

Template	Seq Identity	Oligo-state	QSQE	Found by	Method	Resolution	Seq Similarity	Coverage	Description
6d3k.2.B	29.75	homo-dimer	0.08	HHblits	X-ray	2.60Å	0.34	0.89	Interferon-induced, double-stranded RNA-activated protein kinase
3qd2.1.A	33.79	monomer	-	BLAST	X-ray	2.81Å	0.36	0.92	Eukaryotic translation initiation factor 2-alpha kinase 3
7mf0.1.A	32.61	monomer	-	HHblits	X-ray	2.81Å	0.35	0.88	Eukaryotic translation initiation factor 2-alpha kinase 3,Eukaryotic translation initiation factor 2-alpha kinase 3
4x7k.1.A	32.61	monomer	-	HHblits	X-ray	1.80Å	0.35	0.88	Eukaryotic translation initiation factor 2-alpha kinase 3,Eukaryotic translation initiation factor 2-alpha kinase 3
4x7n.1.A	32.61	monomer	-	HHblits	X-ray	2.35Å	0.35	0.88	Eukaryotic translation initiation factor 2-alpha kinase 3,Eukaryotic translation initiation factor 2-alpha kinase 3
6d3k.1.B	29.75	homo-dimer	0.21	HHblits	X-ray	2.60Å	0.34	0.89	Interferon-induced, double-stranded RNA-activated protein kinase
6d3k.1.A	29.75	homo-dimer	0.21	HHblits	X-ray	2.60Å	0.34	0.89	Interferon-induced, double-stranded RNA-activated protein kinase
8eqd.1.A	32.61	monomer	-	HHblits	X-ray	2.92Å	0.35	0.88	Eukaryotic translation initiation factor 2-alpha kinase 3
4x7o.1.A	32.61	monomer	-	HHblits	X-ray	2.65Å	0.35	0.88	Eukaryotic translation initiation factor 2-alpha kinase 3,Eukaryotic translation initiation factor 2-alpha kinase 3
4x7n.1.A	34.07	monomer	-	BLAST	X-ray	2.35Å	0.36	0.86	Eukaryotic translation initiation factor 2-alpha kinase 3,Eukaryotic translation initiation factor 2-alpha kinase 3
6d3l.1.A	29.75	homo-dimer	0.03	HHblits	X-ray	3.10Å	0.34	0.89	Interferon-induced, double-stranded RNA-activated protein kinase
4x7k.1.A	34.07	monomer	-	BLAST	X-ray	1.80Å	0.36	0.86	Eukaryotic translation initiation factor 2-alpha kinase 3,Eukaryotic translation initiation factor 2-alpha kinase 3
1zy4.1.B	29.82	homo-dimer	0.34	HHblits	X-ray	1.95Å	0.34	0.87	Serine/threonine-protein kinase GCN2
3uiu.1.A	29.79	monomer	-	HHblits	X-ray	2.90Å	0.34	0.90	Interferon-induced, double-stranded RNA-activated protein kinase
1zxe.1.A	28.99	homo-dimer	0.30	HHblits	X-ray	2.60Å	0.34	0.88	Serine/threonine-protein kinase
4x7o.1.A	34.07	monomer	-	BLAST	X-ray	2.65Å	0.36	0.86	Eukaryotic translation initiation factor 2-alpha kinase 3,Eukaryotic translation initiation factor 2-alpha kinase 3
6n3o.1.A	25.27	monomer	-	HHblits	X-ray	2.40Å	0.32	0.89	eIF-2-alpha kinase GCN2
1zyc.1.B	28.99	homo-dimer	0.30	HHblits	X-ray	3.00Å	0.34	0.88	Serine/threonine-protein kinase GCN2
1zy4.1.A	29.82	homo-dimer	0.35	HHblits	X-ray	1.95Å	0.34	0.87	Serine/threonine-protein kinase GCN2

Template	Seq Identity	Oligo-state	QSQE	Found by	Method	Resolution	Seq Similarity	Coverage	Description
1zyc.2.B	28.99	homo-dimer	0.31	HHblits	X-ray	3.00Å	0.34	0.88	Serine/threonine-protein kinase GCN2
1zyd.1.A	28.99	homo-dimer	0.35	HHblits	X-ray	2.75Å	0.34	0.88	Serine/threonine-protein kinase GCN2
1zyc.2.A	28.99	homo-dimer	0.32	HHblits	X-ray	3.00Å	0.34	0.88	Serine/threonine-protein kinase GCN2
6d3k.2.B	35.29	homo-dimer	0.08	BLAST	X-ray	2.60Å	0.37	0.86	Interferon-induced, double-stranded RNA-activated protein kinase
6d3k.1.B	35.29	homo-dimer	0.19	BLAST	X-ray	2.60Å	0.37	0.86	Interferon-induced, double-stranded RNA-activated protein kinase
7qwk.1.A	26.91	monomer	-	HHblits	X-ray	2.30Å	0.33	0.96	eIF-2-alpha kinase GCN2
6d3k.1.A	35.29	homo-dimer	0.19	BLAST	X-ray	2.60Å	0.37	0.86	Interferon-induced, double-stranded RNA-activated protein kinase
1zyc.1.A	28.99	homo-dimer	0.30	HHblits	X-ray	3.00Å	0.34	0.88	Serine/threonine-protein kinase GCN2
7qwk.6.A	26.91	monomer	-	HHblits	X-ray	2.30Å	0.33	0.96	eIF-2-alpha kinase GCN2
6n3n.1.A	25.27	monomer	-	HHblits	X-ray	3.01Å	0.32	0.89	eIF-2-alpha kinase GCN2,eIF-2-alpha kinase GCN2
1zy4.1.B	32.39	homo-dimer	0.35	BLAST	X-ray	1.95Å	0.37	0.90	Serine/threonine-protein kinase GCN2
7qwk.5.A	26.91	monomer	-	HHblits	X-ray	2.30Å	0.33	0.96	eIF-2-alpha kinase GCN2
1zyc.1.B	32.04	homo-dimer	0.32	BLAST	X-ray	3.00Å	0.36	0.90	Serine/threonine-protein kinase GCN2
6n3l.1.A	25.71	monomer	-	HHblits	X-ray	2.61Å	0.32	0.89	eIF-2-alpha kinase GCN2,eIF-2-alpha kinase GCN2
1zyc.1.A	32.04	homo-dimer	0.33	BLAST	X-ray	3.00Å	0.36	0.90	Serine/threonine-protein kinase GCN2
1zyc.2.A	32.04	homo-dimer	0.35	BLAST	X-ray	3.00Å	0.36	0.90	Serine/threonine-protein kinase GCN2
1zyc.2.B	32.04	homo-dimer	0.34	BLAST	X-ray	3.00Å	0.36	0.90	Serine/threonine-protein kinase GCN2
1zy4.1.A	32.39	homo-dimer	0.37	BLAST	X-ray	1.95Å	0.37	0.90	Serine/threonine-protein kinase GCN2
1zyd.1.A	32.04	homo-dimer	0.38	BLAST	X-ray	2.75Å	0.36	0.90	Serine/threonine-protein kinase GCN2
1zxe.1.A	31.69	homo-dimer	0.33	BLAST	X-ray	2.60Å	0.36	0.90	Serine/threonine-protein kinase
2a1a.1.B	36.02	homo-dimer	0.10	BLAST	X-ray	2.80Å	0.38	0.83	Interferon-induced, double-stranded RNA-activated protein kinase
6d3l.1.A	35.29	homo-dimer	0.03	BLAST	X-ray	3.10Å	0.37	0.86	Interferon-induced, double-stranded RNA-activated protein kinase
3uiu.1.A	34.93	monomer	-	BLAST	X-ray	2.90Å	0.37	0.86	Interferon-induced, double-stranded RNA-activated protein kinase
7qq6.1.A	36.02	homo-dimer	0.22	BLAST	X-ray	2.80Å	0.38	0.83	eIF-2-alpha kinase GCN2
6n3o.1.A	34.09	monomer	-	BLAST	X-ray	2.40Å	0.38	0.84	eIF-2-alpha kinase GCN2
7qq6.2.A	36.02	monomer	-	BLAST	X-ray	2.80Å	0.38	0.83	eIF-2-alpha kinase GCN2
7qq6.2.B	36.02	monomer	-	BLAST	X-ray	2.80Å	0.38	0.83	eIF-2-alpha kinase GCN2

Template	Seq Identity	Oligo-state	QSQE	Found by	Method	Resolution	Seq Similarity	Coverage	Description
7qq6.1.B	36.02	homo-dimer	0.22	BLAST	X-ray	2.80Å	0.38	0.83	eIF-2-alpha kinase GCN2
6n3n.1.A	34.09	monomer	-	BLAST	X-ray	3.01Å	0.38	0.84	eIF-2-alpha kinase GCN2,eIF-2-alpha kinase GCN2
6n3l.1.A	34.09	monomer	-	BLAST	X-ray	2.61Å	0.38	0.84	eIF-2-alpha kinase GCN2,eIF-2-alpha kinase GCN2

The table above shows the top 50 filtered templates. A further 5,147 templates were found which were considered to be less suitable for modelling than the filtered list.
1a06.1.A, 1a9u.1.A, 1ad5.1.A, 1apm.1.A, 1aq1.1.A, 1b39.1.A, 1b6c.5.D, 1bi7.1.A, 1bi8.1.A, 1bkx.1.A, 1bl7.1.A, 1blx.1.A, 1bmk.1.A, 1buh.1.A, 1bx6.1.A, 1byg.1.A, 1cdk.1.A, 1ckj.1.A, 1ckj.1.B, 1ckp.1.A, 1cm8.1.A, 1cmk.1.A, 1csn.1.A, 1ctp.1.A, 1day.1.A, 1di8.1.A, 1di9.1.A, 1ds5.1.A, 1e7u.1.A, 1e8x.1.A, 1e8z.1.A, 1e90.1.A, 1e9h.1.A, 1eh4.1.A, 1eh4.2.A, 1f0q.1.A, 1f3m.1.D, 1f5q.1.A, 1f5q.2.A, 1fgi.1.A, 1fin.1.A, 1fin.2.A, 1fmk.1.A, 1fmo.1.A, 1fot.1.A, 1fpu.1.A, 1fq1.1.B, 1fvr.1.A, 1g3n.4.C, 1g5s.1.A, 1gag.2.C, 1gih.1.A, 1gij.1.A, 1gjo.1.A, 1gng.1.A, 1gol.1.A, 1gzn.1.A, 1gzo.1.A, 1h0w.1.A, 1h1p.2.A, 1h1q.2.A, 1h1w.1.A, 1h24.2.A, 1h27.1.A, 1h28.1.A, 1h4l.1.A, 1h8f.1.A, 1hcl.1.A, 1he8.1.A, 1how.1.A, 1i09.1.A, 1i44.1.A, 1ias.1.A, 1ias.1.B, 1iep.2.A, 1irk.1.A, 1j1b.1.A, 1j1b.1.B, 1j3h.1.A, 1j7i.1.A, 1j7u.1.A, 1j7u.1.B, 1j91.1.A, 1j91.2.A, 1jbp.1.A, 1jkt.1.A, 1jkt.2.A, 1jnk.1.A, 1jpa.1.A, 1jqh.2.A, 1jst.1.A, 1jst.1.C, 1jsu.1.A, 1jvp.1.A, 1jwh.1.A, 1jwh.1.B, 1k2p.1.A, 1k2p.1.B, 1k3a.1.A, 1k9a.1.A, 1k9a.2.A, 1k9a.3.A, 1k9a.5.A, 1k9a.6.A, 1koa.1.A, 1ksw.1.A, 1kv1.1.A, 1kwp.3.B, 1kwp.3.C, 1l8t.1.A, 1lp4.1.A, 1luf.1.A, 1m14.1.A, 1m17.1.A, 1m2p.1.A, 1m2q.1.A, 1m2r.1.A, 1m52.1.A, 1m7n.1.A, 1mp8.1.A, 1mq4.1.A, 1mqb.1.A, 1mqb.2.A, 1mru.1.A, 1mru.2.A, 1mrv.1.A, 1muo.1.A, 1na7.1.A, 1nd4.1.A, 1nvq.1.A, 1nw1.1.A, 1nxk.1.A, 1ny3.1.A, 1o6k.1.A, 1o6l.1.A, 1o6y.1.A, 1o9u.1.A, 1oi9.2.A, 1oit.1.A, 1oiu.1.A, 1oiy.2.A, 1okv.1.A, 1okv.2.A, 1oky.1.A, 1ol5.1.A, 1ol6.1.A, 1ol7.1.A, 1omw.1.A, 1opj.1.A, 1opk.1.A, 1opl.1.A, 1opl.2.A, 1ouk.1.A, 1oz1.1.A, 1p14.1.A, 1p2a.1.A, 1p4f.1.A, 1p4o.1.A, 1p5e.1.A, 1pf8.1.A, 1phk.1.A, 1pjk.1.A, 1pkg.1.A, 1pkg.2.A, 1pme.1.A, 1pmu.1.A, 1pmv.1.A, 1pxo.1.A, 1py5.1.A, 1pyx.1.A, 1pyx.1.B, 1q24.1.A, 1q3d.1.B, 1q3w.1.A, 1q5k.1.A, 1q5k.1.B, 1q8w.1.A, 1q8y.1.A, 1q8y.2.A, 1qcf.1.A, 1ql6.1.A, 1qmz.1.A, 1qpd.1.A, 1qpj.1.A, 1r0e.1.A, 1r0p.1.A, 1r39.1.A, 1r3c.1.A, 1r78.1.A, 1rdq.1.A, 1re8.1.A, 1rek.1.A, 1rjb.1.A, 1rqq.1.A, 1rw8.1.A, 1s9i.1.A, 1s9i.1.B, 1sm2.1.A, 1sm2.2.A, 1smh.1.A, 1snu.2.A, 1stc.1.A, 1syk.1.A, 1szm.1.A, 1szm.2.A, 1t45.1.A, 1t46.1.A, 1tki.1.A, 1tqp.1.A, 1tvo.1.A, 1u4d.1.A, 1u4d.2.A, 1u54.1.A, 1u54.2.A, 1u59.1.A, 1u5q.1.A, 1u5q.2.A, 1u5r.1.A, 1u5r.2.A, 1ua2.1.A, 1ua2.2.A, 1ua2.3.A, 1ua2.4.A, 1ukh.1.A, 1uki.1.A, 1ung.1.A, 1ung.2.A, 1unh.1.A, 1unl.2.A, 1uu7.1.A, 1uu9.1.A, 1uv5.1.A, 1uwh.1.A, 1uwj.1.A, 1v0b.1.A, 1v0o.1.A, 1v0o.2.A, 1v0p.1.A, 1v0p.2.A, 1v1k.1.A, 1vjy.1.A, 1vr2.1.A, 1vyw.1.A, 1vyw.2.A, 1vzo.1.A, 1w7h.1.A, 1w82.1.A, 1w98.1.A, 1wak.1.A, 1wbo.1.A, 1wbv.1.A, 1wbw.1.A, 1wmk.1.A, 1wvw.1.A, 1wvx.1.A, 1wvy.1.A, 1wzy.1.A, 1x8b.1.A, 1xh4.1.A, 1xh6.1.A, 1xh9.1.A, 1xjd.1.A, 1xkk.1.A, 1xo2.1.B, 1xr1.1.A, 1xws.1.A, 1y57.1.A, 1y6a.1.A, 1y8g.1.A, 1y8y.1.A, 1y91.1.A, 1ydt.1.A, 1yhv.1.A, 1yhw.1.A, 1yi3.1.A, 1yi4.1.A, 1yi6.1.A, 1yi6.2.A, 1ym7.2.A, 1yoj.1.A, 1yol.1.A, 1yol.2.A, 1yom.2.A, 1yqj.1.A, 1yrp.1.A, 1yvj.1.A, 1yw2.1.A, 1ywn.1.A, 1ywr.1.A, 1yxs.1.A, 1yxu.1.A, 1z57.1.A, 1z9x.1.A, 1zlt.1.A, 1zmu.1.A, 1zmv.1.A, 1zoe.1.A, 1zog.1.A, 1zp9.3.A, 1zrz.1.A, 1zth.1.A, 1zth.4.A, 1zws.1.A, 1zxe.1.B, 1zxe.3.A, 1zxe.3.B, 1zyl.1.A, 1zys.1.A, 1zz2.1.A, 2a0c.1.A, 2a19.1.B, 2a19.1.C, 2a1a.1.B, 2a27.1.A, 2a2a.1.A, 2a4z.1.A, 2a5u.1.A, 2ac3.1.A, 2ac5.1.A, 2acx.1.A, 2auh.1.A, 2ayp.1.A, 2b0q.1.A, 2b1p.1.A, 2b4s.1.D, 2b52.1.A, 2b53.1.A, 2b54.1.A, 2b9h.1.A, 2baj.1.A, 2bak.1.A, 2bal.1.A, 2baq.1.A, 2bcj.1.A, 2bdj.1.A, 2bdw.1.A, 2bdw.1.B, 2bfy.1.A, 2bfy.2.A, 2biy.1.A, 2bkk.1.A, 2bkk.2.A, 2bmc.1.A, 2brb.1.A, 2bts.1.A, 2bva.1.A, 2bva.2.A, 2c0o.1.A, 2c30.1.A, 2c3i.1.A, 2c47.1.A, 2c5o.1.A, 2c6d.1.A, 2c6e.1.A, 2c6e.2.A, 2cch.1.A, 2cdz.1.A, 2cgv.1.A, 2chl.1.A, 2chw.1.A, 2chx.1.A, 2cjm.2.A, 2cko.1.A, 2ckp.1.A, 2ckp.1.B, 2ckq.1.A, 2clq.1.A, 2cmw.1.A, 2cn8.1.A, 2cpk.1.A, 2csn.1.A, 2dq7.1.A, 2dwb.1.A, 2dyl.1.A, 2e2b.1.A, 2e9p.1.A, 2e9v.1.A, 2eb2.1.A, 2eb3.1.A, 2erk.1.A, 2erz.1.A, 2etm.1.A, 2etm.2.A, 2eu9.1.A, 2euf.1.B, 2eva.1.A, 2ewa.1.A, 2exc.1.A, 2exe.1.A, 2exm.1.A, 2f2c.1.B, 2f2u.1.A, 2f2u.2.A, 2f49.1.A, 2f49.2.A, 2f4j.1.A, 2f57.1.A, 2f57.2.A, 2f9g.1.A, 2fb8.1.A, 2fh9.1.A, 2fo0.1.A, 2fq3.1.A, 2fsl.1.A, 2fso.1.A, 2fst.1.A, 2fum.1.A, 2fum.3.A, 2fvd.1.A, 2fys.1.A, 2fys.2.A, 2g01.1.A, 2g01.2.A, 2g15.1.A, 2g1t.1.A, 2g2f.1.A, 2g2f.2.A, 2g2h.1.A, 2g2h.2.A, 2g2i.1.A, 2g2i.2.A, 2gcd.1.A, 2gcd.2.A, 2gdo.1.A, 2gfc.1.A, 2gfs.1.A, 2ghl.1.A, 2ghm.1.A, 2gmx.3.A, 2gmx.3.C, 2gnf.1.A, 2gnj.1.A, 2gph.1.A, 2gqg.1.A, 2gqg.2.A, 2gs2.1.A, 2gs7.1.A, 2gtm.1.A, 2gu8.1.A, 2h34.1.A, 2h34.2.A, 2h6d.1.A, 2h8h.1.A, 2h96.2.A, 2h9v.1.A, 2hak.1.A, 2hak.2.A, 2hak.3.A, 2hak.6.A, 2hak.7.A, 2hck.1.B, 2hel.1.A, 2hen.1.A, 2hiw.1.A, 2hiw.2.A, 2hk5.1.A, 2hw6.1.A, 2hw6.2.A, 2hw7.1.A, 2hwo.1.A, 2hxq.1.A, 2hyy.1.A, 2hz0.1.A, 2hz0.2.A, 2hz4.1.A, 2hz4.2.A, 2hz4.3.A, 2hzi.1.A, 2hzi.2.A, 2hzn.1.A, 2i0e.1.A, 2i0v.1.A, 2i0y.1.A, 2i1m.1.A, 2i40.1.A, 2i40.2.A, 2i7q.1.A, 2ig7.1.A, 2ijm.1.A, 2in6.1.A, 2itn.1.A, 2itp.1.A, 2itt.1.A, 2ity.1.A, 2itz.1.A, 2ivs.1.B, 2ivv.1.A, 2iw8.1.A, 2iw9.1.A, 2iwi.1.A, 2izr.1.A, 2izu.1.A, 2j0i.1.A, 2j0j.1.A, 2j0k.1.A, 2j0k.2.A, 2j0l.1.A, 2j0m.1.B, 2j2i.1.A, 2j4z.1.A, 2j4z.2.A, 2j50.1.A, 2j51.1.A, 2j5e.1.A, 2j5f.1.A, 2j6m.1.A, 2j90.1.A, 2j90.1.B, 2jam.1.A, 2jav.1.A, 2jbo.1.A, 2jbp.10.A, 2jbp.11.A, 2jbp.12.A, 2jbp.5.A, 2jc6.1.A, 2jds.1.A, 2jed.1.A, 2jed.2.A, 2jgz.1.A, 2jii.1.A, 2jit.1.A, 2jit.2.A, 2jiu.1.A, 2jiu.2.A, 2jiv.1.A, 2jiv.2.A, 2jkk.1.A, 2jkm.1.A, 2jko.1.A, 2jkq.1.A, 2kty.1.A, 2kul.1.A, 2lav.1.A, 2lgc.1.A, 2no3.1.A, 2no3.3.C, 2np8.1.A, 2npq.1.A, 2nru.1.A, 2nru.2.A, 2nru.3.A, 2nru.4.A, 2nry.1.A, 2nry.2.A, 2nry.3.A, 2nry.4.A, 2o5k.1.A, 2o8y.1.A, 2ofu.1.A, 2ofv.1.A, 2ofv.1.B, 2og8.1.A, 2ogv.1.A, 2oh4.1.A, 2oib.1.A, 2oib.2.A, 2oib.3.A, 2oib.4.A, 2oic.1.A, 2oic.3.A, 2oic.4.A, 2oid.1.A, 2oid.2.A, 2oj9.1.A, 2ojf.1.A, 2ojg.1.A, 2ojj.1.A, 2ok1.1.A, 2okr.1.A, 2onl.1.B, 2onl.2.B, 2ou7.2.B, 2ow3.1.A, 2oxy.1.A, 2oza.1.A, 2oza.1.B, 2ozo.1.A, 2p2h.1.A, 2p2i.1.A, 2p4i.1.A, 2p4i.2.A, 2pe2.1.A, 2phk.1.A, 2pl0.1.A, 2pmi.1.A, 2pml.1.A, 2pmn.1.A, 2ppq.1.A, 2psq.1.A, 2ptk.1.A, 2pup.1.A, 2puu.1.A, 2pvf.1.A, 2pvh.1.A, 2pvr.1.A, 2pwl.1.A, 2py3.1.A, 2pyw.1.A, 2pz5.1.A, 2pzi.1.A, 2pzi.2.A, 2pzp.1.A, 2pzr.1.A, 2pzy.1.A, 2pzy.2.A, 2pzy.3.A, 2pzy.4.A, 2q0b.1.A, 2q0n.1.A, 2q83.1.A, 2qd9.1.A, 2qg5.1.A, 2qg5.2.A, 2qg7.1.A, 2qg7.1.B, 2qg7.2.A, 2qg7.2.B, 2qi8.1.A, 2qkr.1.A, 2qkw.1.B, 2qlq.1.A, 2qlq.2.A, 2qlu.1.A, 2qnj.1.A, 2qnj.1.B, 2qo2.1.A, 2qob.1.A, 2qod.1.A, 2qof.1.A, 2qoh.1.A, 2qoi.1.A, 2qok.1.A, 2qol.1.A, 2qon.1.A,

2qoo.1.A, 2qu5.1.A, 2qu6.1.A, 2qur.1.A, 2qvs.1.A, 2r0i.1.A, 2r0u.1.A, 2r2p.1.A, 2r3n.1.A, 2r4b.1.A, 2r7b.1.A, 2r7i.1.A, 2r9s.1.A, 2rd0.1.A, 2rei.1.A, 2rf9.1.A, 2rf9.2.A, 2rfd.1.A, 2rfd.2.A, 2rfe.2.A, 2rfe.4.A, 2rfn.1.A, 2rg5.1.A, 2rg6.1.A, 2rgp.1.A, 2rku.1.A, 2rl5.1.A, 2rsv.1.A, 2src.1.A, 2uzb.2.A, 2uzd.1.A, 2uzl.1.A, 2uzv.1.B, 2v55.1.A, 2v5q.1.A, 2v5q.2.A, 2v62.1.A, 2v7a.1.A, 2vd5.1.A, 2vn9.1.A, 2vo6.1.A, 2vrx.1.A, 2vrx.2.A, 2vtn.1.A, 2vtt.1.A, 2vuw.1.A, 2vwb.1.A, 2vwb.2.A, 2vwi.1.A, 2vwi.1.B, 2vwi.1.C, 2vwi.1.D, 2vwx.1.A, 2vx3.2.A, 2vz6.1.A, 2w1g.1.A, 2w1i.1.A, 2w1z.1.A, 2w4j.1.A, 2w4k.1.A, 2w4o.1.A, 2w5a.1.A, 2w5h.1.A, 2w96.1.B, 2w99.1.B, 2w9f.1.B, 2w9z.1.B, 2waj.1.A, 2wb8.1.A, 2wd1.1.A, 2wei.1.A, 2wel.1.A, 2wgj.1.A, 2wma.1.A, 2wma.2.A, 2wmb.1.A, 2wmb.2.A, 2wnt.1.A, 2wo6.1.A, 2wo6.2.A, 2wqb.1.A, 2wqe.1.A, 2wqn.1.A, 2wtk.1.B, 2wtk.1.C, 2wtk.2.C, 2wtv.1.A, 2wtv.2.A, 2wtw.1.A, 2wu6.1.A, 2wxl.1.A, 2wzj.1.A, 2x0g.1.A, 2x39.1.A, 2x4f.1.A, 2x4z.1.A, 2x6e.1.A, 2x6f.1.A, 2x6f.1.B, 2x6j.1.B, 2x7f.1.A, 2x7o.5.A, 2x8e.1.A, 2x8i.1.A, 2x9e.1.A, 2xa4.1.A, 2xba.1.A, 2xch.1.A, 2xck.1.A, 2xik.1.A, 2xj0.1.A, 2xk9.1.A, 2xke.1.A, 2xm8.1.A, 2xm9.1.A, 2xmy.1.A, 2xnb.1.A, 2xne.1.A, 2xng.1.A, 2xno.1.A, 2xru.1.A, 2xrw.1.A, 2xs0.1.A, 2xuu.1.A, 2xvd.1.A, 2xyn.3.A, 2xyu.1.A, 2xzs.1.A, 2y0a.1.A, 2y3a.1.A, 2y4i.1.A, 2y4i.1.B, 2y4p.1.A, 2y4p.1.B, 2y6o.1.A, 2y7j.1.A, 2y7j.1.B, 2y8o.1.A, 2y9q.1.A, 2ya9.1.A, 2yak.1.A, 2ycf.1.A, 2ycr.1.A, 2ycs.1.A, 2yex.1.A, 2yfx.1.A, 2yhv.1.A, 2yiw.1.A, 2yix.1.A, 2yiy.1.A, 2yjr.1.A, 2yjs.1.A, 2yn8.1.A, 2ywp.1.A, 2yza.1.A, 2z2w.1.A, 2z60.1.A, 2z7l.1.A, 2z7s.1.A, 2z8c.1.A, 2zdt.1.A, 2zdu.1.A, 2zjw.1.A, 2zm3.5.G, 2zm3.5.H, 2zmc.1.A, 2zmd.1.A, 2zoq.1.A, 2zoq.2.A, 2zv2.1.A, 2zv9.1.A, 3a2c.1.A, 3a2c.1.D, 3a2c.1.G, 3a2c.1.L, 3a4o.1.A, 3a4p.1.A, 3a60.1.A, 3a60.1.B, 3a61.1.A, 3a62.1.A, 3a7f.1.A, 3a7h.3.A, 3a7h.3.B, 3a7i.1.A, 3a8w.1.A, 3a8x.1.A, 3a99.1.A, 3ack.1.A, 3ag9.1.A, 3ag9.3.B, 3agl.1.A, 3agm.1.A, 3aln.1.A, 3aln.2.A, 3aln.3.A, 3alo.1.B, 3ama.1.A, 3amy.1.A, 3anq.1.A, 3aox.1.A, 3apf.1.A, 3aqv.1.A, 3at2.1.A, 3at4.1.A, 3ats.1.A, 3b2t.1.A, 3b2w.1.A, 3b8q.2.A, 3b8r.1.A, 3b8r.2.A, 3bbt.1.A, 3bce.1.A, 3be2.1.A, 3bea.1.A, 3beg.1.A, 3bgq.1.A, 3bhh.1.A, 3bhh.3.A, 3bht.1.A, 3bht.2.A, 3bhy.1.A, 3bi6.1.A, 3bkb.1.A, 3blq.1.A, 3blr.1.A, 3bpr.1.A, 3bqc.1.A, 3bqr.1.A, 3brb.2.A, 3bu3.1.A, 3bv2.1.A, 3bv3.1.A, 3bx5.1.A, 3bym.1.A, 3byo.1.A, 3bys.1.A, 3byv.1.A, 3bz3.1.A, 3c0g.1.A, 3c0i.1.A, 3c1x.1.A, 3c4c.1.A, 3c4c.2.A, 3c4d.1.A, 3c4d.2.A, 3c4e.1.A, 3c4f.1.A, 3c4x.1.A, 3c4y.1.A, 3c4y.1.B, 3c4z.1.A, 3c50.1.B, 3c51.1.A, 3c51.1.B, 3c5i.1.A, 3c5u.1.A, 3c7q.1.A, 3c9w.1.A, 3cc6.1.A, 3cd3.1.A, 3cd8.1.A, 3ce3.1.A, 3cik.1.A, 3cjf.1.A, 3cjg.1.A, 3ckw.1.A, 3cly.1.A, 3coh.1.A, 3coi.1.A, 3cok.1.A, 3com.1.A, 3com.2.A, 3cqu.1.A, 3cs9.1.A, 3cs9.4.A, 3csf.1.A, 3csv.1.A, 3cth.1.A, 3ctj.1.A, 3ctq.1.A, 3cxw.1.A, 3d0e.1.A, 3d14.1.A, 3d2k.1.A, 3d5v.1.A, 3d5w.1.A, 3d5x.1.A, 3d7t.1.A, 3d7t.2.B, 3d7u.1.A, 3d7u.1.B, 3d7z.1.A, 3d94.1.A, 3d9v.1.A, 3d9v.1.B, 3dae.1.A, 3daj.1.A, 3dak.1.A, 3dak.1.C, 3dbq.1.A, 3dbs.1.A, 3dcv.1.A, 3ddp.1.A, 3ddq.1.A, 3dfa.1.A, 3dfc.1.A, 3dj5.1.A, 3dk3.1.A, 3dk3.2.A, 3dk6.1.A, 3dk6.2.A, 3dk7.1.A, 3dk7.2.A, 3dkc.1.A, 3dkf.1.A, 3dkg.1.A, 3dko.1.A, 3dls.1.A, 3dnd.1.A, 3dpd.1.A, 3dqw.1.A, 3dqw.3.A, 3dqx.1.A, 3dqx.2.A, 3ds6.1.A, 3ds6.2.A, 3ds6.3.A, 3ds6.4.A, 3dt1.1.A, 3dtc.1.A, 3du8.2.A, 3dv3.1.A, 3dxn.1.A, 3dxp.1.A, 3dxq.1.A, 3dy7.1.A, 3dzo.1.A, 3dzq.1.A, 3e3b.1.A, 3e3p.1.A, 3e5a.1.A, 3e64.1.A, 3e7o.1.A, 3e7o.1.B, 3e8c.2.A, 3e8n.1.A, 3e92.1.A, 3e93.1.A, 3eb0.1.A, 3efl.2.A, 3ekk.1.A, 3el7.1.A, 3el8.1.A, 3elj.1.A, 3emg.1.A, 3en9.1.A, 3en9.2.A, 3ene.1.A, 3enh.1.A, 3enm.1.A, 3enm.1.B, 3enm.2.A, 3eqb.1.A, 3eqc.1.A, 3eqp.1.A, 3eqr.1.A, 3erk.1.A, 3eta.1.A, 3eyg.1.A, 3ezv.1.A, 3f2a.1.A, 3f2n.1.A, 3f3u.2.A, 3f3w.1.A, 3f3z.1.A, 3f5g.1.A, 3f5p.12.A, 3f5u.1.A, 3f61.1.A, 3f66.1.A, 3f66.2.A, 3f69.1.A, 3f6x.1.A, 3f6x.2.A, 3f7w.1.A, 3f7z.1.A, 3f88.1.A, 3f9n.1.A, 3faa.1.A, 3faa.3.A, 3fc1.1.A, 3fe3.1.A, 3fe3.1.B, 3feg.1.A, 3fhi.1.A, 3fi2.1.A, 3fi3.1.A, 3fi4.1.A, 3fi8.1.A, 3fkl.1.A, 3fko.1.A, 3fme.1.A, 3fpm.1.A, 3fsf.1.A, 3fv8.1.A, 3fwq.1.A, 3fwq.2.A, 3fxw.1.A, 3fxx.1.A, 3fyk.1.B, 3fzo.1.A, 3fzr.1.A, 3fzt.1.A, 3g0e.1.A, 3g0f.1.A, 3g0f.1.B, 3g2f.1.A, 3g2f.2.A, 3g33.1.A, 3g33.1.C, 3g51.1.A, 3g6g.1.A, 3g6h.1.A, 3g6h.2.A, 3g90.1.A, 3gb2.1.A, 3gc0.1.A, 3gc7.1.A, 3gc8.1.A, 3gc8.2.A, 3gc9.1.A, 3gc9.2.A, 3gcv.1.A, 3gen.1.A, 3geq.1.A, 3gfe.1.A, 3ggf.1.A, 3gi3.1.A, 3gni.1.B, 3gok.1.A, 3gop.1.A, 3gp0.1.A, 3gqi.1.A, 3gql.1.A, 3gql.2.A, 3gql.3.A, 3gt8.1.A, 3gt8.1.B, 3gt8.2.A, 3gt8.2.B, 3gu4.1.A, 3gu8.1.A, 3gub.1.A, 3gvu.2.B, 3h0y.2.A, 3h0z.1.A, 3h0z.2.A, 3h0z.3.A, 3h10.1.A, 3h10.3.A, 3h30.1.A, 3h3c.1.A, 3h4j.1.A, 3h4j.2.A, 3h9f.1.A, 3h9o.1.A, 3h9r.1.A, 3ha6.1.A, 3ha8.1.A, 3ham.1.A, 3ham.1.B, 3hav.1.A, 3hav.2.A, 3hav.3.A, 3hdn.1.A, 3hec.1.A, 3heg.1.A, 3hgk.1.A, 3hhm.1.A, 3hiz.1.A, 3hko.1.A, 3hll.1.A, 3hmi.1.A, 3hmn.1.A, 3hng.1.A, 3hp2.1.A, 3hrb.1.A, 3hub.1.A, 3huc.1.A, 3hv3.1.A, 3hv4.1.A, 3hv5.2.A, 3hvc.1.A, 3hx4.1.A, 3hyh.1.A, 3hyh.2.A, 3hzt.1.A, 3i0o.1.A, 3i0q.1.A, 3i1a.1.A, 3i1a.2.A, 3i4b.1.A, 3i4b.1.B, 3i5z.1.A, 3i6u.1.A, 3i6u.1.B, 3i6w.1.A, 3i6w.1.B, 3i6w.2.A, 3i6w.2.B, 3i6w.3.A, 3i6w.3.B, 3i79.1.A, 3i81.1.A, 3idb.1.A, 3idp.1.A, 3iec.1.A, 3igo.1.A, 3ihy.1.A, 3ihy.3.A, 3ihy.4.A, 3ii5.1.A, 3ik3.1.A, 3ik3.2.A, 3ika.1.A, 3iph.1.A, 3is5.1.A, 3is5.1.F, 3iw4.1.A, 3iw4.2.A, 3iw7.1.A, 3j4r.1.D, 3jbz.1.A, 3jr1.1.A, 3js2.1.A, 3juh.1.A, 3jvr.1.A, 3jy0.1.A, 3jy9.1.A, 3jya.1.A, 3k3i.1.A, 3k3j.1.A, 3k54.1.A, 3k5u.1.A, 3k5v.2.A, 3ka0.1.A, 3kb7.1.A, 3kc3.1.A, 3kc3.1.B, 3kc3.1.C, 3kc3.2.A, 3kc3.2.C, 3kc3.3.B, 3kc3.3.C, 3kc3.4.A, 3kc3.4.B, 3kc3.4.C, 3kcf.3.A, 3kcf.4.A, 3kcf.5.A, 3kck.1.A, 3kex.1.A, 3kf4.1.A, 3kfa.2.A, 3kk8.1.A, 3kk9.1.A, 3kl8.1.A, 3kmm.1.A, 3kn6.1.A, 3kn6.2.A, 3kq7.1.A, 3krj.1.A, 3krl.1.A, 3krr.1.A, 3krx.1.A, 3ku2.1.A, 3kul.1.A, 3kvw.1.A, 3kvx.1.A, 3kxx.1.A, 3kxx.3.A, 3kxx.4.A, 3kxz.1.A, 3l54.1.A, 3l8p.1.A, 3l8v.1.A, 3l9l.1.A, 3l9m.1.A, 3lcd.1.A, 3lco.1.A, 3lfb.1.A, 3lff.1.A, 3lfs.1.A, 3lij.1.A, 3lj0.1.A, 3lj1.1.A, 3lj3.1.A, 3llt.1.A, 3lm0.1.A, 3lmg.1.A, 3lok.1.A, 3lpb.1.A, 3lq3.1.A, 3lq8.1.A, 3ls8.1.A, 3lvp.1.A, 3lvp.2.A, 3lvp.3.A, 3lvp.4.A, 3lw0.2.A, 3lxk.1.A, 3lxn.1.A, 3lxp.1.A, 3lzb.1.A, 3m1s.1.A, 3m2w.1.A, 3ma6.1.A, 3ma6.2.A, 3mbl.1.A, 3mdy.1.A, 3mes.1.A, 3mfr.1.A, 3mgy.1.A, 3mh0.1.A, 3mh1.1.A, 3mh2.1.A, 3mh3.1.A, 3mia.1.A, 3mj1.1.A, 3mjw.1.A, 3mn3.1.A, 3mpa.1.A, 3mpt.1.A, 3mtf.2.A, 3mtl.1.A, 3mv5.1.A, 3mvj.1.A, 3mvj.2.A, 3mvj.3.A, 3mvl.1.A, 3mvl.2.A, 3mvm.1.A, 3mvm.2.A, 3mwu.1.A, 3my0.1.A, 3my1.1.A, 3myg.1.A, 3n4t.1.A, 3n4u.1.A, 3n4v.1.A, 3n9x.1.A, 3nax.1.A, 3nay.1.A, 3ncz.1.A, 3ncz.1.B, 3ncz.2.A, 3ncz.2.B, 3ndm.1.A, 3new.1.A, 3nga.1.A, 3nie.1.A, 3niz.1.A, 3nlb.1.A, 3nnu.1.A, 3nnx.1.A, 3npc.1.A, 3nr9.1.A, 3nrm.1.A, 3nsz.1.A, 3nun.1.A, 3nus.1.A, 3nw5.1.A, 3nw6.1.A, 3nw7.1.A, 3nx8.1.A, 3nyn.1.A, 3nyx.1.A, 3nz0.1.A, 3nzs.1.A, 3o17.1.A, 3o17.2.A, 3o23.1.A, 3o2m.1.A, 3o50.1.A, 3o50.2.A, 3o51.1.A, 3o71.1.A, 3o7l.1.A, 3o7l.1.B, 3o8p.1.A, 3o8t.1.A, 3o8u.1.A, 3o96.1.A, 3oaw.1.A, 3obg.1.A, 3obj.1.A, 3oc1.1.A, 3ocb.1.A, 3ocb.2.A, 3ocg.1.A, 3ocs.1.A, 3oct.1.A, 3od6.1.A, 3ody.1.A, 3odz.1.A, 3oef.1.A, 3oez.1.A, 3oez.2.A, 3ofm.1.A, 3og7.1.A, 3og7.2.A, 3oht.1.A, 3omv.1.A, 3op5.2.A, 3op5.3.A, 3ori.2.A, 3orl.1.A, 3orm.1.A, 3orn.2.A, 3oro.1.A, 3ort.1.A, 3orx.1.A, 3orx.5.A, 3orz.1.A, 3os3.1.A, 3ot3.1.A, 3otu.1.A, 3otv.1.A, 3otv.3.A, 3otv.4.A, 3ouk.1.A, 3oun.1.B, 3ovv.1.A, 3owj.1.A, 3oxi.1.A, 3oxz.1.A, 3oy1.1.A, 3oy3.1.A, 3oz6.1.A, 3oz6.1.B, 3p08.1.A, 3p1a.1.A, 3p23.1.A, 3p23.1.B, 3p4k.1.A, 3p7a.1.A, 3p7c.1.A, 3p86.1.A, 3p86.2.A, 3p9j.1.A, 3pe2.1.A, 3pfq.1.A, 3pg1.1.A, 3pg3.1.A, 3pix.1.A, 3piy.1.A, 3pj3.1.A, 3pj8.1.A, 3pjc.1.A, 3pls.1.A, 3poz.1.A, 3pp0.1.A, 3pp0.2.A, 3ppj.1.A, 3ppz.1.A, 3psc.1.A, 3ptg.1.A, 3pvb.1.A, 3pvg.1.A, 3pwy.1.A, 3pxf.1.A, 3pxk.2.A, 3pxk.3.A, 3pxq.1.A,

3pxr.1.A, 3pxy.1.A, 3py0.1.A, 3py1.1.A, 3py3.1.A, 3pyy.1.A, 3pyy.2.A, 3pze.1.A, 3q04.1.A, 3q2j.1.A, 3q2j.1.B, 3q2m.2.A, 3q32.1.A, 3q32.2.A, 3q4c.1.A, 3q4t.1.A, 3q4u.1.A, 3q4u.1.B, 3q4u.2.A, 3q4u.2.B, 3q4z.1.A, 3q4z.1.B, 3q53.1.A, 3q5i.1.A, 3q5z.1.A, 3q60.1.A, 3q6u.1.A, 3q6w.1.A, 3q96.1.A, 3q9w.1.A, 3q9x.2.A, 3q9y.1.A, 3q9z.1.A, 3qa0.1.A, 3qa8.1.A, 3qa8.1.B, 3qa8.1.D, 3qa8.2.B, 3qal.1.A, 3qam.1.A, 3qbn.1.A, 3qc4.1.A, 3qc4.2.A, 3qc9.1.A, 3qf9.1.A, 3qfv.1.A, 3qgw.1.A, 3qgw.2.A, 3qgy.2.A, 3qhr.1.A, 3qkk.1.A, 3qkl.1.A, 3qlf.1.A, 3qqf.1.A, 3qqh.1.A, 3qql.1.A, 3qqu.1.A, 3qqu.3.A, 3qrj.1.A, 3qrk.1.A, 3qrt.1.A, 3qru.1.A, 3qti.1.A, 3qtu.1.A, 3qtw.1.A, 3qtx.1.A, 3qup.1.A, 3qwj.1.A, 3qxp.1.A, 3qyw.1.A, 3qzi.1.A, 3r21.1.A, 3r22.1.A, 3r2b.1.A, 3r2y.1.A, 3r63.1.A, 3r78.1.A, 3r78.2.A, 3r83.1.A, 3r8m.1.A, 3rah.1.A, 3raw.1.A, 3rcd.1.A, 3rcd.2.A, 3re4.1.A, 3rep.1.A, 3rgf.1.A, 3rhk.1.A, 3rhx.1.A, 3ri1.1.A, 3rin.1.A, 3rkb.1.A, 3rm6.1.A, 3rni.1.A, 3rny.1.A, 3rp0.1.A, 3rp0.2.A, 3rp9.1.A, 3rps.1.A, 3rps.2.A, 3rtp.1.A, 3rvg.1.A, 3rwp.1.A, 3rwq.1.A, 3rzb.1.A, 3rzf.1.A, 3s0o.1.A, 3s3i.1.A, 3s4q.1.A, 3s95.1.A, 3sa0.1.A, 3say.1.A, 3sd0.1.A, 3sd0.1.B, 3sd5.1.A, 3sdj.1.A, 3sdm.1.A, 3sg8.1.A, 3sg9.1.A, 3sgc.1.A, 3she.1.A, 3sls.1.A, 3soa.1.A, 3soc.1.A, 3soc.2.A, 3sqq.1.A, 3srv.1.A, 3sv0.1.A, 3svv.1.A, 3sxr.1.A, 3sxs.1.A, 3t3u.1.A, 3t8m.1.A, 3t8o.1.A, 3t9i.1.A, 3t9t.1.A, 3tac.1.A, 3tcp.1.A, 3tcp.2.A, 3tdv.1.A, 3tdw.1.A, 3tei.1.A, 3tg1.1.A, 3thb.1.A, 3ti1.1.A, 3tiz.1.A, 3tjd.1.A, 3tjd.2.A, 3tku.1.A, 3tl5.1.A, 3tl8.1.A, 3tl8.2.A, 3tl8.3.A, 3tm0.1.A, 3tn8.1.A, 3tni.1.A, 3tnp.2.A, 3tnq.1.A, 3tnw.1.A, 3tt0.1.A, 3tt0.2.A, 3ttj.1.A, 3tub.1.A, 3tuc.1.A, 3tud.1.A, 3tv7.1.A, 3tv7.2.A, 3twj.1.A, 3twj.1.B, 3twj.2.A, 3twj.2.B, 3txo.1.A, 3tyk.1.A, 3tz7.2.A, 3tz9.1.A, 3tz9.2.A, 3tzm.1.A, 3u4u.1.A, 3u4w.1.A, 3u51.1.A, 3u6h.1.A, 3u6j.1.A, 3u9c.1.A, 3u9n.1.A, 3ubd.1.A, 3uc3.1.A, 3uc4.1.A, 3uc4.2.A, 3udb.1.A, 3udb.2.A, 3udb.3.A, 3udb.4.A, 3udb.5.A, 3udb.6.A, 3ue4.1.A, 3ue4.2.A, 3ugc.1.A, 3uib.1.A, 3ujg.1.A, 3ulz.1.A, 3unj.1.A, 3unk.1.A, 3unz.1.A, 3uo4.1.A, 3uo5.1.A, 3uod.1.A, 3uoj.1.A, 3uok.1.A, 3uol.1.A, 3up2.1.A, 3up7.1.A, 3uqc.1.A, 3uqc.1.B, 3uqc.2.B, 3uqf.2.A, 3uto.1.A, 3uto.2.A, 3uvp.1.A, 3uvq.1.A, 3uvr.1.A, 3uys.2.A, 3uyt.2.A, 3uyt.5.A, 3uyt.5.B, 3uyt.6.A, 3uzp.1.A, 3uzp.2.A, 3uzr.1.A, 3uzs.1.A, 3uzt.1.A, 3v3v.1.A, 3v5l.6.B, 3v5q.1.A, 3v5t.1.A, 3v5w.1.A, 3v6s.1.A, 3v6s.2.A, 3v8s.1.A, 3v8s.1.B, 3vap.1.A, 3vby.1.A, 3vhe.1.A, 3vhk.1.A, 3vid.1.A, 3vjn.1.B, 3vjo.1.A, 3vn9.1.A, 3vnt.1.A, 3vqu.1.A, 3vs1.2.A, 3vs4.1.A, 3vs6.1.A, 3vs7.1.A, 3vs7.2.A, 3vud.1.A, 3vug.1.A, 3vuh.1.A, 3vui.1.A, 3vuk.1.A, 3vul.1.A, 3vum.1.A, 3vut.1.A, 3vut.2.A, 3vvh.1.A, 3vvh.2.A, 3vw6.1.A, 3vw6.1.B, 3vw8.1.A, 3vwa.1.A, 3w0m.1.A, 3w0n.1.A, 3w0p.1.A, 3w0q.1.A, 3w0r.1.A, 3w0s.1.A, 3w10.1.A, 3w16.1.A, 3w18.1.A, 3w18.2.A, 3w1f.1.A, 3w2c.1.A, 3w2o.1.A, 3w2r.1.A, 3w2s.1.A, 3w33.1.A, 3w55.1.A, 3w8l.1.A, 3w8l.2.A, 3w8q.1.A, 3we4.1.A, 3wf7.1.A, 3wf8.1.A, 3wi6.1.J, 3wik.1.A, 3wil.1.A, 3wow.1.A, 3wyx.1.A, 3wzd.1.A, 3wze.1.A, 3wzu.1.A, 3x2u.1.A, 3zbf.1.A, 3zc6.1.A, 3zc6.2.A, 3zdi.1.A, 3zdu.1.A, 3zep.1.A, 3zep.2.A, 3zep.3.A, 3zew.1.A, 3zfm.1.A, 3zfx.1.A, 3zfy.1.A, 3zh8.1.A, 3zhp.1.B, 3zim.1.A, 3zls.1.A, 3zlw.1.A, 3zly.1.A, 3zm9.1.A, 3zm9.2.A, 3zmm.1.A, 3zon.1.A, 3zos.2.A, 3zrk.1.A, 3zsh.1.A, 3zsi.1.A, 3ztx.1.A, 3ztx.2.A, 3zu7.1.A, 3zut.1.A, 3zuu.1.A, 3zuv.1.A, 3zw3.1.A, 3zxt.1.A, 3zxt.1.B, 3zya.1.A, 3zzw.1.A, 3zzw.1.B, 4a07.1.A, 4a4x.1.A, 4a55.1.A, 4a7c.1.A, 4a9t.1.A, 4aaa.1.A, 4acc.1.A, 4acd.1.B, 4ach.1.A, 4ae6.1.A, 4ae6.2.A, 4af3.1.A, 4afj.1.A, 4ag8.1.A, 4agc.1.A, 4agu.1.A, 4agw.1.A, 4aif.1.A, 4aif.2.A, 4ajw.1.A, 4alu.1.A, 4alv.1.A, 4an2.1.A, 4anl.1.A, 4anm.1.A, 4ann.1.A, 4ano.1.A, 4ans.1.A, 4anu.1.A, 4anv.1.A, 4anx.1.A, 4aof.1.A, 4aoj.1.A, 4aot.1.A, 4aot.2.A, 4apc.1.A, 4app.1.A, 4aqc.1.A, 4aqc.2.A, 4ark.1.A, 4as0.1.A, 4asd.1.A, 4asz.1.A, 4at3.1.A, 4at4.1.A, 4at5.1.A, 4au8.1.A, 4aw2.1.A, 4aw5.1.A, 4ax8.1.A, 4azf.1.A, 4azt.1.A, 4azw.1.A, 4b0g.1.A, 4b4l.1.A, 4b6l.1.A, 4b7t.1.A, 4b8l.1.A, 4b8m.1.A, 4b8m.2.A, 4b99.1.A, 4bbe.1.A, 4bbf.3.A, 4bbm.1.A, 4bc6.1.A, 4bcf.1.A, 4bcj.1.A, 4bck.1.A, 4bck.2.A, 4bcm.1.A, 4bco.1.A, 4bcq.1.A, 4bcq.2.A, 4bf2.1.A, 4bf2.2.A, 4bfm.1.A, 4bfr.1.A, 4bfr.2.A, 4bgg.3.A, 4bgq.1.A, 4bib.1.A, 4bib.2.A, 4bkj.2.A, 4bn1.1.A, 4btf.1.A, 4btj.1.A, 4btj.2.A, 4btk.1.A, 4bvu.1.A, 4bwk.1.A, 4bwk.1.B, 4bwp.1.A, 4bwp.1.B, 4bwx.1.A, 4bwx.1.B, 4byi.1.A, 4byj.1.A, 4bzn.1.A, 4c02.1.A, 4c0t.1.A, 4c2v.1.A, 4c2v.2.A, 4c2w.1.A, 4c2w.2.A, 4c33.1.A, 4c34.1.A, 4c35.1.A, 4c3f.1.A, 4c3p.1.A, 4c3p.1.C, 4c3r.1.A, 4c4i.1.A, 4c57.1.A, 4c8b.1.A, 4cfe.1.A, 4cfh.1.A, 4cfu.1.A, 4cfu.2.A, 4cfw.1.A, 4cfw.2.A, 4cfx.1.A, 4cg8.1.B, 4ci6.1.B, 4cki.1.A, 4ckj.1.A, 4ckr.1.A, 4cnh.2.A, 4cqe.1.A, 4cqe.2.A, 4cqg.1.A, 4crs.1.A, 4csv.1.A, 4ct1.1.A, 4cxa.1.A, 4cxa.2.A, 4cyi.1.A, 4cyi.1.B, 4cyj.1.A, 4cyj.1.B, 4czt.1.A, 4czt.1.B, 4czt.2.A, 4czt.2.B, 4czu.1.A, 4czu.1.B, 4czu.2.A, 4czu.2.B, 4czy.1.B, 4czy.1.D, 4d0l.2.A, 4d0l.3.A, 4d0m.1.A, 4d0w.1.A, 4d1s.1.A, 4d28.1.A, 4d28.1.B, 4d28.2.A, 4d28.2.B, 4d2p.1.A, 4d2p.1.B, 4d2p.2.A, 4d2p.2.B, 4d2r.1.A, 4d2s.1.A, 4d2t.2.A, 4d2t.4.A, 4d2w.1.B, 4d4r.1.A, 4d4r.2.A, 4d4s.1.A, 4d4s.2.A, 4d55.1.A, 4d9t.1.A, 4d9u.1.A, 4da5.1.A, 4daw.1.A, 4dbn.1.A, 4dbn.1.B, 4dbx.1.A, 4dc2.1.A, 4dca.1.A, 4dce.1.A, 4de4.2.A, 4ded.1.A, 4dee.1.A, 4deg.1.A, 4dfb.1.A, 4dfb.2.A, 4dfl.1.A, 4dfu.1.A, 4dfu.2.A, 4dfy.1.A, 4dfz.1.A, 4dg3.1.A, 4dgg.1.A, 4dgl.1.C, 4dgl.1.D, 4dgm.1.A, 4dgn.1.A, 4dhf.1.A, 4din.1.A, 4dit.1.A, 4dk5.1.A, 4dn5.3.A, 4dt9.1.A, 4dta.1.A, 4dtb.1.A, 4dtk.1.A, 4dym.1.A, 4e1z.1.A, 4e20.1.A, 4e26.1.B, 4e3c.1.A, 4e3c.1.B, 4e3c.2.A, 4e3c.2.B, 4e3c.3.A, 4e3c.3.B, 4e4m.1.A, 4e4x.1.A, 4e5a.1.A, 4e5b.1.A, 4e6d.1.A, 4e6d.1.B, 4e6q.1.A, 4e73.1.A, 4e7w.1.A, 4e93.1.A, 4ebv.1.A, 4ec9.1.A, 4eev.1.A, 4eh2.1.A, 4eh3.1.A, 4eh4.1.A, 4eh9.1.A, 4ehg.1.B, 4ehv.1.A, 4ejn.1.A, 4el9.1.A, 4enx.1.A, 4eoi.1.A, 4eoi.2.A, 4eoj.1.A, 4eoj.2.A, 4eok.1.A, 4eok.2.A, 4eom.1.A, 4eom.2.A, 4eon.1.A, 4eon.2.A, 4eoo.1.A, 4eoo.2.A, 4eop.1.A, 4eop.2.A, 4eoq.1.A, 4eoq.2.A, 4eor.1.A, 4eor.2.A, 4eos.1.A, 4eos.2.A, 4eqc.1.A, 4eqm.1.A, 4eqm.4.A, 4equ.1.A, 4erk.1.A, 4erw.1.A, 4eut.1.A, 4eut.1.B, 4euu.1.A, 4ewh.1.A, 4ewq.1.A, 4eyj.1.A, 4eym.1.A, 4ez3.1.A, 4ez5.1.A, 4ez7.1.A, 4ezj.1.A, 4ezk.1.A, 4f08.2.A, 4f09.1.A, 4f0f.1.A, 4f0g.1.A, 4f0i.1.A, 4f0i.2.A, 4f1m.1.A, 4f1o.1.A, 4f1s.1.A, 4f1t.1.A, 4f4p.1.A, 4f64.1.A, 4f6s.1.A, 4f7n.1.A, 4f7s.1.A, 4f9a.1.A, 4fa6.1.A, 4fbx.1.A, 4feu.1.A, 4feu.2.A, 4feu.3.A, 4feu.4.A, 4feu.5.A, 4feu.6.A, 4fev.1.A, 4fev.6.A, 4few.1.A, 4fex.3.A, 4fex.5.A, 4ff8.1.A, 4fg7.1.A, 4fg8.1.A, 4fg9.1.A, 4fgb.1.A, 4fie.1.A, 4fif.1.A, 4fih.1.A, 4fjy.1.A, 4fk3.1.A, 4fk3.1.B, 4fk6.1.A, 4fl1.1.A, 4fl2.1.A, 4fl3.1.A, 4fnw.1.A, 4fnx.1.A, 4fny.1.A, 4fnz.1.A, 4fob.1.A, 4fr4.1.A, 4fr4.4.A, 4fsn.1.A, 4fsr.1.A, 4fst.1.A, 4fsu.1.A, 4ful.1.A, 4fv3.1.A, 4fv7.1.A, 4fv8.1.A, 4fvp.1.A, 4fvr.1.A, 4fx3.1.A, 4fza.1.B, 4fzd.1.B, 4fzf.1.B, 4g11.1.A, 4g1w.1.A, 4g2f.1.A, 4g34.1.A, 4g3c.1.A, 4g3c.2.A, 4g3d.1.A, 4g3d.2.A, 4g3f.1.A, 4g3g.1.A, 4g5j.1.A, 4g5p.1.A, 4g5p.2.A, 4g6l.1.A, 4g9c.1.A, 4g9c.2.A, 4g9r.1.A, 4gb9.1.A, 4gcj.1.A, 4geo.1.A, 4gfm.1.A, 4gfo.1.A, 4gg5.1.A, 4gg7.1.A, 4gii.1.A, 4gkh.5.A, 4gl9.1.A, 4gmy.1.A, 4gs6.1.A, 4gsb.1.A, 4gt4.1.A, 4gt5.1.A, 4gu6.2.A, 4gu6.3.A, 4gu9.1.A, 4gub.1.A, 4gv1.1.A, 4gvj.1.A, 4gyg.1.A, 4h05.1.A, 4h05.2.A, 4h1j.1.A, 4h36.1.A, 4h39.1.A, 4h3p.1.A, 4h3q.1.A, 4h58.1.A, 4h58.2.A, 4h58.3.A, 4hct.1.A, 4hge.1.A, 4hge.2.A, 4hgs.1.A, 4hgt.2.A, 4hjo.1.A, 4hle.1.A, 4hnf.1.A, 4hnf.2.A, 4hni.1.A, 4hok.1.A, 4hok.11.A, 4hok.12.A, 4hok.6.A, 4hok.9.A, 4hvb.1.A, 4hvd.1.A, 4hvs.1.A, 4hw7.1.A, 4hyh.1.A, 4hyi.1.A, 4hzr.1.A, 4hzs.1.A, 4i0s.1.A, 4i0t.1.A, 4i1z.1.A, 4i20.1.A, 4i21.1.A, 4i21.2.A, 4i22.1.A, 4i23.1.A, 4i24.1.A, 4i3z.1.A, 4i41.1.A, 4i4f.1.A, 4i5h.1.A, 4i5m.1.A, 4i6h.1.A, 4i92.1.A, 4i93.1.A, 4i93.2.A, 4i94.1.A, 4i94.2.A, 4iaa.1.A, 4ian.1.B, 4iay.1.A, 4ib5.1.A, 4ib5.2.A,

4ib5.3.A, 4ibm.1.A, 4ibm.2.A, 4ic7.1.A, 4ic7.2.A, 4ic8.1.A, 4id7.1.A, 4idt.1.A, 4idt.1.B, 4idv.1.A, 4ieb.1.A, 4ifc.1.A, 4ifc.2.A, 4ifg.1.A, 4ii5.1.A, 4ijp.1.A, 4im0.1.A, 4im3.1.A, 4imy.1.A, 4imy.3.A, 4iq6.1.A, 4ith.1.A, 4ith.2.A, 4iti.1.A, 4iti.2.A, 4itj.1.A, 4itj.2.A, 4iva.1.A, 4iw0.1.A, 4iwd.1.A, 4iwo.1.A, 4iwp.1.A, 4ix3.1.A, 4ix3.1.B, 4ixp.1.A, 4iz5.1.A, 4iz7.1.A, 4iz7.2.A, 4iza.1.A, 4j1r.2.A, 4j1r.4.A, 4j52.1.A, 4j6i.1.A, 4j7b.1.A, 4j8m.1.A, 4j8n.3.A, 4j95.4.A, 4j96.1.A, 4j96.2.A, 4j97.1.A, 4j98.1.A, 4j98.2.A, 4j99.1.A, 4jai.1.A, 4jaj.1.A, 4jbo.1.A, 4jbq.1.A, 4jdj.1.A, 4jg6.1.A, 4jg7.1.A, 4jg8.1.A, 4jia.1.A, 4jin.1.A, 4jjr.1.A, 4jjr.2.A, 4jl9.1.A, 4jlc.1.A, 4jnw.1.A, 4joa.1.A, 4jq7.1.A, 4jr7.1.A, 4jrn.1.A, 4js8.1.A, 4jt3.1.A, 4jvg.1.A, 4jvg.2.A, 4jx3.1.A, 4jxf.1.A, 4k0y.1.A, 4k11.1.A, 4k18.1.A, 4k2r.1.A, 4k33.1.A, 4k6z.1.A, 4k9y.1.A, 4ka3.1.A, 4kao.1.A, 4kao.2.A, 4kb8.1.A, 4kbc.3.A, 4kbc.3.B, 4kd1.1.A, 4kik.1.A, 4kik.1.B, 4kin.4.A, 4kio.2.A, 4kip.1.A, 4kiq.3.A, 4kiq.4.A, 4knb.1.A, 4knb.4.A, 4krc.1.A, 4ks7.1.A, 4ks8.1.A, 4ksp.1.B, 4l00.1.A, 4l00.2.A, 4l01.1.A, 4l01.2.A, 4l1b.1.A, 4l3j.1.A, 4l3p.1.A, 4l42.1.A, 4l43.1.A, 4l44.1.A, 4l45.1.A, 4l46.1.A, 4l52.1.A, 4l67.1.A, 4l68.1.A, 4l68.2.A, 4l6q.1.A, 4l7f.1.A, 4l8m.1.A, 4l9i.1.A, 4lfi.1.B, 4lg4.1.A, 4lg4.2.A, 4lg4.3.A, 4lg4.4.A, 4lg4.5.A, 4lg4.6.A, 4lgd.1.A, 4lgd.2.A, 4lgd.3.A, 4lgd.4.A, 4lgg.1.A, 4lgh.1.A, 4lgh.2.A, 4li5.1.A, 4ll0.1.A, 4ll5.1.A, 4loo.1.A, 4lop.1.A, 4lop.2.A, 4lqm.1.A, 4lqp.1.A, 4lqq.1.A, 4lqq.2.A, 4lqs.1.A, 4lrj.1.A, 4lrk.1.A, 4lrk.2.A, 4lrk.3.A, 4lrm.1.A, 4lrm.2.A, 4lrm.3.A, 4lrm.4.A, 4lrm.5.A, 4lv5.1.A, 4lv8.1.A, 4m0y.1.A, 4m3q.1.A, 4m66.1.A, 4m67.1.A, 4m68.1.A, 4m69.1.A, 4m69.1.B, 4m84.1.A, 4m8t.1.A, 4m97.1.A, 4mao.1.A, 4mbi.1.A, 4mbj.1.A, 4mcv.1.A, 4md7.1.C, 4md8.1.C, 4md9.1.C, 4mf0.1.B, 4mf1.1.B, 4mk0.1.A, 4mkc.1.A, 4mne.1.B, 4mne.1.C, 4mne.1.D, 4mne.2.A, 4mnf.1.A, 4mnf.1.B, 4mq1.1.A, 4mta.1.A, 4mvf.1.A, 4mwi.1.A, 4mx9.1.A, 4mxc.1.A, 4mxo.1.A, 4mxx.1.A, 4mxx.2.A, 4mxy.1.A, 4mxy.2.A, 4myg.1.A, 4myg.2.A, 4n4s.1.A, 4n57.2.A, 4nct.3.A, 4nct.4.A, 4neu.1.A, 4neu.2.A, 4nfm.1.A, 4nfn.1.A, 4nh1.1.B, 4nif.1.A, 4nif.1.D, 4nj3.1.A, 4nm0.1.A, 4nm3.1.A, 4nm5.1.A, 4nst.1.A, 4nt4.1.A, 4nts.1.A, 4nts.2.A, 4ntt.1.A, 4ntt.2.A, 4nus.1.A, 4nw6.1.A, 4nwm.1.A, 4nzw.1.B, 4o0r.1.A, 4o0r.2.A, 4o0s.1.A, 4o0t.1.A, 4o0t.2.A, 4o0u.1.A, 4o0v.1.A, 4o0w.1.A, 4o1o.1.A, 4o21.1.A, 4o27.1.B, 4o2p.1.A, 4o2z.1.A, 4o38.1.A, 4o6e.1.A, 4o6l.2.A, 4o7o.1.A, 4o7o.1.B, 4o7p.1.B, 4o91.2.C, 4o96.1.A, 4o96.2.A, 4oau.1.A, 4oav.1.A, 4oav.1.B, 4obq.1.A, 4obq.1.B, 4ocj.1.A, 4ocu.1.A, 4ogr.2.A, 4oh4.1.A, 4oh4.2.A, 4oli.1.A, 4or5.1.A, 4or5.2.A, 4ork.1.A, 4ork.3.A, 4ork.4.A, 4otd.1.A, 4otf.1.A, 4oth.1.A, 4otp.1.A, 4ouc.1.A, 4ovv.1.A, 4ow8.1.A, 4p2k.1.A, 4p2w.3.B, 4p7e.1.A, 4p7e.2.A, 4p90.1.A, 4p90.2.A, 4pdo.1.B, 4pdp.1.A, 4pdy.1.A, 4ped.1.A, 4pf4.1.A, 4ph4.1.A, 4pl3.1.A, 4pl3.1.B, 4pl4.1.A, 4pl4.1.B, 4pl4.2.A, 4pl4.2.B, 4pl5.1.A, 4pl5.1.B, 4pl5.2.A, 4pl5.2.B, 4pmm.1.A, 4pmt.1.A, 4pni.1.A, 4pnk.1.A, 4pp7.1.A, 4pp9.1.A, 4ppb.3.A, 4ppb.3.B, 4ppc.1.A, 4ppc.2.A, 4pqn.1.A, 4prj.1.A, 4ps8.1.A, 4pte.1.A, 4puz.2.A, 4pv0.1.A, 4pwn.1.A, 4px6.1.A, 4py1.1.A, 4q2a.1.A, 4q5e.1.A, 4q5j.1.A, 4q5j.2.A, 4q9z.1.A, 4q9z.1.B, 4qd6.1.A, 4qd6.2.A, 4qfg.1.A, 4qml.1.A, 4qmn.1.A, 4qmt.1.A, 4qmv.1.A, 4qmw.1.A, 4qny.1.A, 4qo9.1.A, 4qo9.2.A, 4qox.1.A, 4qp1.1.A, 4qp1.2.A, 4qp2.2.A, 4qp3.1.A, 4qp3.2.A, 4qp4.1.A, 4qp4.2.A, 4qpa.1.A, 4qpm.1.A, 4qps.1.A, 4qps.2.A, 4qqc.1.A, 4qqj.1.A, 4qqt.1.A, 4qta.1.A, 4qtb.1.A, 4qtc.1.A, 4qtd.1.A, 4qte.1.A, 4qye.1.A, 4qyy.1.A, 4r1v.1.A, 4r1y.1.A, 4r3c.1.A, 4r3p.1.A, 4r3r.1.A, 4r5s.1.A, 4r5y.1.A, 4r6v.1.A, 4r77.1.A, 4r78.1.A, 4r7b.1.A, 4r8q.1.A, 4ra4.1.A, 4red.1.A, 4red.1.B, 4rer.1.A, 4rew.1.A, 4rfm.1.A, 4rgj.1.A, 4rio.1.A, 4riw.1.A, 4riw.1.B, 4rix.1.A, 4rix.1.B, 4rix.2.B, 4riy.1.A, 4riy.1.B, 4riy.2.B, 4rj4.1.A, 4rll.1.A, 4rlo.1.A, 4rlo.2.A, 4rlp.1.A, 4rmz.1.A, 4rpv.1.A, 4rss.1.A, 4rt7.1.A, 4rvt.1.A, 4rvt.1.B, 4rwi.1.A, 4rwi.2.A, 4rwj.1.A, 4rwj.2.A, 4rwl.2.A, 4rx5.1.A, 4rx9.1.A, 4rz7.1.B, 4rzv.1.A, 4rzv.2.A, 4rzw.1.A, 4rzw.1.B, 4s2z.1.A, 4s30.1.A, 4s32.1.A, 4s33.1.A, 4tks.1.A, 4tl0.1.A, 4tn6.1.A, 4tn6.2.A, 4tnb.1.A, 4tpt.1.A, 4tt7.1.A, 4tth.1.B, 4tuu.1.A, 4tw9.1.A, 4tw9.1.B, 4twn.1.A, 4twp.1.A, 4twp.2.A, 4txc.1.A, 4tye.1.A, 4tyg.1.A, 4tyh.1.A, 4tyh.1.B, 4tyi.1.A, 4tyj.1.A, 4u0i.1.A, 4u3y.1.A, 4u3y.1.B, 4u3z.1.A, 4u3z.1.B, 4u40.2.A, 4u41.2.A, 4u42.1.A, 4u42.1.B, 4u43.1.A, 4u43.3.A, 4u44.2.A, 4u45.2.A, 4u45.3.A, 4u5j.2.A, 4u6r.1.A, 4u79.1.A, 4u81.1.A, 4u8z.1.A, 4u94.1.A, 4u9a.1.A, 4u9a.1.B, 4ual.1.A, 4ub7.1.A, 4uba.1.A, 4uba.2.A, 4ueu.1.A, 4ump.1.A, 4ump.1.B, 4umq.1.A, 4umt.1.A, 4un0.1.B, 4urk.1.A, 4usd.1.A, 4use.2.A, 4usf.1.A, 4usf.2.A, 4uv0.1.A, 4uw0.1.A, 4uwg.1.A, 4uwy.1.A, 4uwy.2.A, 4ux9.1.A, 4ux9.4.A, 4uxq.1.A, 4uy9.1.A, 4uya.1.A, 4uyn.1.A, 4uzd.1.A, 4uzh.1.A, 4v01.2.A, 4v0g.1.A, 4v0g.2.A, 4v0i.2.A, 4w4y.1.A, 4w7p.1.A, 4w8e.1.A, 4w9w.1.A, 4wa9.1.A, 4wa9.2.A, 4wae.1.A, 4waf.1.A, 4waf.1.B, 4wb5.1.A, 4wb6.1.A, 4wb7.1.A, 4wb7.2.A, 4wb8.1.A, 4wbb.1.B, 4wbo.1.A, 4wd5.2.A, 4wd5.3.A, 4wh1.1.A, 4whz.1.A, 4wih.1.A, 4wnk.1.A, 4wnm.1.A, 4wnp.1.A, 4wnp.2.A, 4wnp.4.A, 4wo5.1.A, 4wo5.2.A, 4wot.1.A, 4wov.1.A, 4wsq.1.A, 4wsq.2.A, 4wsy.1.A, 4wua.1.A, 4ww5.1.A, 4x0m.1.A, 4x3f.1.A, 4x3j.1.A, 4x6r.1.A, 4x7q.1.A, 4x7q.2.A, 4xbr.1.A, 4xbu.1.A, 4xcu.1.A, 4xe0.1.A, 4xey.1.A, 4xey.2.A, 4xg3.1.A, 4xh0.1.A, 4xh6.1.A, 4xhk.1.A, 4xhl.1.A, 4xi2.1.A, 4xj0.1.A, 4xj0.2.A, 4xli.1.A, 4xlv.1.A, 4xne.1.A, 4xoy.1.A, 4xr7.1.B, 4xr7.1.C, 4xr7.2.A, 4xr7.2.B, 4xr7.4.A, 4xr7.4.B, 4xrl.1.A, 4xuf.1.A, 4xuf.2.A, 4xv1.1.A, 4xv1.1.B, 4xv9.1.A, 4xx9.1.A, 4y0x.1.A, 4y5h.1.A, 4y73.1.A, 4y73.2.A, 4y73.4.A, 4y83.3.A, 4y85.2.A, 4y8d.2.A, 4y93.1.A, 4y95.1.A, 4y95.2.A, 4ybj.1.B, 4ybk.1.A, 4yc3.1.A, 4yc6.1.A, 4yc8.1.A, 4yff.1.A, 4yfi.1.A, 4yfi.1.B, 4yga.1.A, 4yga.4.A, 4yhf.1.A, 4yhf.2.A, 4yhj.1.A, 4yhj.1.B, 4yht.1.A, 4yjo.1.A, 4yjq.1.A, 4yjt.1.A, 4yju.1.A, 4ykn.1.A, 4ylj.4.A, 4yll.1.A, 4ymj.1.A, 4yne.1.A, 4ynz.1.A, 4ynz.1.B, 4yo6.2.A, 4yo6.4.A, 4yom.1.A, 4yp8.3.A, 4yp8.4.A, 4yr8.1.A, 4yr8.3.A, 4ysj.1.A, 4ysm.1.A, 4ytc.1.A, 4yur.1.A, 4yz9.1.A, 4yz9.2.A, 4yz9.3.A, 4yzb.1.A, 4yzc.1.A, 4yzd.1.A, 4yzd.3.A, 4yzm.1.A, 4yzm.2.A, 4yzn.1.A, 4z16.1.A, 4z3v.1.A, 4z55.1.A, 4z7g.1.A, 4z7h.2.A, 4z83.1.A, 4z84.1.A, 4z9l.1.A, 4zeg.1.A, 4zhx.1.A, 4zhx.2.A, 4zji.1.A, 4zji.3.A, 4zjv.1.A, 4zlo.1.A, 4zlo.1.B, 4zly.1.A, 4zog.1.A, 4zog.2.A, 4zop.1.A, 4zop.1.B, 4zp5.1.A, 4zp5.2.A, 4zs0.1.A, 4zsa.1.A, 4zse.1.A, 4zse.1.B, 4zse.2.A, 4zsg.1.A, 4zsj.1.A, 4zsl.1.A, 4ztl.1.A, 4ztr.1.A, 4zts.1.A, 4zy4.1.A, 4zy4.2.A, 4zy6.1.A, 4zy6.2.A, 4zzm.1.A, 5a14.1.A, 5a46.1.A, 5a4c.1.A, 5a4e.3.A, 5a6n.1.A, 5a6n.1.B, 5a9u.1.A, 5aa8.1.A, 5aa9.1.A, 5aad.1.A, 5aae.1.A, 5acb.1.B, 5acb.2.B, 5afv.1.B, 5aik.2.A, 5air.1.A, 5air.1.B, 5ajq.1.A, 5ajq.2.A, 5am6.2.A, 5am7.1.A, 5am7.2.A, 5amn.1.A, 5ap1.1.A, 5ap2.1.A, 5ap3.1.A, 5ap6.1.A, 5ap7.1.A, 5ar2.1.A, 5ar4.1.B, 5ar7.1.A, 5ar7.1.B, 5ar8.1.A, 5av4.1.A, 5awm.1.A, 5ax3.1.A, 5ax9.1.A, 5ax9.2.A, 5ax9.3.A, 5b0x.1.A, 5b2k.1.A, 5b2l.1.A, 5b2m.1.A, 5b7v.2.A, 5bmm.2.A, 5bms.1.A, 5bpy.1.A, 5bpy.2.A, 5bue.1.A, 5bvf.1.A, 5bvk.1.A, 5bvo.1.A, 5byy.1.A, 5byz.1.A, 5c01.1.A, 5c03.2.A, 5c46.1.A, 5c4k.2.A, 5c4l.1.A, 5c4l.2.A, 5cav.1.A, 5cei.1.A, 5cek.1.A, 5cem.1.A, 5cen.1.A, 5ceo.1.A, 5cf5.1.A, 5cf5.2.A, 5ci6.1.A, 5ci6.2.A, 5ckw.1.A, 5ckw.1.B, 5clr.1.A, 5clr.1.B, 5cnn.1.A, 5cno.2.A, 5cs6.1.A, 5csp.1.A, 5csw.1.A, 5csx.1.A, 5ct7.1.A, 5cu3.1.A, 5cu4.1.A, 5cu6.1.A, 5cvg.1.A, 5cwz.1.A, 5cwz.2.A, 5cwz.3.A, 5cxh.1.A, 5cxz.1.A, 5cy3.1.A, 5cyi.1.A, 5cyz.1.A, 5czh.1.A, 5czo.1.A, 5d10.1.A, 5d10.2.A, 5d11.1.A, 5d12.1.A, 5d12.2.A, 5d41.1.B, 5d7a.1.A, 5d7a.3.A, 5d7v.1.A, 5d9h.1.A, 5d9k.1.A, 5d9l.1.A, 5da3.1.A, 5dbx.1.A, 5dbx.1.B, 5de2.1.A, 5de2.2.A, 5dew.2.A, 5dey.1.A, 5dey.2.A, 5dfp.1.A, 5dfz.1.B, 5dfz.1.C, 5dg5.1.B, 5dgz.1.A, 5dh3.1.A, 5di1.1.A, 5di1.2.A, 5dmz.1.A, 5dmz.2.A, 5dnr.1.A, 5dos.1.A, 5drb.1.A, 5dt0.1.A, 5dt4.1.A, 5dvr.1.A, 5dxh.1.A, 5dxt.1.A, 5dxu.1.A,

5dyk.1.A, 5e1e.2.A, 5e1s.1.A, 5e7r.1.A, 5e8s.1.A, 5e8u.1.A, 5e8v.1.A, 5e8x.1.A, 5eak.1.A, 5ebz.1.A, 5ebz.1.F, 5ebz.2.E, 5ebz.2.F, 5edp.1.A, 5edr.1.A, 5eds.1.A, 5efq.1.A, 5eg3.1.A, 5ek7.1.A, 5eob.1.A, 5eqp.1.A, 5eqy.1.A, 5es1.1.A, 5eta.1.A, 5eta.2.A, 5etc.1.A, 5etf.1.A, 5eti.1.A, 5ew3.1.A, 5ew3.2.A, 5ew9.1.A, 5eyk.1.A, 5eym.1.A, 5ezr.1.A, 5ezv.1.A, 5ezv.2.A, 5f1z.1.A, 5f4n.1.A, 5f94.1.A, 5f9e.1.A, 5f9e.2.A, 5fbo.1.A, 5fbw.1.A, 5fd2.1.A, 5fd2.1.B, 5fdp.1.A, 5fdx.1.A, 5fed.1.A, 5fee.1.A, 5fg8.1.A, 5fgk.1.A, 5fi4.1.B, 5flf.1.A, 5flf.2.A, 5flf.3.A, 5flf.5.A, 5fm2.1.A, 5fqd.1.C, 5fri.1.A, 5ftg.1.B, 5fwl.1.D, 5fxq.1.A, 5fxr.1.A, 5fxs.1.A, 5g15.1.A, 5g1x.1.A, 5g2n.1.A, 5g55.1.A, 5g6v.1.A, 5g6v.2.A, 5ghv.1.A, 5gjd.1.A, 5gjg.1.A, 5gjk.1.A, 5gmp.1.A, 5gnk.1.A, 5grn.1.A, 5gty.2.A, 5gty.2.C, 5gty.2.D, 5gtz.1.A, 5gz8.1.A, 5gza.1.A, 5h09.1.A, 5h0e.1.A, 5h2u.1.A, 5h3q.1.A, 5h8g.1.A, 5hbe.1.A, 5hbh.1.A, 5hcz.1.A, 5hd7.1.A, 5he0.1.A, 5he1.1.A, 5he3.1.A, 5hes.1.A, 5hez.1.A, 5hez.2.A, 5hg9.1.A, 5hgi.1.A, 5hhw.1.A, 5hi2.1.A, 5hie.1.A, 5hie.3.A, 5hie.4.A, 5hln.1.A, 5hln.1.B, 5hlp.2.A, 5hlw.1.A, 5hnb.1.A, 5hni.1.A, 5hnv.1.A, 5hoa.1.A, 5hor.1.A, 5hti.1.A, 5hu3.1.A, 5hu9.1.A, 5hvj.1.A, 5hvj.2.A, 5hvk.1.A, 5hvk.2.A, 5hvu.1.B, 5hvy.1.A, 5hx6.1.A, 5hx6.2.A, 5hze.1.A, 5hzn.7.A, 5hzn.8.A, 5i0b.1.A, 5i35.1.A, 5i3o.1.A, 5i3o.2.A, 5i4n.1.A, 5i4u.1.A, 5i5z.1.A, 5i6u.1.A, 5i8a.1.A, 5i9v.1.A, 5i9w.1.A, 5i9y.1.A, 5ia0.2.A, 5ia0.3.A, 5ia1.1.A, 5ia4.1.A, 5idn.1.A, 5idp.1.A, 5if1.1.A, 5if1.2.A, 5ig1.1.A, 5ig1.2.A, 5igh.1.A, 5igi.1.A, 5igv.1.A, 5igy.1.A, 5igz.1.A, 5ih0.1.A, 5ih1.1.A, 5ih4.1.A, 5iha.1.A, 5ikw.1.A, 5ime.1.A, 5ime.2.A, 5imx.1.A, 5iqc.1.A, 5iqe.3.A, 5iqg.2.A, 5iqh.1.A, 5iqh.4.A, 5iqi.1.A, 5iqi.4.A, 5is5.1.A, 5iso.1.A, 5iso.2.A, 5ita.1.A, 5ita.2.A, 5iu2.1.A, 5iuh.1.A, 5iwu.1.A, 5izj.1.A, 5izj.2.A, 5j0a.1.A, 5j0a.1.B, 5j1v.2.A, 5j5s.1.A, 5j5t.1.A, 5j79.1.A, 5j7s.1.A, 5j87.1.A, 5j87.1.D, 5j8i.1.A, 5j95.1.A, 5j95.2.A, 5j9l.1.A, 5j9y.1.A, 5j9z.1.A, 5jeb.1.A, 5jfs.1.A, 5jgb.1.A, 5jh6.1.A, 5jha.1.A, 5jhb.1.A, 5jk3.1.A, 5jkg.1.A, 5jms.1.A, 5jr7.1.A, 5jr7.2.A, 5jrq.1.A, 5jsm.2.A, 5jsm.2.B, 5jy7.1.E, 5jy7.1.F, 5jy7.1.H, 5jzj.1.A, 5jzj.2.A, 5jzn.1.A, 5k00.1.A, 5k0k.1.A, 5k0k.2.A, 5k3y.1.A, 5k3y.2.A, 5k4i.1.A, 5k4j.1.A, 5k5n.1.A, 5k5x.1.A, 5k75.4.A, 5k7g.3.A, 5k7i.1.A, 5k9i.2.A, 5kbq.1.A, 5kbq.2.A, 5kbr.1.A, 5kc2.1.A, 5kc2.1.B, 5kcv.1.A, 5kcx.1.A, 5khw.2.A, 5khx.1.A, 5kkr.1.A, 5kkr.1.B, 5kmi.1.A, 5kmk.1.A, 5kml.1.A, 5knj.1.A, 5ko1.1.A, 5kpk.2.A, 5ku8.1.A, 5kvt.1.A, 5kx7.2.A, 5kx8.2.A, 5kz0.1.A, 5kz7.1.A, 5kz7.2.A, 5l1z.1.A, 5l2i.1.A, 5l2q.1.A, 5l2q.3.A, 5l2q.4.A, 5l2s.1.A, 5l2t.1.A, 5l2w.1.A, 5l3a.1.A, 5l4q.1.A, 5l8k.1.A, 5l8l.1.A, 5lar.1.A, 5lcj.1.A, 5lck.1.A, 5lcq.1.A, 5lcr.1.A, 5li1.1.A, 5li9.1.A, 5lih.1.A, 5ljj.1.A, 5lma.1.A, 5lmk.2.A, 5loh.1.A, 5lpw.1.A, 5lpz.1.A, 5lqf.1.A, 5lvp.1.A, 5lw1.2.B, 5lwm.1.A, 5lxc.1.A, 5lxd.1.A, 5lxm.1.A, 5m06.1.A, 5m07.1.A, 5m08.1.A, 5m08.2.A, 5m09.1.A, 5m09.2.A, 5m0l.1.A, 5m44.1.A, 5m4c.1.A, 5m4f.1.A, 5m4i.1.A, 5m4u.1.A, 5m51.1.A, 5m53.1.A, 5m56.1.A, 5m56.2.A, 5m57.1.A, 5m6u.1.A, 5mag.1.A, 5mah.1.A, 5mai.1.A, 5mja.1.A, 5mjb.1.A, 5ml5.1.A, 5mo4.1.A, 5mov.1.A, 5mow.2.A, 5mqv.1.A, 5mqv.5.A, 5mrb.1.A, 5mrd.1.A, 5mty.1.A, 5mxx.1.A, 5my8.1.A, 5myv.2.A, 5myv.3.A, 5myv.4.A, 5mz3.1.A, 5n1g.1.A, 5n1v.1.A, 5n23.1.A, 5n4v.1.A, 5n63.1.A, 5n65.1.A, 5n7v.1.A, 5n84.1.A, 5n93.1.A, 5na0.1.A, 5nad.1.A, 5ncl.1.A, 5ncy.1.A, 5ncz.1.A, 5nev.1.A, 5ng0.1.A, 5ng2.1.A, 5ng3.1.A, 5ngb.1.A, 5ngu.1.A, 5nhh.1.A, 5nk0.1.A, 5nk3.1.A, 5nk5.1.A, 5nk7.1.A, 5nka.1.A, 5nqc.1.A, 5ntj.1.A, 5ntt.1.A, 5nud.1.A, 5nud.2.A, 5nwz.1.A, 5nwz.2.A, 5nxc.1.A, 5nxd.1.A, 5nxd.2.A, 5nzz.1.B, 5nzz.2.B, 5o0y.1.A, 5o11.1.A, 5o1s.1.A, 5o1v.1.A, 5o1v.1.B, 5o21.1.A, 5o23.1.A, 5o23.2.A, 5o26.1.A, 5o2b.1.A, 5o2b.2.A, 5o2c.1.A, 5o7i.1.A, 5o83.1.A, 5o8u.1.A, 5o8v.1.A, 5o90.1.A, 5oat.1.A, 5obj.1.A, 5obr.1.A, 5odt.1.A, 5okt.2.A, 5okt.4.A, 5omg.1.A, 5omy.1.A, 5one.1.A, 5oni.1.A, 5oo0.1.A, 5oo1.1.A, 5ooi.1.A, 5oop.1.A, 5op2.1.A, 5opu.1.A, 5opv.1.A, 5oq4.1.A, 5oq5.1.A, 5oq6.1.A, 5oq7.1.A, 5oq8.1.A, 5orl.1.A, 5os5.1.A, 5os7.1.A, 5osd.1.A, 5osl.1.A, 5osm.1.A, 5osz.1.A, 5otq.1.A, 5owl.1.A, 5owq.3.B, 5owr.1.A, 5oy4.1.A, 5oy6.4.A, 5p9f.1.A, 5p9k.1.A, 5p9m.1.A, 5r8z.1.A, 5r9d.1.A, 5s7g.1.A, 5s82.1.B, 5sav.1.A, 5saw.1.A, 5sax.1.A, 5say.1.A, 5say.2.A, 5sb1.1.A, 5sb2.1.A, 5sw8.1.A, 5swh.1.A, 5sx8.1.A, 5sxa.1.A, 5sys.1.A, 5t0p.1.A, 5t1t.1.A, 5t23.1.A, 5t27.1.A, 5t31.1.A, 5t5t.2.A, 5t68.1.A, 5t7f.1.A, 5t8f.1.A, 5t8i.1.A, 5t8o.1.A, 5t8p.1.A, 5t8q.1.A, 5t8q.2.A, 5ta6.1.A, 5tbe.1.A, 5tc0.1.A, 5tc0.2.A, 5tco.1.A, 5td2.1.A, 5td2.2.A, 5te0.1.A, 5teh.1.A, 5teh.2.A, 5tex.1.A, 5tf9.1.A, 5tf9.2.A, 5tiu.1.A, 5tkd.1.A, 5to8.1.A, 5tob.1.A, 5toe.1.A, 5tos.1.A, 5tq4.1.A, 5tq6.1.A, 5tq7.1.A, 5tq7.2.A, 5tq8.1.A, 5tqw.1.A, 5tqx.1.A, 5tqy.1.A, 5tr6.1.A, 5ts8.1.A, 5tts.1.A, 5tur.1.A, 5tvt.1.A, 5twl.1.A, 5twy.1.A, 5twy.2.A, 5twz.1.A, 5tx3.2.A, 5u6b.1.A, 5u6b.2.A, 5u6c.1.A, 5u6c.1.B, 5u6i.1.A, 5u6y.1.A, 5u6y.1.B, 5u6y.1.C, 5u6y.1.D, 5u6y.1.E, 5u6y.1.F, 5u6y.1.G, 5u6y.1.H, 5u6y.1.I, 5u6y.1.J, 5u6y.1.K, 5u6y.1.L, 5u7q.1.A, 5u7q.2.A, 5u7r.1.A, 5u7r.1.B, 5u7r.2.B, 5u94.1.A, 5u9d.1.A, 5uab.1.A, 5ubr.1.A, 5ubt.1.A, 5ugc.1.A, 5ugl.1.A, 5ugx.1.A, 5uhn.1.A, 5ui0.1.A, 5ui0.2.A, 5uiq.1.A, 5uir.1.A, 5uit.1.A, 5uiu.2.A, 5uk8.1.A, 5uk8.1.B, 5ukf.1.A, 5ukf.2.A, 5ukk.1.A, 5ukl.1.A, 5ukm.1.A, 5ul1.1.B, 5umo.1.A, 5unp.1.A, 5uor.1.A, 5uor.2.A, 5uox.2.A, 5up3.1.A, 5upk.1.B, 5upl.1.A, 5uq0.1.A, 5uq1.1.A, 5uq2.1.A, 5uq3.1.A, 5ur1.1.A, 5usq.1.A, 5usy.1.A, 5uu1.1.A, 5uuu.1.A, 5uv4.1.A, 5uvc.1.A, 5uwd.1.A, 5uxa.2.A, 5uxb.1.A, 5uxb.2.A, 5uxc.1.A, 5uxd.1.A, 5uxd.2.A, 5uy6.1.A, 5v5n.1.A, 5v5y.1.A, 5v60.1.A, 5v61.1.A, 5v62.1.A, 5val.2.A, 5vam.1.A, 5vc3.1.A, 5vc4.1.A, 5vc5.1.A, 5vcv.1.A, 5vcw.1.A, 5vcw.2.A, 5vcx.1.A, 5vcy.1.A, 5vd0.1.A, 5vd1.1.A, 5vd2.1.A, 5vd4.1.A, 5vdk.1.A, 5ve6.1.A, 5ved.1.A, 5vee.1.A, 5vef.1.A, 5vib.1.A, 5vil.1.A, 5vil.2.A, 5vil.3.A, 5vil.4.A, 5vja.1.B, 5vja.1.D, 5vlo.1.A, 5vlo.2.A, 5vnd.1.A, 5vnd.2.A, 5vt1.1.A, 5w4w.2.A, 5w5j.1.A, 5w5j.1.B, 5w5o.1.A, 5w5v.1.A, 5w7t.1.A, 5w7t.2.A, 5w84.1.A, 5w86.1.A, 5w86.3.A, 5wax.1.A, 5wax.2.A, 5wdy.1.A, 5wdy.2.A, 5we8.1.A, 5wev.1.A, 5wg4.1.A, 5wg5.1.A, 5wij.1.A, 5wjj.1.A, 5wne.2.A, 5wng.1.A, 5wng.1.B, 5wnh.2.A, 5wnh.2.B, 5wni.1.A, 5wno.1.A, 5wp1.1.A, 5wr7.1.A, 5wvd.1.A, 5x02.1.A, 5x17.1.A, 5x17.2.A, 5x18.1.A, 5x2f.1.A, 5x2f.2.A, 5x2f.2.B, 5x2k.1.A, 5x3f.1.B, 5x5o.1.A, 5x8i.1.A, 5xd6.1.A, 5xd6.2.A, 5xdl.1.A, 5xff.1.A, 5xfj.1.A, 5xgi.1.A, 5xgj.1.A, 5xgm.1.A, 5xgn.1.A, 5xgn.1.B, 5xka.1.A, 5xp7.2.A, 5xqx.1.A, 5xv7.1.A, 5xva.1.A, 5xvf.1.A, 5xvu.1.A, 5xy1.1.A, 5xyx.1.A, 5xyz.1.A, 5xzv.1.A, 5xzv.2.A, 5y25.1.A, 5y5t.1.A, 5y5u.1.A, 5y5u.2.A, 5y7z.1.A, 5y80.1.A, 5y86.1.A, 5y8u.1.A, 5y90.1.A, 5y9m.1.A, 5y9t.1.A, 5ya5.1.A, 5yj9.1.A, 5yjz.1.A, 5yks.1.A, 5yt3.1.A, 5yt3.1.B, 5yt3.2.A, 5yu9.3.A, 5yu9.4.A, 5yvb.1.A, 5z1d.1.A, 5z1e.1.A, 5z33.1.A, 5zan.1.A, 5zj6.1.A, 5zn0.1.A, 5zn2.1.A, 5zn3.1.A, 5zn4.1.A, 5zn5.1.A, 5zto.1.A, 5zv2.1.A, 5zv2.2.A, 5zwj.1.A, 5zxb.1.A, 5zxb.1.B, 5zz4.1.A, 6a1c.1.A, 6a1f.1.A, 6a1g.1.A, 6a32.1.A, 6aaj.1.A, 6aaj.1.B, 6aak.1.B, 6aak.2.A, 6aam.1.A, 6ac9.1.A, 6ac9.3.A, 6ae3.1.A, 6ae3.2.A, 6agx.1.A, 6agx.1.B, 6ao5.1.A, 6ate.1.A, 6ath.1.A, 6aua.1.A, 6aub.1.A, 6aud.1.A, 6ayd.1.A, 6b1u.2.A, 6b2e.1.A, 6b2p.1.A, 6b2q.1.A, 6b3e.1.A, 6b4w.1.A, 6b5j.4.A, 6b8j.1.A, 6b8y.1.A, 6bab.1.A, 6bab.2.A, 6bbu.1.A, 6bbv.1.A, 6bdl.1.A, 6bdn.1.A, 6bfn.1.A, 6bfn.2.A, 6bg2.1.A, 6bg2.2.A, 6bg2.3.A, 6bhc.1.A, 6bik.1.A, 6bl8.1.A, 6bl8.2.A, 6ble.1.A, 6bny.1.A, 6bod.1.A, 6bq1.1.A, 6bq1.1.D, 6bql.1.A, 6bqq.1.A, 6brj.1.A, 6bsd.1.A, 6buu.1.A, 6bwk.1.A, 6bx6.1.A, 6bxi.1.A, 6byr.1.C, 6byr.2.A, 6bys.2.C, 6c0u.1.A, 6c1s.1.A, 6c2t.1.B, 6c2y.1.A, 6c3e.1.A, 6c3e.1.B, 6c4d.1.A, 6c4d.2.A, 6c4d.3.A, 6c4d.4.A, 6c7y.1.A, 6c83.1.A, 6c83.1.B, 6c9d.1.A, 6c9j.1.A, 6cad.1.A, 6cad.2.A, 6ccf.1.A, 6ccy.1.A, 6cd6.1.A, 6cd7.1.A, 6cd7.2.A, 6ch4.1.A, 6cjw.1.A, 6cmj.1.A, 6cn9.1.A, 6cn9.2.A, 6cnh.1.A,

6cpe.1.A, 6cpf.1.A, 6cpw.1.A, 6cpy.1.A, 6cpy.2.A, 6cq0.1.A, 6cqd.1.A, 6cqd.1.B, 6cqe.1.A, 6cqf.1.A, 6csw.4.A, 6cth.1.A, 6ctz.1.A, 6cz3.1.A, 6cz4.1.A, 6d1y.1.A, 6d22.1.A, 6d2i.1.A, 6d5y.1.A, 6d8e.1.A, 6dc0.1.A, 6dc0.1.B, 6dfl.1.A, 6dgt.1.A, 6di5.1.A, 6di9.1.A, 6dkb.1.A, 6dkg.1.A, 6dkw.1.A, 6dkw.2.A, 6dmg.1.A, 6dtl.1.A, 6dtl.2.A, 6duk.1.A, 6duk.3.A, 6e0r.1.A, 6e2o.1.A, 6e2o.1.B, 6e4f.1.A, 6e6e.1.A, 6e6e.4.A, 6e6e.5.A, 6e6e.8.A, 6e9w.1.A, 6e9w.1.B, 6eac.1.A, 6eas.1.A, 6ed6.1.A, 6ed6.2.A, 6edl.1.A, 6ef6.1.A, 6eg9.1.A, 6eg9.1.B, 6ega.1.B, 6egd.1.A, 6egf.1.A, 6ehu.1.A, 6ehu.2.A, 6eif.2.A, 6eij.4.A, 6eil.2.A, 6eim.1.A, 6eim.1.B, 6eiq.1.A, 6eiv.2.A, 6eiv.3.A, 6eix.1.A, 6ej4.1.A, 6ej4.2.A, 6ej4.3.A, 6ej4.4.A, 6ekd.1.A, 6elr.1.A, 6emd.1.A, 6emh.4.A, 6eml.1.4, 6ep9.1.A, 6eq9.1.A, 6eq9.2.A, 6eqi.1.C, 6es0.1.A, 6es0.2.A, 6esa.1.A, 6ewx.1.A, 6ewx.1.B, 6eyz.1.A, 6f14.1.A, 6f1w.1.A, 6f1w.2.A, 6f26.1.A, 6f26.2.A, 6f3d.1.A, 6f3e.2.A, 6f3f.1.A, 6f3i.1.A, 6f5e.1.B, 6f7b.1.A, 6fad.1.A, 6fad.3.A, 6fai.1.J, 6fcf.1.A, 6fd3.1.A, 6fdm.1.A, 6fdm.1.B, 6fdn.1.A, 6fdn.1.B, 6fdo.1.A, 6fdo.2.A, 6fdy.1.A, 6fdz.1.A, 6fek.1.A, 6fer.1.A, 6few.1.A, 6fha.1.A, 6fhb.1.A, 6fil.1.A, 6fio.1.A, 6fiq.1.A, 6fjz.1.A, 6fnf.1.A, 6fng.1.A, 6fnh.1.A, 6fnh.2.A, 6fnh.3.A, 6fni.1.A, 6fnj.1.A, 6fnl.1.A, 6frx.1.A, 6ft7.1.A, 6ft9.3.A, 6fu5.1.B, 6fuc.1.A, 6fvf.1.A, 6fyi.1.A, 6fyk.1.A, 6fyk.2.A, 6fyk.3.A, 6fyl.1.A, 6fyo.1.A, 6fyp.1.A, 6fyr.1.A, 6fyv.1.A, 6g18.1.7, 6g39.1.A, 6g3a.1.A, 6g3c.1.A, 6g4j.1.A, 6g51.1.W, 6g54.1.A, 6g6w.1.A, 6g76.1.A, 6g78.1.A, 6g9a.1.A, 6g9n.1.A, 6gcw.1.A, 6gcw.1.B, 6gcx.1.A, 6ggh.1.A, 6gjo.2.A, 6gl3.1.A, 6gl3.2.A, 6gn1.1.A, 6gqj.1.A, 6gqk.1.A, 6gqo.1.A, 6gr8.1.A, 6gr9.1.A, 6gra.1.A, 6gtt.1.A, 6gu2.1.A, 6gu6.1.A, 6gu7.2.A, 6gub.1.A, 6gub.2.A, 6guc.2.A, 6gue.1.A, 6gue.2.A, 6guf.1.A, 6guf.2.A, 6guh.1.A, 6guk.1.A, 6gva.1.A, 6gvf.1.A, 6gvg.1.A, 6gvj.1.A, 6gvx.1.A, 6gwr.1.A, 6gwr.1.B, 6gy0.1.A, 6gzd.1.A, 6gzh.1.A, 6gzm.1.A, 6gzm.1.B, 6h0u.1.B, 6h3k.1.A, 6hbn.2.A, 6hdr.1.A, 6hh1.1.A, 6hhf.1.A, 6hhg.1.A, 6hhh.1.A, 6hho.1.A, 6hho.2.A, 6hi9.1.A, 6hjj.1.A, 6hjk.1.A, 6hk3.1.A, 6hk3.2.A, 6hk4.2.A, 6hk6.1.A, 6hk6.2.A, 6hk7.1.A, 6hkm.1.A, 6hkn.1.A, 6hm6.1.A, 6hmd.1.A, 6hme.1.A, 6hmp.1.A, 6hmq.1.A, 6hmr.2.A, 6hmx.1.A, 6hp9.1.A, 6hp9.2.A, 6hv0.1.A, 6hvd.1.A, 6hvf.2.A, 6hwj.1.A, 6hwj.2.A, 6hwk.1.A, 6hwk.1.D, 6hwu.1.A, 6hwv.1.A, 6hx1.1.A, 6hxf.1.A, 6hxf.3.A, 6hzu.2.A, 6hzv.1.A, 6i2p.1.A, 6i2u.1.A, 6i2y.1.A, 6i2y.1.B, 6i3u.1.A, 6i5i.1.A, 6i82.1.A, 6i82.1.B, 6i83.1.A, 6i8z.1.A, 6i99.1.A, 6i99.2.A, 6ib0.1.A, 6ib2.1.A, 6ig8.1.A, 6iii.1.A, 6il3.1.A, 6ilz.1.A, 6ilz.2.A, 6ilz.3.A, 6ilz.4.A, 6in0.1.A, 6in4.1.A, 6iqn.1.A, 6iqn.1.B, 6ise.1.A, 6itt.1.A, 6itt.1.B, 6itv.1.A, 6iup.1.A, 6iy9.1.A, 6iy9.2.A, 6j5l.1.A, 6j5t.1.A, 6j5t.1.B, 6j5u.1.B, 6j5u.1.C, 6j5v.1.B, 6j5v.1.C, 6j5w.1.A, 6j6i.1.A, 6j6i.1.B, 6jgm.1.A, 6jkk.1.A, 6jlr.1.A, 6jmf.1.A, 6joi.1.A, 6joj.1.A, 6jol.1.A, 6jpe.1.A, 6jpj.1.A, 6jqr.1.A, 6jrj.1.A, 6jrk.1.A, 6jrx.1.A, 6jut.1.A, 6jux.1.A, 6jwl.1.A, 6jx0.1.A, 6jxt.1.A, 6jz0.1.A, 6k0j.1.A, 6k20.1.A, 6k3l.1.A, 6ka4.1.A, 6khd.1.A, 6khd.2.A, 6khd.3.A, 6khe.1.A, 6khf.1.A, 6kla.1.A, 6kmh.1.A, 6kmh.2.A, 6kyq.1.A, 6kyr.1.A, 6kz7.1.A, 6kzc.1.A, 6l20.1.A, 6l20.2.A, 6l20.3.A, 6l20.4.A, 6l21.1.A, 6l22.1.A, 6l23.1.A, 6l24.1.A, 6l8l.1.A, 6l8l.2.A, 6l8l.3.A, 6l8l.4.A, 6lba.1.A, 6lk5.1.A, 6lk6.1.A, 6ln1.1.A, 6ln1.2.A, 6lnm.1.A, 6lub.1.A, 6lvk.1.A, 6lvm.1.A, 6m0u.1.A, 6m11.1.B, 6m12.1.A, 6m7z.1.A, 6m7z.3.A, 6m7z.4.A, 6m7z.5.A, 6m7z.6.A, 6m95.1.A, 6m9l.1.A, 6mcp.1.A, 6mcp.2.A, 6mib.1.A, 6mm5.1.A, 6mm6.1.A, 6mnh.1.A, 6mny.1.A, 6mny.2.A, 6mob.1.A, 6mom.1.A, 6mul.1.A, 6mwe.2.A, 6mx8.1.A, 6myn.1.A, 6mzw.1.A, 6n0p.1.A, 6n6o.1.A, 6n7a.1.A, 6n7c.2.A, 6n8g.1.A, 6n8g.3.A, 6nbs.1.A, 6ncg.1.A, 6ncg.2.A, 6nct.1.A, 6ne7.1.A, 6nfi.1.A, 6nfy.1.A, 6nfz.1.A, 6nfz.1.B, 6ng0.1.A, 6ng0.1.B, 6no7.1.C, 6no7.2.A, 6no7.2.C, 6no8.1.A, 6npt.1.A, 6npy.1.B, 6npz.1.A, 6npz.2.A, 6nsp.1.A, 6nsq.1.A, 6nss.1.A, 6nt9.1.A, 6nvj.1.A, 6nvl.1.A, 6nvl.2.A, 6nvl.3.A, 6nw2.1.A, 6nw2.2.A, 6nyb.1.A, 6nyb.1.B, 6nyh.1.A, 6nyh.2.A, 6nzm.1.A, 6nzr.1.A, 6o5z.1.A, 6o5z.2.A, 6o6q.1.A, 6o6q.1.B, 6o8b.1.C, 6o8b.1.D, 6o8c.1.A, 6o8c.1.B, 6o8i.1.A, 6o8u.1.A, 6o8u.2.A, 6o8u.3.A, 6o94.1.A, 6o94.2.A, 6o94.3.A, 6o9d.2.A, 6o9l.1.3, 6oac.1.A, 6ocq.1.A, 6ocq.2.A, 6ocu.1.A, 6ohd.1.A, 6oid.1.A, 6oko.1.A, 6ol2.1.A, 6op9.1.A, 6opg.1.A, 6opi.1.A, 6opk.1.A, 6oqi.1.A, 6oql.1.A, 6ot6.1.A, 6ots.1.A, 6ova.1.A, 6oyt.1.A, 6oyt.1.B, 6oyw.1.A, 6oyw.1.B, 6p1d.1.A, 6p1d.1.B, 6p1d.2.A, 6p1d.2.B, 6p1l.1.B, 6p1l.2.A, 6p3d.1.A, 6p5m.1.A, 6p5m.3.A, 6p5p.4.A, 6p5s.1.A, 6p68.2.A, 6p69.1.A, 6p7g.1.A, 6p7g.1.B, 6p7g.2.B, 6p8e.1.B, 6p8f.1.B, 6p8g.1.B, 6p8h.1.B, 6p8q.1.A, 6p8q.2.A, 6paw.1.A, 6paw.2.A, 6paw.4.A, 6pdj.1.A, 6pjj.1.A, 6pjx.1.A, 6pk6.1.A, 6pl2.1.A, 6pl3.1.A, 6pma.1.A, 6pme.1.B, 6pme.1.D, 6pme.1.L, 6pnx.1.A, 6pnx.1.B, 6pp9.1.A, 6pp9.1.B, 6pwd.1.A, 6pwg.1.A, 6pxn.1.A, 6pxn.2.A, 6pxo.3.A, 6pxo.3.B, 6pxp.1.A, 6pxp.2.A, 6pyr.1.A, 6pys.1.A, 6q0j.1.A, 6q0k.1.A, 6q0k.1.B, 6q0t.1.A, 6q0t.1.B, 6q0t.1.C, 6q2a.1.A, 6q2a.15.A, 6q2a.2.A, 6q2a.7.A, 6q38.1.A, 6q48.1.A, 6q4d.1.A, 6q4g.1.A, 6q4h.1.A, 6q4i.1.A, 6q4k.1.A, 6q4q.1.A, 6q8k.1.A, 6qaq.1.A, 6qas.1.A, 6qas.2.A, 6qat.1.A, 6qat.2.A, 6qau.1.A, 6qau.2.A, 6qav.1.A, 6qav.2.A, 6qav.2.B, 6qfl.1.A, 6qfr.1.A, 6qmo.1.A, 6qn4.1.A, 6qp5.1.A, 6qtg.1.A, 6qx9.56.A, 6qy7.1.A, 6qy9.1.A, 6qyx.1.A, 6r3d.1.A, 6r5f.2.A, 6r5f.3.A, 6r5f.4.A, 6r5k.1.F, 6r5k.1.G, 6ra5.1.A, 6ra7.1.A, 6rbd.1.W, 6rcg.1.A, 6rch.1.A, 6rch.1.B, 6rct.2.A, 6rfi.2.A, 6rfo.1.A, 6rfp.1.A, 6rln.1.A, 6rsb.1.A, 6rsr.1.A, 6rst.1.A, 6rsu.1.A, 6ru8.2.A, 6ru8.3.A, 6ruu.1.A, 6ruu.1.B, 6ruu.2.A, 6s11.1.A, 6s1f.1.B, 6s1i.4.A, 6s73.1.A, 6s73.2.A, 6s73.3.A, 6s73.4.A, 6s75.1.A, 6s75.2.A, 6s75.3.A, 6s75.4.A, 6s76.1.A, 6s76.2.A, 6s76.3.A, 6s76.4.A, 6s89.1.A, 6s90.1.A, 6s90.2.A, 6s9b.1.A, 6s9c.1.A, 6s9d.1.A, 6s9x.1.A, 6sd9.1.A, 6sdc.1.A, 6sdd.1.A, 6sde.1.A, 6seq.1.A, 6sfk.1.A, 6sfo.1.A, 6sg4.1.A, 6sg4.1.C, 6sgd.1.A, 6so4.1.A, 6soi.1.A, 6sov.1.A, 6spw.1.A, 6srh.1.A, 6srh.2.A, 6sui.1.A, 6sul.1.A, 6sum.1.A, 6sun.1.A, 6t28.1.A, 6t2w.1.A, 6t41.1.A, 6t6a.2.A, 6t6d.3.A, 6t6d.4.A, 6t6f.1.A, 6t6f.2.A, 6t8x.1.A, 6t8x.1.D, 6t8x.1.F, 6tca.1.A, 6tca.1.B, 6tca.2.A, 6tca.2.B, 6tca.3.A, 6tca.3.B, 6tca.4.A, 6tcu.1.A, 6td3.1.B, 6tew.1.A, 6tfp.1.A, 6tfp.2.A, 6tfp.4.A, 6tfu.1.A, 6tfv.2.A, 6tgu.1.A, 6thw.1.A, 6thx.2.A, 6ti8.1.A, 6ti8.4.A, 6tia.1.A, 6tia.2.A, 6tll.1.A, 6tlo.1.A, 6tn9.1.A, 6tnb.1.A, 6tnc.1.A, 6tnd.1.A, 6tpa.1.A, 6tpd.1.A, 6tpe.1.A, 6tpf.1.A, 6tsz.1.A, 6tu9.1.A, 6tua.1.A, 6ty3.1.A, 6ty4.1.A, 6u0k.1.A, 6u2g.1.A, 6u2g.1.B, 6u2h.1.C, 6u2h.1.D, 6u5l.1.A, 6u69.1.A, 6u6a.1.A, 6uan.1.C, 6uan.1.D, 6uip.1.A, 6uip.3.A, 6ul8.1.A, 6umw.1.A, 6una.1.A, 6una.2.A, 6unp.1.A, 6unq.1.A, 6unr.1.A, 6urc.1.A, 6uuo.1.A, 6uuo.1.B, 6v2u.1.A, 6v2u.2.A, 6v34.1.A, 6v34.2.A, 6v5n.1.A, 6v5n.1.B, 6v5n.2.A, 6v5n.2.B, 6v66.2.A, 6v6a.1.A, 6v6k.1.B, 6v6k.2.A, 6v6k.2.B, 6v6k.3.A, 6v6o.4.B, 6v6q.1.A, 6vbz.1.A, 6vbz.2.A, 6vc0.1.A, 6vc0.2.A, 6vc0.3.A, 6vg3.1.A, 6vg3.2.A, 6vg3.3.A, 6vgl.1.A, 6vgl.2.A, 6vhg.1.A, 6vnc.1.A, 6vnc.2.A, 6vne.1.A, 6vne.2.A, 6vng.2.A, 6vno.1.A, 6vov.1.A, 6vp6.1.B, 6vp8.1.A, 6vp8.1.B, 6vpg.1.A, 6vph.1.A, 6vpi.1.A, 6vpj.1.A, 6vpl.1.A, 6vpm.1.B, 6vql.1.A, 6vqm.1.A, 6vre.1.A, 6vrf.1.A, 6vru.1.A, 6vvc.1.A, 6vvd.1.A, 6vvd.2.A, 6vvd.3.A, 6vve.1.A, 6vvg.1.A, 6vvg.1.B, 6vxq.1.A, 6vxr.1.A, 6vzk.1.A, 6w39.1.A, 6w3a.1.A, 6w3a.1.B, 6w3b.1.B, 6w3c.1.A, 6w3c.2.A, 6w3e.1.A, 6w3k.1.A, 6w4o.1.M, 6w7o.1.A, 6w8i.1.A, 6w8i.2.A, 6w8i.3.A, 6w9e.1.A, 6wa2.2.A, 6wa2.2.B, 6wak.1.A, 6wak.2.A, 6wak.2.B, 6whp.1.A, 6wjf.1.A, 6wjf.1.B, 6wpp.1.A, 6wpp.2.A, 6wqx.1.B, 6wqx.2.B, 6wtn.1.A, 6wxj.1.A, 6wxn.1.A, 6wxn.2.A, 6wxn.3.A, 6x5g.1.A, 6x8e.2.A, 6x8g.1.A, 6xag.1.C, 6xbz.1.C, 6xd3.1.C, 6xdb.1.A, 6xdd.2.A, 6xdd.3.A, 6xdf.1.A, 6xdf.1.B, 6xfp.1.B, 6xi8.1.B, 6xih.1.A, 6xih.2.A, 6xka.1.A, 6xl4.1.A, 6xl4.2.A, 6xl4.3.A, 6xl4.4.A, 6xlo.1.B, 6xr6.1.A, 6xr7.1.A, 6xrg.1.A, 6xrl.1.A, 6xrm.1.A, 6xrn.1.A, 6xv9.2.A, 6xx6.1.A,

6xx8.1.A, 6y23.1.A, 6y23.2.A, 6y4x.1.A, 6y6f.1.A, 6y6v.1.A, 6y7c.1.6, 6y7z.1.A, 6y9r.1.A, 6y9s.2.A, 6ya6.1.A, 6ya7.1.A, 6ya8.1.A, 6yat.1.A, 6yfz.1.A, 6yg0.1.A, 6yg1.1.A, 6yg2.1.A, 6yg3.1.A, 6yg4.1.A, 6yg6.1.A, 6yg6.2.A, 6yg7.1.A, 6ygn.1.A, 6ygn.2.A, 6yi8.1.A, 6yi8.2.A, 6yid.1.B, 6yid.2.A, 6ykd.1.A, 6ykg.1.A, 6yky.1.A, 6yky.2.A, 6yky.3.A, 6yky.4.A, 6yl1.1.A, 6ylc.1.A, 6ylk.1.A, 6yll.1.A, 6yll.2.A, 6yoj.1.A, 6yph.1.A, 6yq1.1.A, 6yr9.3.A, 6yt6.1.A, 6yt6.2.A, 6ytd.1.A, 6yty.1.A, 6yul.2.A, 6yvs.2.A, 6yvs.3.A, 6yvs.4.A, 6yvy.1.A, 6yvy.4.A, 6yxs.1.A, 6yxt.1.A, 6yxv.4.A, 6yzh.1.A, 6z0f.1.A, 6z0f.1.B, 6z19.1.A, 6z1c.1.A, 6z1q.1.A, 6z3u.1.B, 6z45.1.A, 6z4b.1.A, 6z4b.2.A, 6z4y.1.A, 6z50.1.A, 6z53.1.A, 6z55.1.A, 6z83.1.A, 6z84.2.A, 6zgc.3.A, 6ziw.1.A, 6zqs.1.A, 6zr5.1.A, 6zr5.2.A, 6zv6.1.8, 6zxd.1.9, 6zxe.1.a, 6zxf.1.8, 6zxg.1.a, 7a2a.2.A, 7a49.2.A, 7a4o.1.A, 7a4q.1.A, 7a5b.2.A, 7a6i.1.A, 7aax.1.A, 7aay.1.A, 7aaz.1.A, 7ab1.1.A, 7aem.1.A, 7aj2.1.A, 7ajm.1.A, 7ajm.2.A, 7ajv.1.A, 7ajy.2.A, 7akm.1.A, 7akm.2.A, 7ako.1.A, 7ako.2.A, 7apf.1.A, 7apg.2.A, 7apj.1.A, 7aqb.1.A, 7aqb.2.A, 7at5.2.A, 7at9.1.A, 7ats.1.A, 7atu.1.A, 7atv.1.A, 7avx.1.A, 7avx.2.A, 7avy.1.A, 7aw4.2.A, 7ax4.1.A, 7ayi.1.A, 7aym.1.A, 7b30.1.A, 7b36.1.A, 7b3m.1.A, 7b3q.1.A, 7b3w.1.A, 7b55.1.A, 7b5l.1.J, 7b5q.1.C, 7b6f.1.A, 7b7s.1.A, 7b85.1.A, 7bcm.1.A, 7be6.1.A, 7bi2.1.A, 7bi4.1.A, 7bi6.1.A, 7bl1.1.B, 7bl1.1.C, 7bmk.1.A, 7bmk.1.B, 7btt.1.A, 7bu4.1.A, 7byk.1.A, 7c2v.1.A, 7c2v.1.B, 7c2w.1.A, 7c2w.1.B, 7c2w.1.C, 7c2w.1.D, 7c3n.1.A, 7cbx.1.A, 7cc2.1.A, 7cc2.2.A, 7cga.1.A, 7cga.2.A, 7cga.3.A, 7cga.4.A, 7chn.1.A, 7cht.1.A, 7cil.1.A, 7cja.1.A, 7clh.1.A, 7cmb.1.A, 7cml.1.A, 7cqe.1.A, 7ctv.1.A, 7ctv.2.A, 7ctx.1.A, 7cy2.1.A, 7cyr.1.A, 7cz2.1.A, 7d57.1.A, 7d57.2.A, 7dd1.1.A, 7dg4.1.A, 7dh3.1.A, 7dhv.1.A, 7dt2.1.A, 7dts.1.A, 7dts.1.B, 7dtz.1.A, 7du8.1.B, 7dua.1.A, 7dv6.1.A, 7dxl.1.A, 7e0z.1.A, 7e11.1.A, 7e34.1.A, 7e73.1.A, 7e75.1.A, 7eec.1.A, 7eed.1.A, 7eef.1.A, 7egc.1.I, 7ejv.1.A, 7ejv.1.B, 7ena.2.A, 7enc.24.A, 7er2.1.A, 7f0a.1.A, 7f0b.1.A, 7f2x.1.A, 7f3g.1.A, 7f3g.1.B, 7f3m.1.A, 7f7w.1.A, 7f7w.2.A, 7fcz.1.A, 7fcz.1.B, 7fd0.1.A, 7feh.1.A, 7fhs.1.A, 7fht.4.A, 7fic.1.A, 7jhg.1.A, 7jhh.1.A, 7jij.1.B, 7jiu.1.A, 7jnt.1.A, 7jou.1.A, 7jov.4.B, 7ju6.1.A, 7ju6.1.B, 7juq.1.A, 7juq.1.B, 7jur.1.B, 7jus.1.A, 7jus.1.B, 7jut.1.B, 7juv.1.B, 7juw.1.A, 7juw.1.B, 7jux.1.A, 7jux.1.B, 7jv7.1.A, 7jw7.1.A, 7jwz.1.A, 7jx0.1.A, 7jxh.1.A, 7jxh.5.A, 7jxl.2.B, 7jxm.2.A, 7jxp.3.A, 7jxq.1.A, 7jxq.2.A, 7jxu.1.A, 7jxx.1.A, 7jxy.1.A, 7jxy.2.A, 7k0v.1.A, 7k0v.1.B, 7k0v.1.C, 7k0v.1.D, 7k1h.1.A, 7k1h.1.B, 7k1h.2.A, 7k1h.2.B, 7k1h.3.B, 7k1i.1.A, 7k6m.1.A, 7k7l.1.A, 7k7z.1.A, 7kac.1.A, 7kac.1.B, 7khj.1.A, 7khj.2.A, 7khk.1.A, 7khk.2.A, 7kia.1.A, 7kja.1.A, 7kja.2.A, 7kjb.1.A, 7kjc.1.A, 7kjc.2.A, 7kjs.1.A, 7kke.1.A, 7kl2.1.A, 7kp6.1.A, 7kp6.2.A, 7kpm.1.A, 7kpv.1.A, 7ksj.1.A, 7kue.1.B, 7kx6.1.A, 7kx6.2.A, 7kx8.1.A, 7kx8.2.A, 7kxl.1.A, 7kxo.1.A, 7kxq.1.A, 7kxw.1.A, 7kxw.2.A, 7kxz.1.A, 7ky0.2.A, 7l24.1.A, 7l24.2.A, 7l25.1.A, 7l26.1.A, 7l26.4.A, 7l5p.1.A, 7l5p.2.A, 7lbm.1.8, 7lg8.1.A, 7lg8.2.A, 7lg8.3.A, 7lg8.4.A, 7lgs.1.A, 7lgs.3.A, 7lht.1.A, 7lhw.1.A, 7li3.1.A, 7ll5.1.A, 7lm2.1.A, 7lqd.1.A, 7lv3.1.A, 7lv3.1.B, 7lvh.1.A, 7lvh.2.A, 7m0k.1.A, 7m0l.1.A, 7m0m.1.A, 7m0m.1.B, 7m0t.1.B, 7m0y.1.B, 7m5z.1.A, 7m5z.2.A, 7m74.1.A, 7mez.1.A, 7mf0.1.A, 7mfd.1.A, 7mfd.1.B, 7mfe.1.A, 7mff.1.C, 7mff.1.D, 7mgj.1.A, 7mgk.4.A, 7mlk.1.A, 7mon.1.A, 7mon.1.B, 7mp8.1.A, 7mt8.1.A, 7mt9.1.A, 7mta.1.A, 7mtb.1.A, 7mx3.1.A, 7mx3.2.A, 7mx3.3.A, 7mx3.4.A, 7mxb.1.A, 7mxb.2.A, 7mxj.1.A, 7mxk.1.A, 7myj.2.A, 7myn.1.A, 7myo.1.B, 7n3u.1.A, 7n4q.1.A, 7n8t.1.A, 7n91.1.A, 7n91.2.A, 7n93.2.A, 7n9g.1.A, 7n9g.2.A, 7naa.1.A, 7nb1.1.A, 7ncf.1.A, 7ng7.1.A, 7nj0.1.B, 7nqw.1.A, 7nry.1.B, 7nti.1.A, 7nur.1.A, 7nvq.1.A, 7nwk.1.A, 7nxj.1.A, 7nxj.2.A, 7nxk.1.A, 7nxk.2.A, 7o2v.1.A, 7o7i.1.A, 7o7j.1.A, 7o7k.1.A, 7o7k.2.A, 7oai.1.A, 7oam.1.A, 7oam.2.A, 7oi4.1.A, 7oox.1.A, 7opm.1.A, 7opo.1.A, 7opo.4.A, 7opo.5.A, 7ore.1.A, 7orf.1.A, 7ote.1.A, 7ote.2.A, 7ovi.1.A, 7ovj.1.A, 7ovm.1.A, 7oxb.1.A, 7oy5.1.A, 7oy6.1.A, 7ozy.1.A, 7ozy.2.A, 7p1l.1.A, 7p3v.1.A, 7p3v.1.B, 7p5z.1.M, 7p7f.1.A, 7p7g.1.A, 7p7g.2.A, 7p7h.1.A, 7p7h.2.A, 7pcd.1.A, 7pg5.1.A, 7pi4.1.D, 7pqv.1.A, 7psu.1.A, 7pt6.1.O, 7pt7.1.G, 7pue.1.A, 7pwd.1.A, 7q4a.1.A, 7q4a.1.B, 7q52.1.A, 7q6h.1.A, 7q7i.1.A, 7q7l.1.A, 7q8v.1.A, 7q8y.1.A, 7q8y.2.A, 7qb2.1.A, 7qg2.1.A, 7qg2.4.A, 7qg3.1.A, 7qg5.2.A, 7qgp.1.A, 7qhw.1.A, 7qhw.2.A, 7qq6.1.A, 7qq6.1.B, 7qq6.2.A, 7qq6.2.B, 7que.1.A, 7que.2.A, 7quf.1.A, 7qux.1.A, 7qwk.1.A, 7qwk.5.A, 7qwk.6.A, 7r9l.1.B, 7r9n.1.B, 7r9p.1.A, 7r9p.1.B, 7r9t.1.A, 7r9t.1.B, 7ree.1.A, 7rj8.1.A, 7rn6.1.A, 7rsj.1.A, 7rsp.1.A, 7run.1.A, 7rwf.1.A, 7s25.1.A, 7s25.1.B, 7s25.2.A, 7s25.2.B, 7s26.1.A, 7s26.1.B, 7s26.2.A, 7s26.2.B, 7s3l.1.A, 7s46.1.A, 7s47.1.A, 7s4t.1.A, 7s6u.1.A, 7s7a.1.A, 7shv.1.A, 7siu.1.A, 7siu.1.B, 7sj3.1.A, 7sqm.1.A, 7szc.1.A, 7szd.1.A, 7t0p.1.A, 7t3x.1.A, 7t4k.1.A, 7t4k.1.B, 7t4l.1.B, 7t4m.1.A, 7t4m.1.B, 7t6f.1.A, 7tnh.1.A, 7tvd.1.A, 7tz7.1.B, 7u2z.1.A, 7u2z.2.A, 7u31.1.A, 7u9a.1.A, 7uiq.2.A, 7ujp.1.A, 7ujr.1.A, 7ujs.1.A, 7ukv.1.A, 7ukw.1.A, 7ukw.1.B, 7ukz.1.A, 7up4.1.A, 7up4.2.A, 7up6.1.A, 7upm.1.A, 7uxi.1.A, 7uxk.1.A, 7uy0.1.A, 7uy0.2.A, 7uy3.1.A, 7uyr.1.A, 7uyv.1.A, 7uyv.3.A, 7uyw.1.A, 7v3r.1.A, 7v3v.1.M, 7vbt.1.A, 7vdp.1.A, 7vdp.2.A, 7vdq.1.A, 7vdu.1.A, 7vjl.1.A, 7vjl.2.A, 7vkm.1.A, 7vkn.1.A, 7vko.1.A, 7vnx.1.A, 7w19.1.A, 7w19.2.A, 7w5c.1.A, 7w5o.1.A, 7w5o.2.A, 7w7x.1.A, 7w7x.1.B, 7w7y.1.A, 7w7y.1.B, 7wcl.1.A, 7wcl.2.A, 7wcw.1.A, 7wcx.1.A, 7wj6.1.A, 7wj6.1.B, 7wtt.1.6, 7wu0.1.8, 7xaf.1.A, 7xsv.1.A, 7xzq.1.A, 7xzr.1.A, 7xzr.2.A, 7y1g.1.A, 7y1g.2.A, 7y4u.1.A, 7ybo.1.A, 7z37.1.C, 7z38.1.C, 7z4v.1.A, 7z5w.1.A, 7z61.1.A, 7z75.1.A, 7zh8.1.A, 7zh8.3.A, 7zr0.1.D, 7zr5.1.D, 7zr6.1.D, 7zvs.1.A, 7zvs.2.A, 7zvs.3.A, 8a27.1.A, 8a2a.1.A, 8a2b.1.A, 8a2d.1.A, 8a5j.1.A, 8a66.1.A, 8a8m.1.A, 8a8m.1.B, 8aau.1.A, 8ahg.1.A, 8ahp.1.A, 8an8.1.A, 8ans.1.A, 8aof.1.A, 8arj.1.A, 8aza.1.B, 8aza.1.C, 8bem.1.A, 8c01.1.3, 8d6f.1.A, 8d73.1.A, 8dcp.1.A, 8dcx.1.A, 8dd4.1.A, 8dd8.1.A, 8deg.1.A, 8dks.1.A, 8e1x.1.A, 8e1x.1.B, 8ej4.1.K, 8eqd.1.A, 8exl.1.A, 8gua.1.A, 8gub.1.A, 8gud.1.A, 8gxq.48.A
